# Supplementary material for: Polε Instability Drives Replication Stress, Abnormal Development, and Tumorigenesis
Source: Mol Cell. 2018 May 17;70(4):707–721.e7. doi: 10.1016/j.molcel.2018.04.008 (PMC5972231; doi:10.1016/j.molcel.2018.04.008)
Supplement: Document S1. Figures S1–S6 [file mmc1.pdf]

Molecular Cell, Volume *70*

Supplemental Information

Polε Instability Drives Replication Stress, Abnormal Development, and Tumorigenesis

Roberto Bellelli, Valerie Borel, Clare Logan, Jennifer Svendsen, Danielle E. Cox, Emma Nye, Kay Metcalfe, Susan M. O'Connell, Gordon Stamp, Helen R. Flynn, Ambrosius P. Snijders, François Lassailly, Andrew Jackson, and Simon J. Boulton

Figure S1

A.

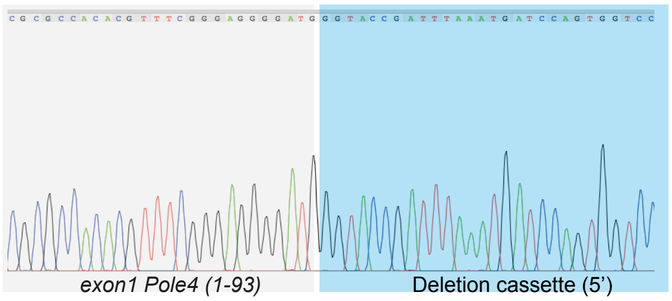

B.

	Pole4 (inbred)			
	+/+	+/-	-/-	Total
#	91 (63)	161 (127)	1 (63)	253
%	36.0 (25)	63.6 (50)	0.4 (25)	100

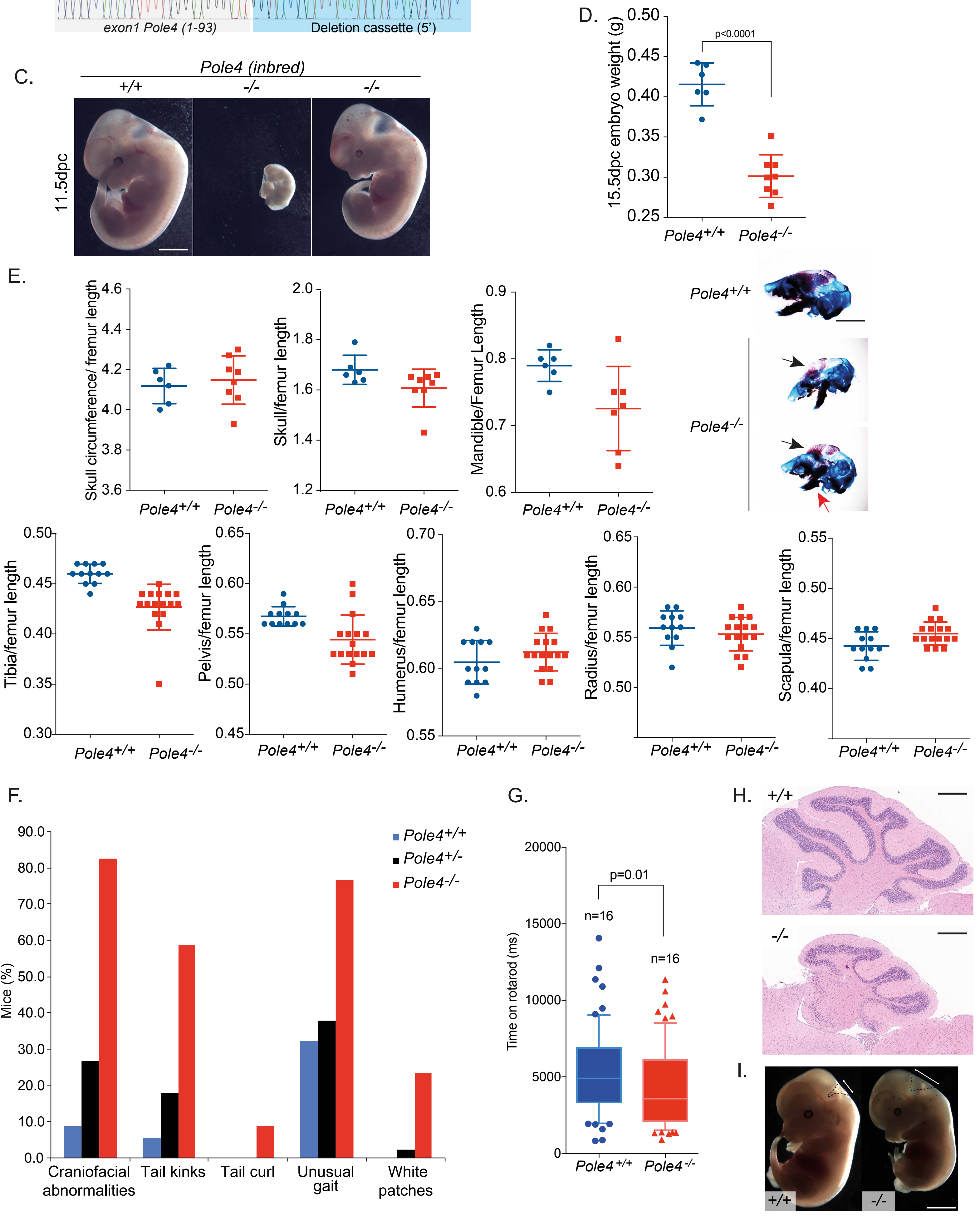

Figure S1: (Related to Figure 1)

(A) Sequencing data showing the insertion of the deletion cassette in exon1 of *Pole4* gene. (B) *Pole4*$^{+/+}$, *Pole4*$^{+/-}$ and *Pole4*$^{-/-}$ mice Mendelian ratios in C57BL/6 background. Numbers in brackets represent expected number or percent. (C) Representative images of inbred *Pole4*$^{+/+}$ and *Pole4*$^{-/-}$ embryos at 11.5dpc. Bar=0.5mm (D) Weight analysis of *Pole4*$^{+/+}$ and *Pole4*$^{-/-}$ embryos at 15.5dpc. Error bars represent ±standard error of the mean (SEM) of n= *Pole4*$^{+/+}$ and n= *Pole4*$^{-/-}$. Significance: t-test, p<0.0001. (E) Body and bones measurements relative to femur length during development in utero at 15.5dpc. At least 3 embryos were used per analysis. Alcian blue/Alizarin red staining of *Pole4*$^{+/+}$ and *Pole4*$^{-/-}$ embryos head at 15.5dpc. The black arrow shows the bulging forehead and the red arrow indicates the shortening of the mandible. Bar=3mm. (F) Frequency of *Pole4*$^{+/+}$ and *Pole4*$^{-/-}$ mice displaying abnormal phenotypes such as craniofacial abnormalities, tail kinks or curl, unusual gait and white patches. (G) Rotarod experiment testing 12 months old *Pole4*$^{+/+}$ and *Pole4*$^{-/-}$ mice coordination. Note the decreased time spent on the rotating rod by *Pole4*$^{-/-}$ mice compared to their wild type littermates. Error bars represent ±standard error of the mean (SEM) of n=16 *Pole4*$^{+/+}$ and n=16 *Pole4*$^{-/-}$. Significance: *t*-test, p=0.01. (H) Sagittal section of cerebellum from *Pole4*$^{+/+}$ and *Pole4*$^{-/-}$ mice. Note the reduced size and decrease in the number of lobule in the mutant section. Sections are stained by hematoxylin and eosin, bar=500µm. (I) Whole-mount lateral images of embryos at 13.5dpc. White arrows show the expanded roof of fourth ventricle, bar=1mm.

Figure S2

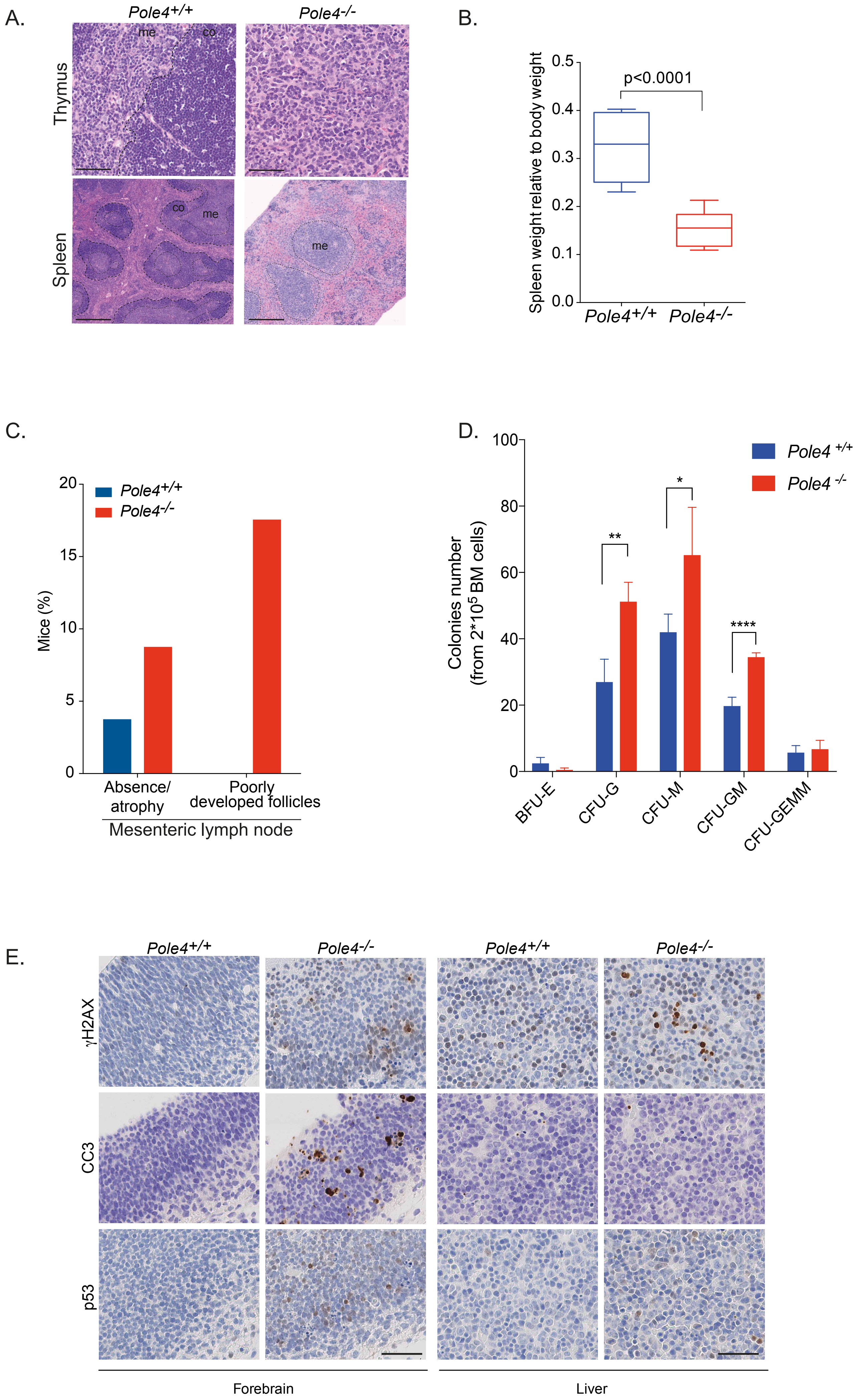
A.
Pole4+/+
Pole4-/-
Thymus
Spleen
me
co
B.
Spleen weight relative to body weight
p<0.0001
0.5
0.4
0.3
0.2
0.1
0.0
Pole4+/+
Pole4-/-
C.
Mice (%)
20
15
10
5
0
Pole4+/+
Pole4-/-
Absence/ atrophy
Poorly developed follicles
Mesenteric lymph node
D.
Colonies number (from 2*10^5 BM cells)
100
80
60
40
20
0
Pole4 +/+
Pole4 -/-
**
*
****
BFU-E
CFU-G
CFU-M
CFU-GM
CFU-GEMM
E.
Pole4+/+
Pole4-/-
Pole4+/+
Pole4-/-
γH2AX
CC3
p53
Forebrain
Liver

Figure S2: (Related to Figure 2)

(A) Representative sagittal section of *Pole4*$^{+/+}$ and *Pole4*$^{-/-}$ mice thymus and spleen. me: medulla, co: cortex. Bar= 300µm for thymus and 200µm for spleen. (B) Spleen weight relative to body weight. Significance: *t*-test, p<0.0001. (C) Frequency of *Pole4*$^{+/+}$ and *Pole4*$^{-/-}$ mice presenting abnormal mesenteric lymph nodes. (D) Cobblestone area forming cells assay of bone marrow (BM) cells from *Pole4*$^{+/+}$ and *Pole4*$^{-/-}$ mice plated and grown in Methocult media. Colonies of hematopoietic progenitors BFU-E, CFU-GEMM, CFU-GM, CFU-G or CFU-M were counted 12 days after plating $2x10^5$ cells. Bone marrow from 3 animals was used and plated in triplicates. Significance: *t*-test, for CFU-GM, ****: p<0.0001; for CFU-G, **: p=0.0016; for CFU-M, *p=0.0232. (E) Representative images of γH2AX, Cleaved caspase 3 and p53 immunohistochemistry in forebrain and liver section of 13.5dpc embryos. Nuclei have been counterstained with hematoxylin. Bar=50µm.

Figure S3

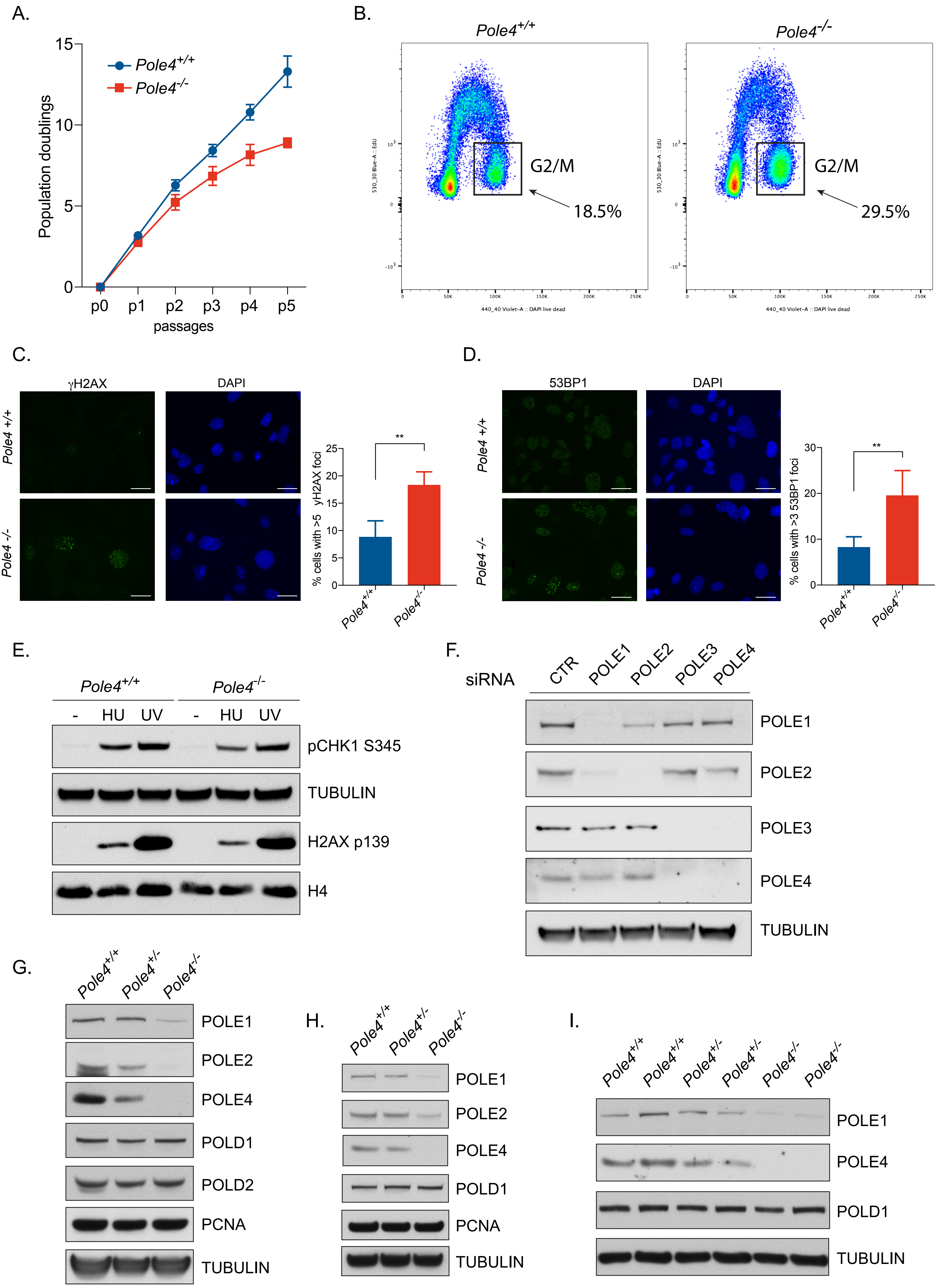

A.
Population doublings
passages
p0
p1
p2
p3
p4
p5
Pole4+/+
Pole4-/-
B.
Pole4+/+
Pole4-/-
G2/M
18.5%
29.5%
C.
γH2AX
DAPI
Pole4 +/+
Pole4 -/-
% cells with >5 γH2AX foci
**
D.
53BP1
DAPI
% cells with >3 53BP1 foci
E.
- HU UV
pCHK1 S345
TUBULIN
H2AX p139
H4
F.
siRNA
CTR
POLE1
POLE2
POLE3
POLE4
TUBULIN
G.
Pole4+/-
POLD1
POLD2
PCNA
H.
I.

Figure S3. (Related to Figure 3).

(A) Analysis of proliferation of *Pole4*$^{+/+}$ and *Pole4*$^{-/-}$ MEFs, measured as accumulation of population doublings. Cells were cultured in low oxygen conditions (5%) according to a standard 3T3 protocol; data are shown as mean +/- SD of 4 different experiments. (B) Representative EdU/DAPI flow cytometry profile of *Pole4*$^{+/+}$ and *Pole4*$^{-/-}$ MEFs. (C) Left: representative γH2AX immunofluorescence staining of *Pole4*$^{+/+}$ and *Pole4*$^{-/-}$ MEFs. DAPI was used for nuclear counterstaining. Right: Bar graph showing percentage of *Pole4*$^{+/+}$ and *Pole4*$^{-/-}$ MEFs with >5 γH2AX foci (** $p< 0.01$); Error bars ±standard deviation (SD) are included; Scale bar, 30µM. (D) representative 53BP1 immunofluorescence staining of *Pole4*$^{+/+}$ and *Pole4*$^{-/-}$ MEFs. DAPI was used for nuclear counterstaining. Right: Bar graph showing percentage of *Pole4*$^{+/+}$ and *Pole4*$^{-/-}$ MEFs with >3 53BP1 foci (** $p< 0.01$); Error bars ±standard deviation (SD) are included; Scale bar, 30µM. (E) Western blot analysis of total cell extracts from *Pole4*$^{+/+}$ and *Pole4*$^{-/-}$ MEFs exposed to 2 mM Hydroxyurea (HU) or 20J/m2 ultraviolet rays (UV) for 4 hours. pCHK1 S345 and γH2AX (pH2AX S139) were used as markers of checkpoint activation. Tubulin and H4 were used as loading controls. (F) Western blot analysis of HeLa cell lysates, subjected to silencing of Polε complex subunits, using the indicated antibodies. Tubulin was used for normalization. (G-H-I) Western blot analysis of testis (G), embryo (H) or MEFs (I) extracts from *Pole4*$^{+/+}$, *Pole4*$^{+/-}$ and *Pole4*$^{-/-}$ mice using the indicated antibodies. Tubulin was used as loading control.

Figure S4

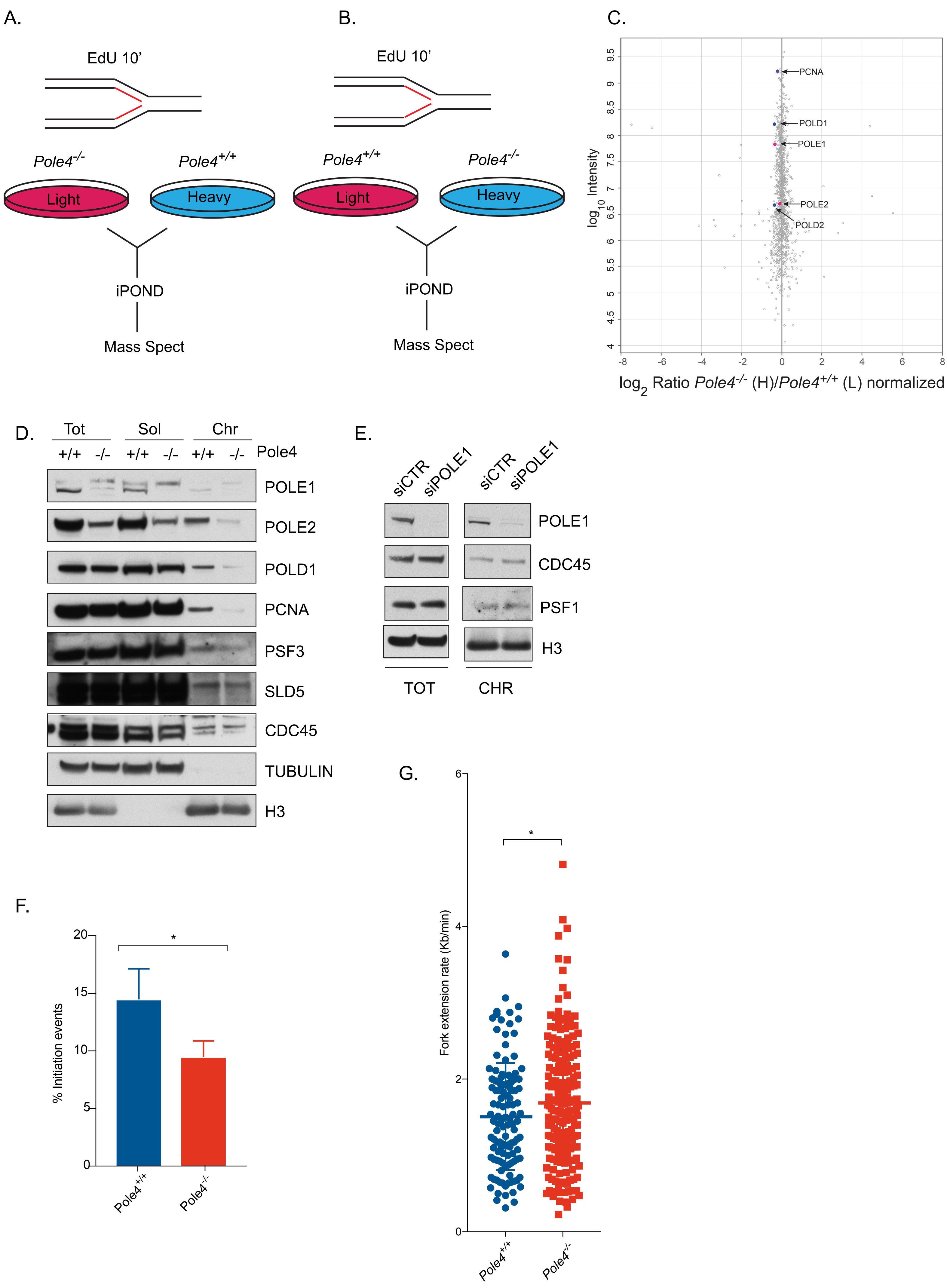
A.
EdU 10'
Pole4-/-
Pole4+/+
Light
Heavy
iPOND
Mass Spect
B.
EdU 10'
Pole4+/+
Pole4-/-
Light
Heavy
iPOND
Mass Spect
C.
log10 Intensity
PCNA
POLD1
POLE1
POLE2
POLD2
log2 Ratio Pole4-/- (H)/Pole4+/+ (L) normalized
D.
Tot
Sol
Chr
+/+
-/-
Pole4
POLE1
POLE2
POLD1
PCNA
PSF3
SLD5
CDC45
TUBULIN
H3
E.
siCTR
siPOLE1
POLE1
CDC45
PSF1
H3
TOT
CHR
F.
% Initiation events
Pole4+/+
Pole4-/-
*
G.
Fork extension rate (Kb/min)
Pole4+/+
Pole4-/-
*

Figure S4. (Related to Figure 3).

(A) and (B) Cartoons depicting the iPOND-SILAC-MS strategy used for replisome analysis of *Pole4*$^{-/-}$ and *Pole4*$^{+/+}$ MEFs (C) Results of the iPOND-SILAC-MS experiment reported as logarithmic fold change of Heavy/Light ratio. Polδ and Polε major subunits are indicated in the plot as blue and red dots respectively. (D) Western blot analysis of replication proteins from total, soluble and chromatin fractions of *Pole4*$^{+/+}$ and *Pole4*$^{-/-}$ MEFs obtained from inbred C57BL/6 embryos. Tubulin and Histone H3 were used as loading controls (E) Western blot analysis of replication proteins from total (TOT) and chromatin (CHR) fractions of HeLa cells transfected with siRNAs against POLE1. Histone H3 was used as loading control. (F) Bar graph showing the percentage of initiation events in *Pole4*$^{+/+}$ and *Pole4*$^{-/-}$ MEFs. Data were obtained from 3 different *Pole4*$^{+/+}$ and *Pole4*$^{-/-}$ MEFs clones (*$p<0.05$); Error bars ±standard deviation (SD) are included. (G) Replication fork speed (IdU extension rate) of *Pole4*$^{+/+}$ and *Pole4*$^{-/-}$ MEFs in a C57BL/6 genetic background (*$p<0.05$).

Figure S5

A.

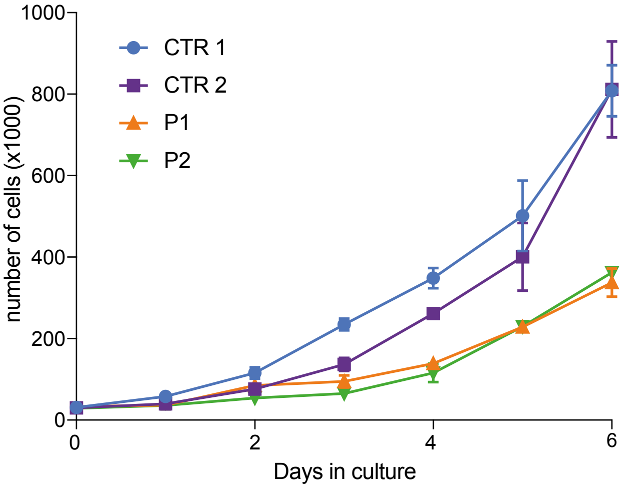

B.

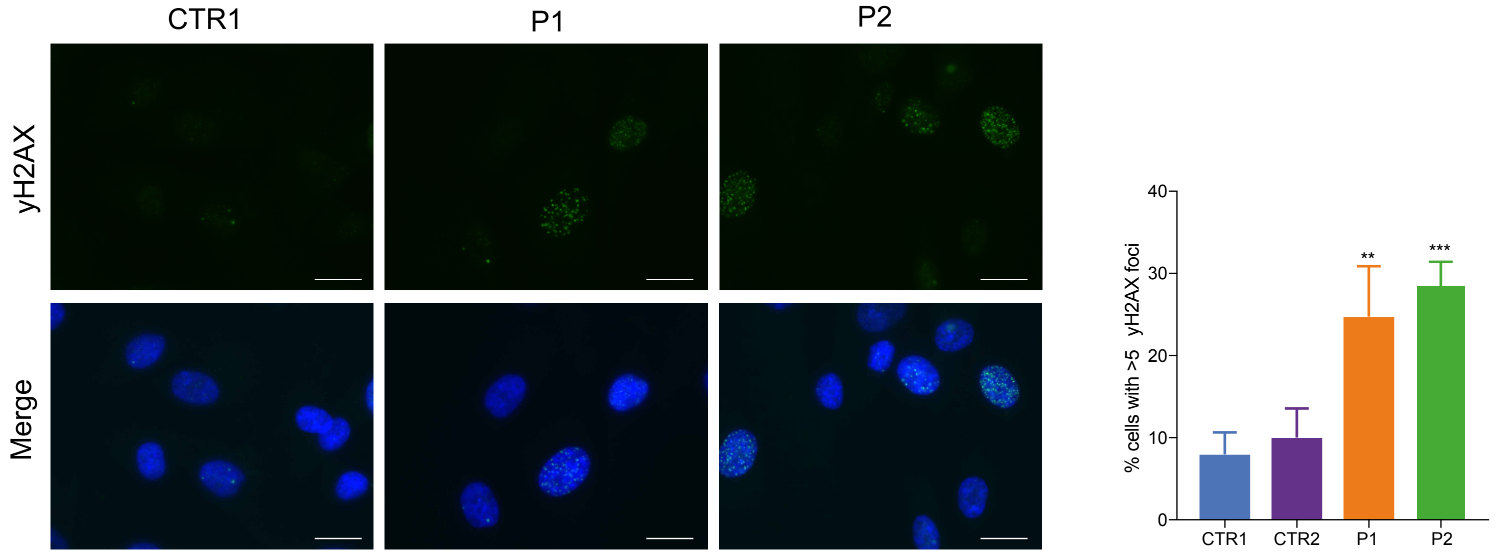

C.

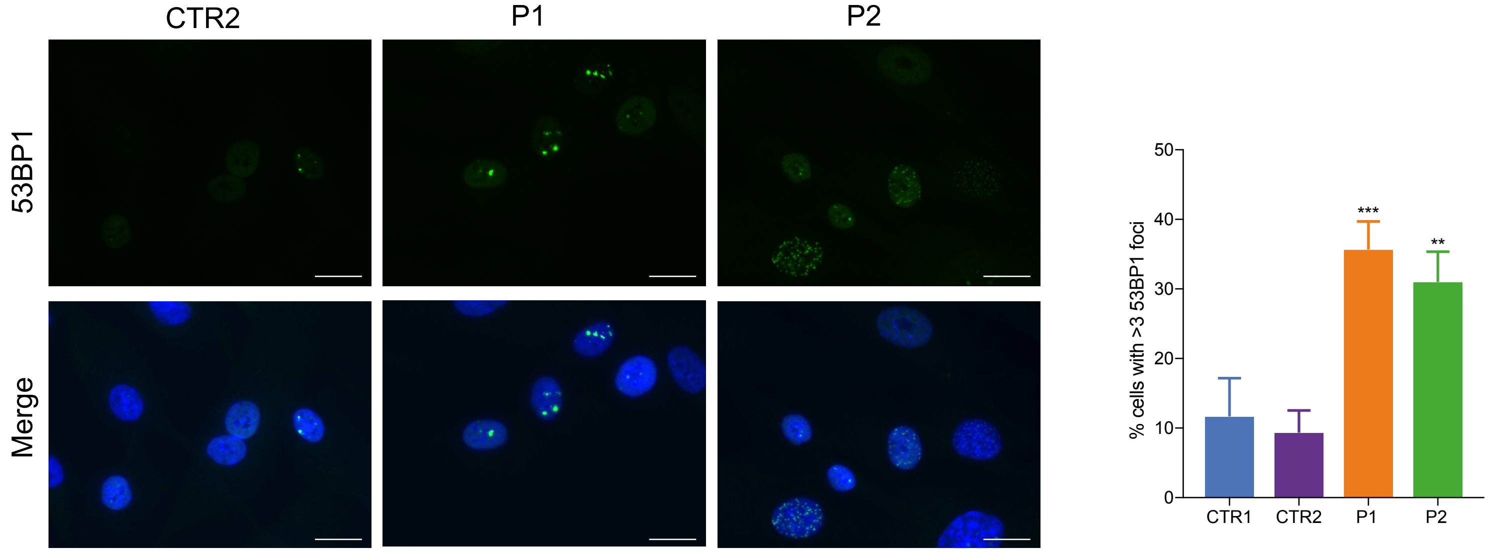

Figure S5. (Related to figure 4).

(A) Growth curve of *POLE1* mutant (P1 and P2) and control (CTR1 and CTR2) cells. 20.000 cells were plated in duplicate and counted for 6 consecutive days; Error bars ±standard deviation (SD) are included. (B) Left: representative γH2AX immunofluorescence staining of CTR1 and *POLE1* mutant (P1 and P2) cells. DAPI was used for nuclear counterstaining. Right: Bar graph showing percentage of CTR and *POLE1* mutant cells with >5 γH2AX foci (** $p < 0.01$; *** $p < 0.001$); Error bars ±standard deviation (SD) are included; Scale bar, 30μM. (C) Left: representative 53BP1 immunofluorescence staining of CTR1 and *POLE1* mutant (P1 and P2) cells. DAPI was used for nuclear counterstaining. Right: Bar graph showing percentage of CTR and *POLE1* mutant cells with >3 53BP1 foci (** $p < 0.01$; *** $p < 0.001$); Error bars ±standard deviation (SD) are included; Scale bar, 30μM.

Figure S6

A.

Genotype	Observed #	Observed %	Expected #	Expected %
Pole4$^{+/+}$ p53$^{+/+}$	5	4.6	6.8	6.25
Pole4$^{+/+}$ p53$^{+/-}$	20	18.3	13.6	12.5
Pole4$^{+/+}$ p53$^{-/-}$	3	2.8	6.8	6.25
Pole4$^{+/-}$ p53$^{+/+}$	14	12.8	13.6	12.5
Pole4$^{+/-}$ p53$^{+/-}$	43	39.4	27.3	25
Pole4$^{+/-}$ p53$^{-/-}$	9	8.3	13.6	12.5
Pole4$^{-/-}$ p53$^{+/+}$	0	0	6.8	6.25
Pole4$^{-/-}$ p53$^{+/-}$	***11***	***10.1***	***13.6***	***12.5***
Pole4$^{-/-}$ p53$^{-/-}$	***4***	***3.7***	***6.8***	***6.25***
Total	109	100	109	100

B.

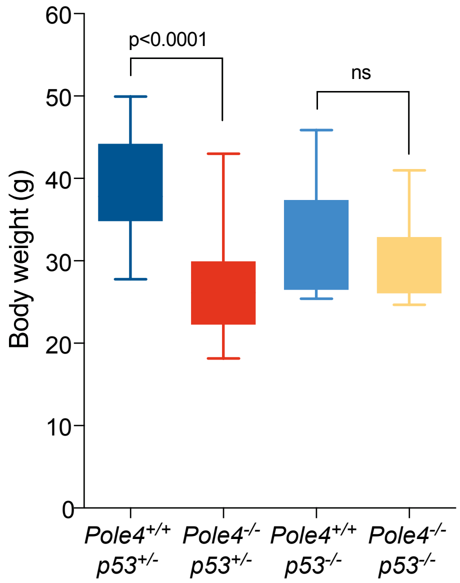

C.

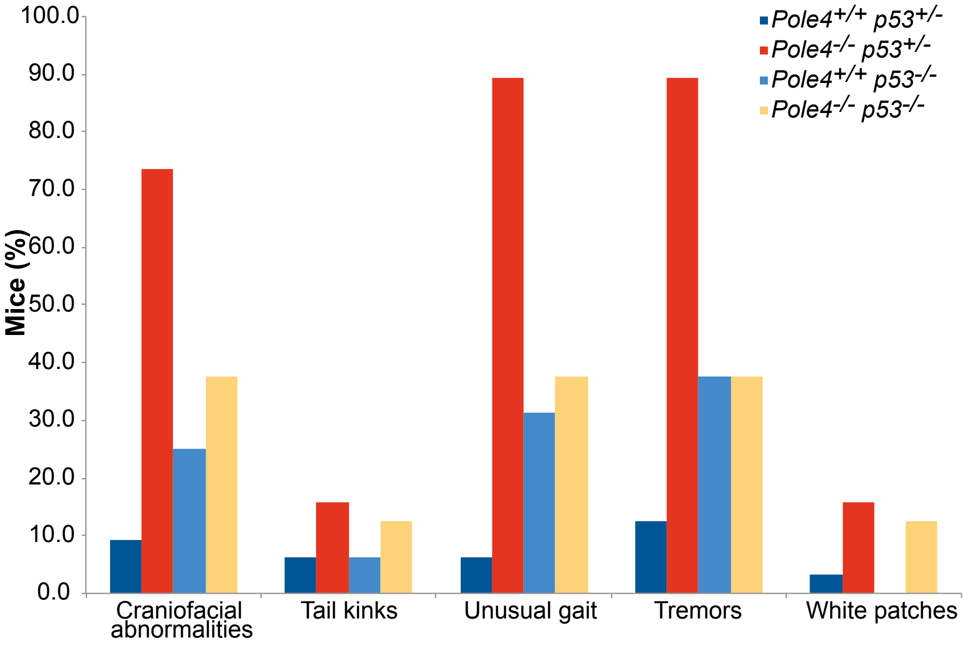

Figure S6: (Relative to Figure 6)

(A) *Pole4/p53* mice Mendelian ratios in C57BL/6 background. (B) *Pole4/p53* mice body weight at 6 months. Significance: *t*-test. (C) Frequency of *Pole4/p53* mice displaying abnormal phenotypes such as craniofacial abnormalities, tail kinks or curl, unusual gait, tremors and/or white patches.
