# Supplementary material for: Polε Instability Drives Replication Stress, Abnormal Development, and Tumorigenesis
Source: Mol Cell. 2018 May 17;70(4):707–721.e7. doi: 10.1016/j.molcel.2018.04.008 (PMC5972231; doi:10.1016/j.molcel.2018.04.008)
Supplement: Document S2. Article plus Supplemental Information [file mmc2.pdf]

Molecular Cell

Article

Polε Instability Drives Replication Stress, Abnormal Development, and Tumorigenesis

Graphical Abstract

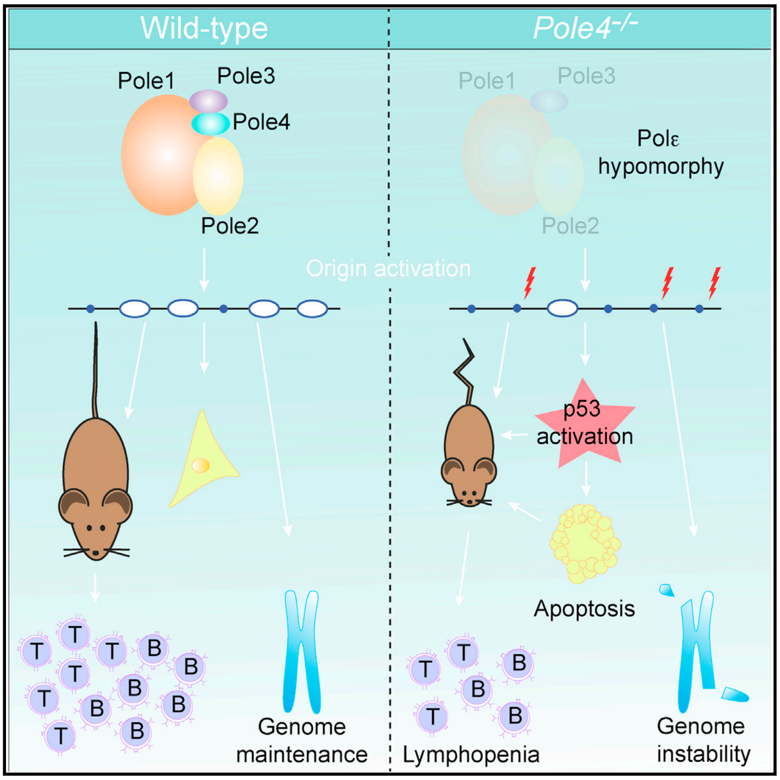

Highlights

- *Pole4*$^{-/-}$ mice exhibit impaired development and defective lymphocyte maturation
- Pole4 is crucial for maintaining the stability of the Polε complex
- Polε hypomorphy causes replication stress via inefficient origin activation
- p53 inactivation rescues all major developmental defects in *Pole4*-deficient mice

Authors

Roberto Bellelli, Valerie Borel, Clare Logan, ..., François Lassailly, Andrew Jackson, Simon J. Boulton

Correspondence

simon.boulton@crick.ac.uk

In Brief

Bellelli et al. report that *Pole4* deficiency in mice or *POLE1* mutations in humans result in Pol Epsilon complex instability, replication stress, and inefficient origin activation. As a consequence, Pol Epsilon hypomorphic mice exhibit aberrant growth and development, lymphopenia, and tumor predisposition, which can be rescued by deleting p53.

Bellelli et al., 2018, Molecular Cell *70*, 707–721
May 17, 2018 © 2018 The Author(s). Published by Elsevier Inc.
https://doi.org/10.1016/j.molcel.2018.04.008

CellPress

Polε Instability Drives Replication Stress, Abnormal Development, and Tumorigenesis

Roberto Bellelli,[1,5] Valerie Borel,[1,5] Clare Logan,[2] Jennifer Svendsen,[1] Danielle E. Cox,[1] Emma Nye,[1] Kay Metcalfe,[3] Susan M. O'Connell,[4] Gordon Stamp,[1] Helen R. Flynn,[1] Ambrosius P. Snijders,[1] François Lassailly,[1] Andrew Jackson,[2] and Simon J. Boulton[1,6,*]
[1]The Francis Crick Institute, 1 Midland Road, London NW1 1AT, UK
[2]MRC Institute of Genetics & Molecular Medicine, The University of Edinburgh, Western General Hospital, Crewe Road, Edinburgh EH4 2XU, UK
[3]Department of Genetic Medicine, St Mary's Hospital, Oxford Road, Manchester, M13 OJH, UK
[4]Department of Paediatrics, Cork University Hospital, Wilton, Cork T12 DC4A, Ireland
[5]These authors contributed equally
[6]Lead Contact
*Correspondence: simon.boulton@crick.ac.uk
https://doi.org/10.1016/j.molcel.2018.04.008

SUMMARY

DNA polymerase ε (POLE) is a four-subunit complex and the major leading strand polymerase in eukaryotes. Budding yeast orthologs of POLE3 and POLE4 promote Polε processivity *in vitro* but are dispensable for viability *in vivo*. Here, we report that POLE4 deficiency in mice destabilizes the entire Polε complex, leading to embryonic lethality in inbred strains and extensive developmental abnormalities, leukopenia, and tumor predisposition in outbred strains. Comparable phenotypes of growth retardation and immunodeficiency are also observed in human patients harboring destabilizing mutations in POLE1. In both *Pole4*$^{-/-}$ mouse and *POLE1* mutant human cells, Polε hypomorphy is associated with replication stress and p53 activation, which we attribute to inefficient replication origin firing. Strikingly, removing p53 is sufficient to rescue embryonic lethality and all developmental abnormalities in *Pole4* null mice. However, *Pole4*$^{-/-}$*p53*$^{+/-}$ mice exhibit accelerated tumorigenesis, revealing an important role for controlled CMG and origin activation in normal development and tumor prevention.

INTRODUCTION

DNA replication in eukaryotes is performed by a large multi-subunit machine, known as the replisome, which assembles at thousands of DNA replication origins in a cell-type- and developmental-stage-dependent manner (for review, see Méchali, 2010). Assembly of the eukaryotic replisome begins in G1 phase of the cell cycle with loading of inactive MCM2–7 double hexamers at origins (for review, see Masai et al., 2010). At the G1-S transition, DDK- and CDK-dependent phosphorylation drives assembly of the CMG complex, composed of CDC45, MCM2–7, and GINS1–4, which is considered to form the processive replicative helicase (Ilves et al., 2010; Labib, 2010). Two major DNA polymerases, Polδ and Polε, are thought to replicate the lagging and leading strand, respectively (Nick McElhinny et al., 2008; Pursell et al., 2007). In addition to its catalytic role, Polε is required for CMG formation and origin activation in budding yeast; while its N-terminal catalytic domain is dispensable for viability, deletion of its C-terminal structural domain is lethal in *S. cerevisiae* (Handa et al., 2012; Muramatsu et al., 2010; Sengupta et al., 2013; Yeeles et al., 2015).

Mammalian Polε is composed of four subunits: POLE1, homolog of *S. cerevisiae* Pol2, contains polymerase and exonuclease activities (Lee et al., 1991; Syvaoja and Linn, 1989); POLE2, homolog of Dpb2, structurally links Polε to the CMG complex (Li et al., 1997; Sengupta et al., 2013); and two smaller subunits, POLE3 and POLE4, orthologs of Dpb4 and Dpb3, whose functions are unknown (Li et al., 2000). Dpb3 and Dpb4 are non-essential in *S. cerevisiae*, with *dpb3*Δ and *dpb4*Δ yeast strains exhibiting increased mutation rates and defective S-phase progression, respectively (Araki et al., 1991; Ohya et al., 2000). Both proteins harbor histone fold motifs of the H2A-H2B family, through which they are thought to interact with each other and increase Polε binding to double-stranded DNA and/or provide an interaction surface at the replication fork (Iida and Araki, 2004; Tackett et al., 2005; Tsubota et al., 2003). Of note, Dpb4 and its human homolog POLE3 are also components of the budding yeast ISWI2/yCHRAC and the human hCHRAC chromatin remodeling complexes, respectively (Poot et al., 2000; Iida and Araki, 2004). Studies *in vitro* using recombinant proteins have shown that yeast Polε lacking Dpb3 and Dpb4 exhibits reduced processivity on synthetic DNA substrates, which may explain the increased mutagenesis of *dpb3*Δ-*dpb4*Δ yeast strains (Aksenova et al., 2010). Biochemical studies using reconstituted human Polε suggested that POLE3 and POLE4 bind to the catalytic subunit POLE1 at different sites compared to their yeast homologs and do not significantly contribute to the rate of DNA synthesis *in vitro*, which suggests that POLE3-POLE4 functions in higher eukaryotes might differ from budding yeast (Bermudez et al., 2011).

This is an open access article under the CC BY license (http://creativecommons.org/licenses/by/4.0/).

Several clinically recognized human syndromes are caused by mutations in DNA replication genes, many of them presenting with growth failure and skeletal abnormalities, endocrine and/or immune dysfunctions, and heightened cancer risk (for review, see Jackson et al., 2014). Recently, mutations of Polε subunits, POLE1 and POLE2, have been described in human patients suffering from immunodeficiency and growth restriction (Frugoni et al., 2016; Pachlopnik Schmid et al., 2012). However, substantive biological and mechanistic insights into disease pathogenesis remain to be established. Moreover, mutations of *POLE1* in proximity to the proofreading exonuclease domain have been identified in several neoplasias such as colorectal and endometrial cancer, which are associated with a peculiar hypermutator phenotype (Alexandrov et al., 2013; Campbell et al., 2017; Kandoth et al., 2013; Yang et al., 2013).

To unravel the function of Polε accessory subunits in vertebrates, we report here the generation of a mouse knockout for the *Pole4* gene and describe the functional characterization of primary cell lines from two human patients harboring destabilizing mutations in *POLE1*. While essential for embryonic development in a C57BL/6 inbred strain, we found that *Pole4* knockout mice are viable in an outbred background, presenting with a multitude of developmental growth defects, including craniofacial and skeletal abnormalities and defective B and T cell maturation. Notably, these overlap with the clinical features observed in patients harboring *POLE1/2* mutations. In addition, we show that *Pole4*$^{-/-}$ mice and patient cells with *POLE1* mutations exhibit Polε complex instability, which leads to inefficient origin activation, replicative damage, genome instability, and p53 activation. Surprisingly, removal of p53 is sufficient to rescue the complex array of developmental abnormalities in *Pole4*$^{-/-}$ mice and restore lymphoid lineage differentiation. Despite this, p53 haploinsufficiency further exacerbates genetic instability and leads to accelerated tumorigenesis in the absence of POLE4. Collectively, our work defines a mouse model of Polε hypomorphy and extends the group of human genetic diseases at the cross-road of CMG activation and replication fork establishment, highlighting the intimate connection between replication origin activation, genome instability, and cancer development.

RESULTS

Pole4 Knockout Mice Are Viable in an Outbred Strain and Exhibit Phenotypes Similar to Human Patients with Mutations in *POLE1* or *POLE2*

To investigate the role of DNA polymerase ε in a complex organism, we generated a *Pole4*-deficient mouse model in the inbred C57BL/6 genetic background from an existing EUCOMM embryonic stem cell line, *Pole4*$^{tm1(KOMP)Vlcg}$, in which most of the *Pole4* gene has been replaced by a β-galactosidase-neomycin reporter cassette (Figure 1A). Sequencing of the first exon revealed that the deletion cassette is inserted 94 bp after the start of exon 1 (Figure S1A). Based on this information, we developed a genotyping strategy to detect wild-type (WT), heterozygous, and mutant alleles using a three-primer PCR. POLE4 protein was undetectable in mutant embryo extracts by immunoblotting and was reduced by ~50% in heterozygous cells compared to WT (Figure 1B).

Pole4$^{tm1(KOMP)Vlcg}$ intercrosses in the C57BL/6 inbred background gave rise to only 1 viable animal out of 63 expected (Figure S1B). Further examination of *Pole4* embryos *in utero* revealed that most embryos die with extensive developmental defects between 11.5 and 13.5 days postcoitum (dpc) (Figure S1C; data not shown). Unexpectedly, crossing the *Pole4*$^{tm1(KOMP)Vlcg}$ allele into an FVB/sv129 outbred background resulted in viable *Pole4* null mutant mice, albeit at lower than expected Mendelian ratios, corresponding to 9.3% compared to 25% expected (Figure 1C). *In utero* analysis of timed matings revealed that *Pole4* lethality in the FVB/sv129 outbred background occurs between 13.5 dpc and birth. *Pole4* mutant embryos in this outbred background showed no significant developmental abnormalities at 13.5 dpc with the exception of intrauterine growth retardation (Figure 1D, left). At 13.5 and 15.5 dpc, mutant embryos were significantly smaller with a decreased weight (Figures 1D, middle, and S1D; at 13.5 dpc, *Pole4*$^{+/+}$, 0.16 ± 0.022 g versus *Pole4*$^{-/-}$, 0.12 ± 0.027 g; at 15.5 dpc, *Pole4*$^{+/+}$, 0.42 ± 0.011 g versus *Pole4*$^{-/-}$, 0.30 ± 0.0094 g, respectively). They also exhibited a significantly smaller mandible, tibia, and pelvis length relative to the femur length, whereas skull, humerus, and radius had a similar size compared to WT (Figure S1E). From 3 to 34 weeks postpartum, *Pole4* mutants remained at lower weight and shorter length compared to their WT littermates (Figure 1D, right; data not shown), suggesting that these mice have a significant developmental growth defect. To investigate the source of these developmental issues, we proceeded to characterize the viable FVB/sv129 outbred *Pole4*$^{-/-}$ animals.

Breeding experiments over a 5-month period involving a *Pole4*$^{-/-}$ animal as one of the breeding pairs showed that these mice are subfertile, producing significantly less litters and a tendency for fewer pups (Figure 1E, top). Breeding of heterozygous pairs resulted in an average of 26 litters with 202 pups (1.3 litters per 21-day-gestation interval and 11 pups per litters), whereas breeding involving one mutant in a pair produced only 15 litters with 118 pups (0.95 litters per 21-day-gestation interval and 7.5 pups per litters). Examination of the testes of *Pole4*-deficient neonatal mice revealed a marked reduction in the number of promyelocytic leukemia zinc-finger (PLZF)-positive cells per tubules compared to controls (6.8 ± 2.2 versus 4.1 ± 1.9), suggesting that the subfertility of *Pole4* mutant is likely due to germ cell attrition (Figure 1E, bottom).

Detailed phenotypic characterization of adult *Pole4*$^{-/-}$ mice revealed skeletal defects and craniofacial abnormalities. While acknowledging the morphological differences between mice and humans, we noted that similar bone dysplasia and facial dysmorphism have also been reported in human patients with mutations of the *POLE1* and *POLE2* genes (Frugoni et al., 2016; Pachlopnik Schmid et al., 2012; Thiffault et al., 2015). In addition to this, we also noticed white patches on the belly, kinks and curls in their tails, and an unusual gait (Figure 1F), which were evident in some, but not all, of the *Pole4*$^{-/-}$ animals (Figure S1F). Micro-computed tomography (CT) scans revealed that the tail kinks or curls in the *Pole4*-deficient mice are caused by vertebrae fusions (Figure 1G),

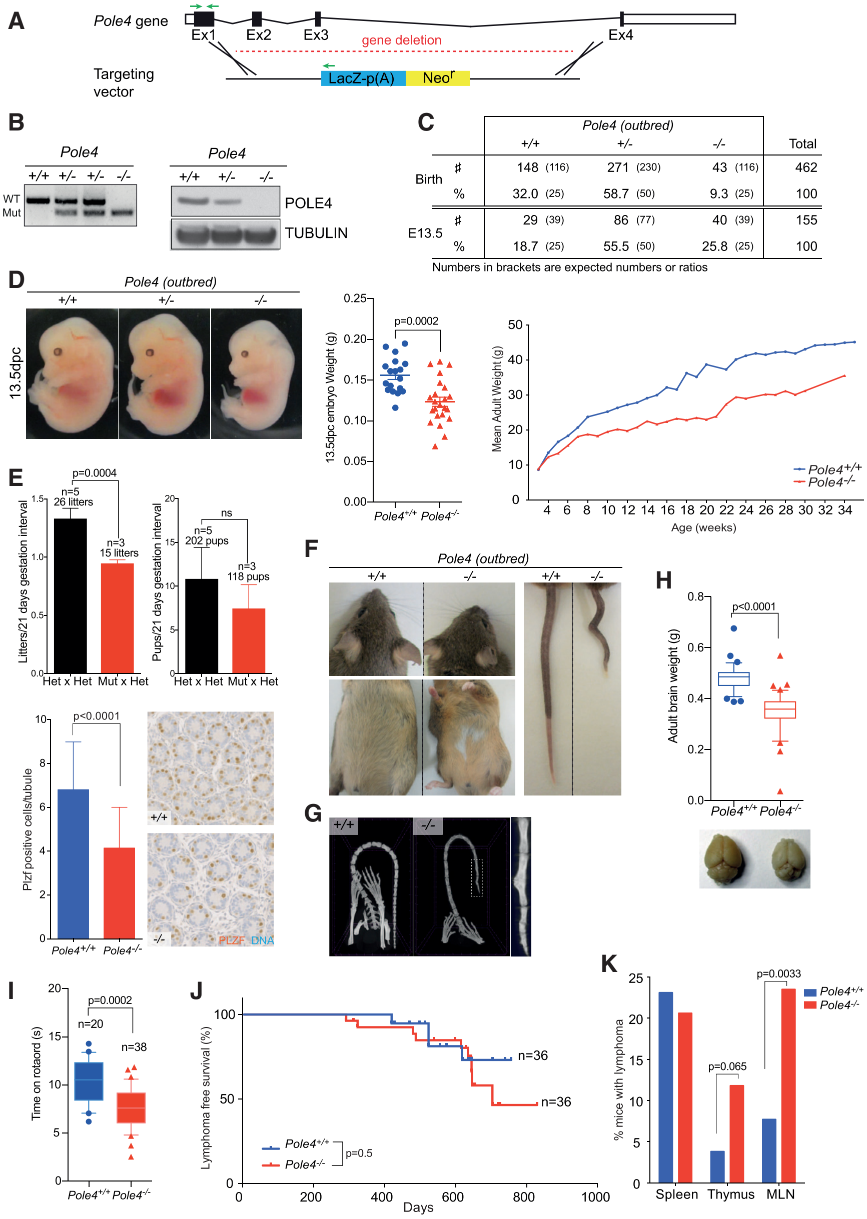

		+/+	+/-	-/-	Total
Birth	#	148 (116)	271 (230)	43 (116)	462
	%	32.0 (25)	58.7 (50)	9.3 (25)	100
E13.5	#	29 (39)	86 (77)	40 (39)	155
	%	18.7 (25)	55.5 (50)	25.8 (25)	100

Numbers in brackets are expected numbers or ratios

Figure 1. Pole4 Knockout Mice Are Viable in an Outbred Strain and Exhibit Phenotypes Similar to Human Patients with Mutations of POLE1 or POLE2

(A) Schematic representation of the targeting vector used to generate the *Pole4*-deficient mice. The deletion cassette has been inserted 93 bp after the start of *Pole4* exon 1 to replace the entire *Pole4* gene. Green arrows represent primers used for genotyping.

(B) Left: *Pole4* genotyping strategy. Upper band represents the WT allele; lower band, the mutant allele. Right: western blot on 13.5 dpc $Pole4^{+/+}$, $Pole4^{+/-}$, $Pole4^{-/-}$ embryos illustrating loss of POLE4. Tubulin was used as loading control.

(C) Breeding summary of $Pole4^{+/-}$ mice intercrosses at birth and during embryogenesis. Numbers and percentages in brackets are expected numbers and ratios. *Pole4*-deficient mice are represented at expected ratios at 13.5 dpc, whereas only 9.3% $Pole4^{-/-}$ mice are born.

(D) Left: representative images of 13.5 dpc *Pole4* embryos. Note the smaller size of $Pole4^{-/-}$ embryo. Scale bar, 1 mm. Middle: weight analysis of *Pole4* embryos at 13.5 dpc. Error bars represent ± SEM of n = 18 $Pole4^{+/+}$ and n = 23 $Pole4^{-/-}$. Significance: t test, p = 0.0002. Right: weight analysis of $Pole4^{+/+}$ and $Pole4^{-/-}$mice. Error bars are not shown to render the graph readable; data are from males and females with at least five mice measured at each time point.

(E) Top: breeding ability of *Pole4* heterozygous × heterozygous versus mutant × heterozygous crosses. Each pair was bred during 5 months, and the number of litters and pups per litter was quantified per 21-day gestation. Significance: t test; litter per 21-day gestation, p = 0.0004; pups per 21-day gestation, p = 0.21. Bottom: testis tubules of 5-day-old neonates were stained with PLZF (germ cells; brown) marker. DNA was counterstained with hematoxylin (blue). Spermatogonia cell quantification per tubule. Error bars represent ± SEM of at least 50 tubules. Significance: t test, p < 0.0001.

(F) Representative pictures of 5-month-old $Pole4^{+/+}$ and $Pole4^{-/-}$ littermates illustrating craniofacial abnormalities, belly white patches, and kinks in the tail.

(G) Micro-CT scan of tail from $Pole4^{+/+}$ and $Pole4^{-/-}$ mice. Bar, 2.5 mm.

(H) Brain-weight analysis of $Pole4^{+/+}$ and $Pole4^{-/-}$ mice. Error bars represent ± SEM of n = 34 $Pole4^{+/+}$ and n = 41 $Pole4^{-/-}$. Significance: t test, p < 0.0001.

(I) Rotarod experiment testing 3-month-old $Pole4^{+/+}$ and $Pole4^{-/-}$ mice coordination. Note the decreased time spent on the rotating rod by $Pole4^{-/-}$ mice compared to their WT littermates. Error bars represent ± SEM of n = 20 $Pole4^{+/+}$ and n = 38 $Pole4^{-/-}$. Significance: t test, p = 0.0002.

(J) Lymphoma-free survival of *Pole4* mice. Significance: Mantel-Cox test, p = 0.5. n = 36 $Pole4^{+/+}$ and n = 36 $Pole4^{-/-}$. Mice culled due to nonspecific phenotypes (e.g., dermatitis, overgrown teeth, and fits) were excluded from this study.

(K) Frequency of *Pole4* mice with lymphomas in spleen, thymus, and mesenteric lymph nodes (MLNs). Significance: Fisher's exact test, p = 0.86 for spleen, p = 0.0652 for thymus, and p = 0.0033 for MLNs.

suggestive of a notochord formation defect (Farkas and Chapman, 2009).

$Pole4^{-/-}$ mice had reduced brain weight, albeit with a relative normal ratio between whole body and brain size (Figure 1H; $Pole4^{+/+}$, 0.48 ± 0.056 g versus $Pole4^{-/-}$, 0.35 ± 0.086 g). We also investigated the unusual gait of these mice by monitoring their ability to stay upright on a rotating rod. *Pole4* mutant mice fell around 3 s earlier from the rod than their WT littermates (Figure 1I), suggesting the *Pole4* deficiency leads to loss of motor function/coordination in mice. Importantly, the latter did not worsen with age, suggesting that this ataxic phenotype is developmental in origin and not degenerative (Figure S1G). In line with a developmental defect, cerebellum sectioning of 3-month-old mutant mice showed a reduced cerebellum size and a severe

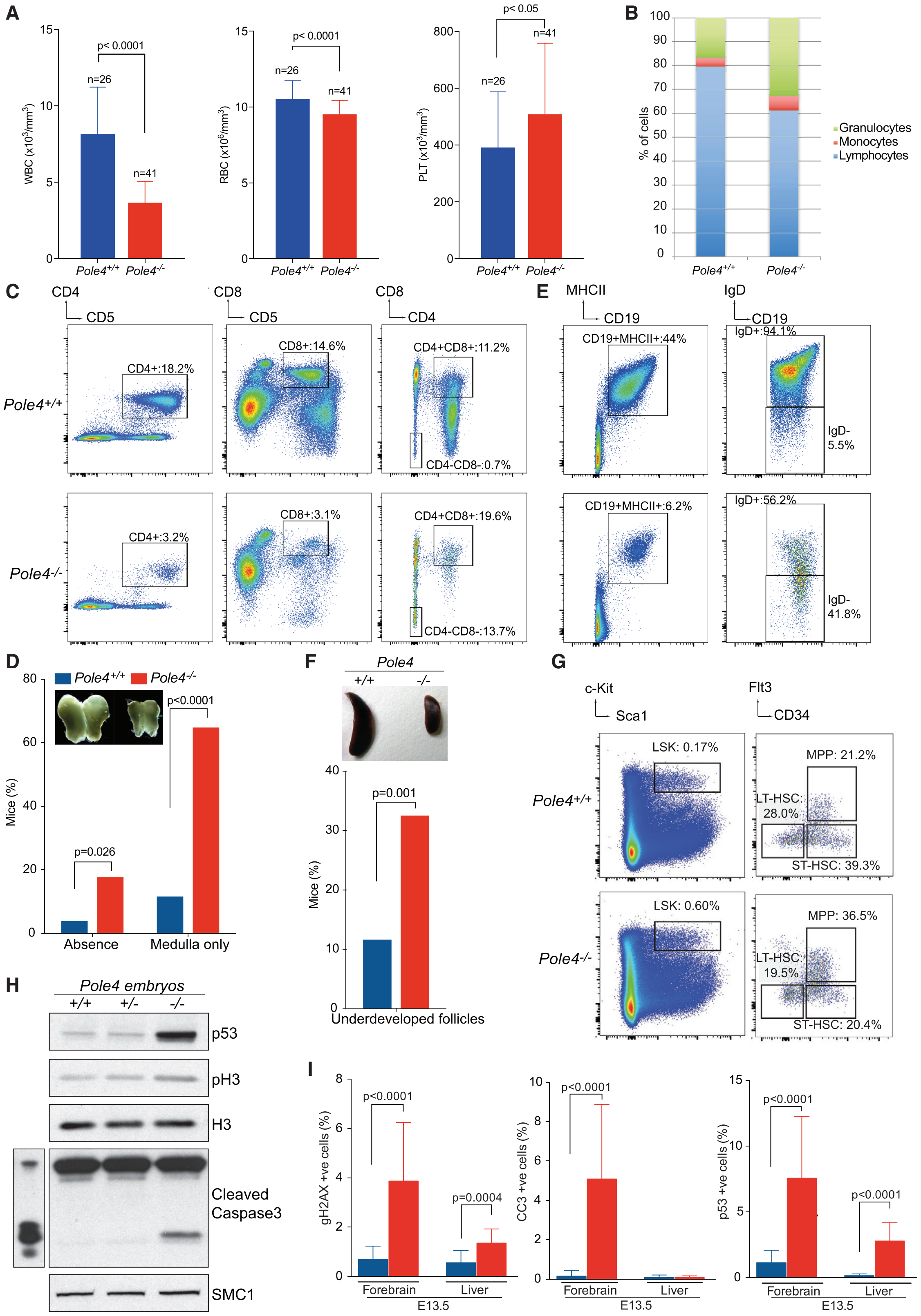

(legend on next page)

foliation defect. Whereas WT mice presented with 7–10 lobules, only 3–6 lobules were detectable in the cerebellum of *Pole4*$^{-/-}$ mice, suggesting that cerebellar development is impaired after loss of POLE4 (Figure S1H). This is further supported by the lengthening of the roof plate in embryos at 13.5 dpc, which results in defective midline fusion (Figure S1I).

To determine the long-term effect of POLE4 loss *in vivo*, we conducted an aging study on 36 WT and 36 Pole4$^{-/-}$ mice. Pole4 deficiency did not lead to a reduction in lifespan (Figure 1J) but did result in increased incidence of lymphomas in the thymus (around 12% of Pole4-deficient mice compared to 4% WT mice) and mesenteric lymph nodes (23.5% in Pole4$^{-/-}$ versus 7.7% in WT; Figure 1K).

Loss of POLE4 Leads to Failed Lymphoid Progenitor Maturation and p53 Activation

Analysis of hematological parameters showed that *Pole4*-deficient mice exhibit leukopenia, with a 2.3-fold decrease in the number of white blood cells, moderate anemia, and thrombocytosis (Figure 2A). We subsequently discovered that leukopenia is caused by a lymphopenia associated with an increase in the proportion of granulocytes and monocytes (Figure 2B). To identify which subset of lymphocytes is affected, we performed flow cytometry analysis of *Pole4* mice splenocytes. As shown in Figure 2C, *Pole4* mutant mice showed a 5-fold decrease of CD4^{+} and CD8^{+} T lymphocytes and a 20-fold increase in the number of CD4 CD8 double-negative precursor cells, compared to WT controls. As maturation of the T cell precursors occurs in the thymus, we examined histological thymus sections from WT and *Pole4*$^{-/-}$ mice (Figure S2A). We discovered that *Pole4*$^{-/-}$ thymus are devoid of cortex and are composed of medulla only structures, suggesting that T cell maturation is severely compromised upon POLE4 loss (Figure S2A). Indeed, around 80% of *Pole4*-deficient mice have absent or a structurally disorganized thymus (Figure 2D).

Pole4 mutant mice also presented with an 8-fold reduction in the number of B cells, which tended to stay at an immature IgD-negative stage (Figure 2E). Mutant mice have a smaller spleen than WT animals and a smaller spleen weight compared to body weight (Figures 2F and S2B). Histological examination of *Pole4*$^{-/-}$ spleens revealed that more than 30% of mutant mice have spleens with inconspicuous and/or underdeveloped follicles, suggestive of impaired B cell maturation. In addition, mesenteric lymph nodes showed a disorganized structure with underdeveloped follicles (Figure S2C).

As lymphoid organs are originally seeded by hematopoietic stem cells (HSCs), we analyzed the HSC compartment in the bone marrow of *Pole4*-deficient mice. Contrary to expectation, loss of POLE4 led to a 3.5-fold increase in the number of HSCs compared to WT littermates as seen by the increase in the number of LSK (Lin^{-}Sca1^{+}cKit^{+}) cells in the bone marrow (Figure 2G, left panel). Long-term (LT-HSC, CD34^{-}Flt3^{-}) and short-term (ST-HSC, CD34^{+}Flt3^{-}) HSCs were reduced in mutant mice, whereas the number of multipotent progenitors (MPPs, CD34^{+} Flt3^{+}) was increased by around 2-fold (Figure 2G, right panel). By performing cobblestone area forming assays to assess the differentiation abilities of HSCs, we noticed that *Pole4*-deficient HSCs retain the ability to differentiate properly to common myeloid progenitors, notably into granulocytes and monocytes progenitors (Figure S2D), which is in accordance with the increase in the number of granulocytes and monocytes previously observed (Figure 2B). These results suggest that *Pole4*-deficient HSCs can differentiate into downstream progenitors, but long-term HSCs may either fail to self-renew to maintain adequate HSC numbers or die due to an increased apoptosis.

To distinguish between these possibilities, we assessed the levels of apoptosis and DNA damage during embryogenesis, particularly in the liver, which is one of the main hematopoietic organs during development. *Pole4*-deficient embryos (13.5 dpc) presented elevated p53 expression levels and increased cleaved caspase 3 (CC3) (Figure 2H). Immunohistochemistry experiments on 13.5 dpc embryos showed that apoptosis (p53 and CC3 staining) and DNA damage (as shown by γH2AX staining) are increased in both forebrain and liver (Figures 2I and S2E) in the *Pole4* mutant, suggesting that HSCs likely die as a consequence of p53 activation and apoptosis. These data also suggest that the reduced growth and developmental alterations (such as dwarf-like craniofacial abnormalities and

Figure 2. Loss of POLE4 Leads to Failed Lymphoid Progenitor Maturation following p53 Activation

(A) White blood cell (left), red blood cell (middle), and platelet (right) counts in *Pole4*$^{+/+}$ and *Pole4*$^{-/-}$ mice. n = 26 *Pole4*$^{+/+}$ and n = 41 *Pole4*$^{-/-}$. Significance: t test, $p < 0.0001$ for white blood cells (WBCs) and red blood cells (RBCs), $p < 0.05$ for platelets.

(B) White blood cell distribution. Note the lower number of lymphocytes in *Pole4*$^{-/-}$ mice.

(C) Representative flow cytometry plots of *Pole4*$^{+/+}$ and *Pole4*$^{-/-}$ mice spleen gated on T lymphocytes and demonstrating a significant reduction of CD4^{+} and CD8^{+} single-positive cells and an increase in CD4^{-}CD8^{-} double-negative population. At least three animals have been analyzed per condition, and analyses were done in triplicates. CD5 was used as T cell marker

(D) Frequency of *Pole4*$^{+/+}$ and *Pole4*$^{-/-}$ mice presenting abnormal thymus. Note the size of *Pole4*$^{-/-}$ thymus is greatly reduced compared to WT littermates. Significance: Fisher's exact test, p = 0.026 for thymus absence, $p < 0.0001$ for thymus with medulla only. n = 36 *Pole4*$^{+/+}$ and n = 36 *Pole4*$^{-/-}$.

(E) Representative flow cytometry plots of *Pole4*$^{+/+}$ and *Pole4*$^{-/-}$ mice spleen gated on B lymphocytes and demonstrating a significant reduction of CD19^{+} cells and an increase in IgD^{-} population. At least 3 animals have been analyzed per condition, and analyses were done in triplicates.

(F) Top: representative picture of spleen from *Pole4* mice. Note the reduction in size following deletion of *Pole4*. Bottom: frequency of *Pole4*$^{+/+}$ and *Pole4*$^{-/-}$ mice presenting spleen with underdeveloped follicles. n = 36 *Pole4*$^{+/+}$ and n = 36 *Pole4*$^{-/-}$. Significance: Fisher's exact test, p = 0.001.

(G) Left: representative flow cytometry plots of *Pole4*$^{+/+}$ and *Pole4*$^{-/-}$ mice bone marrow gated on Lin-Sca1^{+}cKit^{+} hematopoietic stem cells (HSCs) and demonstrating an increase of HSCs in *Pole4*$^{-/-}$ mice. Right: *Pole4*$^{+/+}$ and *Pole4*$^{-/-}$ mice bone marrow gated on CD34 and Flt3 illustrating a decrease of long-term and short-term HSCs and an increase of multipotent progenitors in *Pole4*$^{-/-}$ mice. At least three animals have been analyzed per conditions, and analyses were done in triplicates.

(H) Western blot analysis of proliferation and apoptosis in *Pole4*$^{+/+}$ and *Pole4*$^{-/-}$ embryos at 13.5 dpc. Note the increased expression of p53 and caspase 3 cleavage in *Pole4*$^{-/-}$ extracts. SMC1 was used as loading control.

(I) Immunohistochemistry quantification of γH2AX, cleaved caspase 3, and p53 in forebrain, and liver section of 13.5 dpc embryos. Significance: t test.

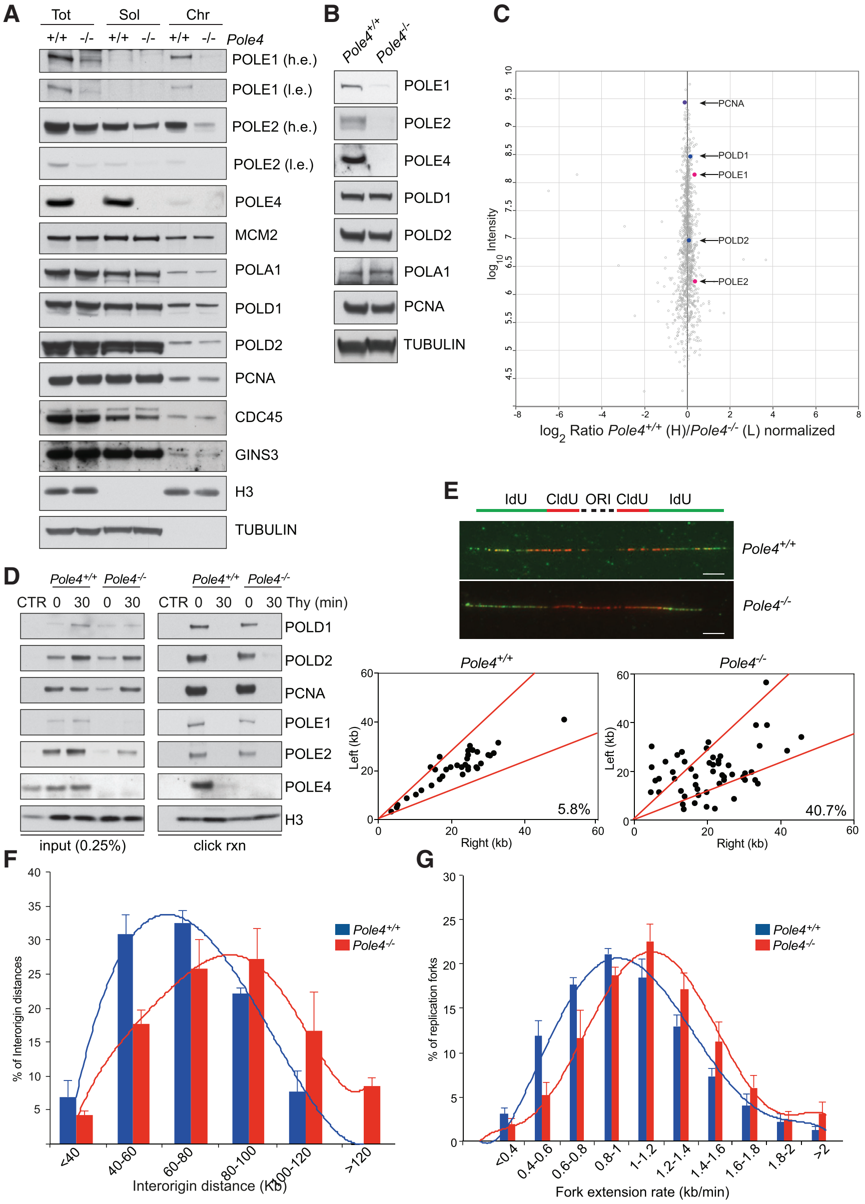

Figure 3. *Pole4*$^{-/-}$ Mouse Cells Exhibit Polε Complex Instability and Heightened Replication Stress

(A) Western blot analysis of replication proteins from total, soluble, and chromatin fractions of *Pole4*$^{+/+}$ and *Pole4*$^{-/-}$ MEFs. Tubulin and histone H3 were used as loading controls.

(B) Western blot analysis of replication proteins from *Pole4*$^{+/+}$ and *Pole4*$^{-/-}$ mice testis extracts. Tubulin was used for normalization.

(C) Results of the iPOND-SILAC-MS experiment reported as logarithmic fold change of heavy/light ratio. Polδ and Polε major subunits are indicated in the plot as blue and red dots, respectively.

(D) *Pole4*$^{+/+}$ and *Pole4*$^{-/-}$ cells were labeled with EdU for 10 min and subjected or not to 30-min chase in media containing thymidine before being processed for iPOND. Captured proteins were analyzed by SDS-PAGE and western blot using the indicated antibodies.

(E) Analysis of fork symmetry in *Pole4*$^{+/+}$ and *Pole4*$^{-/-}$ MEFs reported as left/right moving fork ratio. Data were obtained from three different *Pole4*$^{+/+}$ and *Pole4*$^{-/-}$ MEF clones; error bars ± SEM are included. Scale bar, 5 μM.

(F) Bar graphs showing inter-origin distance distribution in *Pole4*$^{+/+}$ and *Pole4*$^{-/-}$ cells. Data were obtained from three different *Pole4*$^{+/+}$ and *Pole4*$^{-/-}$ MEF clones; error bars ± SEM are included.

(G) Bar graphs showing replication fork speed distribution in *Pole4*$^{+/+}$ and *Pole4*$^{-/-}$ cells. A total of ~1,000 fiber tracts/condition were analyzed from three different *Pole4*$^{+/+}$ and *Pole4*$^{-/-}$ MEF clones; error bars ± SEM are included.

skeletal abnormalities) observed in *Pole4*$^{-/-}$ embryos and mice may be due to apoptosis-dependent elimination of cells during early stages of embryonic development, similar to that previously described in ATR-Seckel mice (Murga et al., 2009).

DNA Polymerase ε Complex Destabilization Drives Replication Stress in *Pole4*$^{-/-}$ Mouse Embryo Fibroblasts

To investigate the impact of *Pole4* deficiency at a cellular level, we derived mouse embryonic fibroblasts (MEFs) from WT and *Pole4*$^{-/-}$ embryos. *Pole4*-deficient MEFs exhibited reduced proliferative potential when grown under a standard 3T3 protocol and in low-oxygen (5%) conditions (Figure S3A). Furthermore, EdU/DAPI (5-ethynyl-2′-deoxyuridine/4′,6-diamidino-2-phenylindole) flow cytometry and immunofluorescence analyses showed an increased proportion of *Pole4*$^{-/-}$ cells in the G2 phase of the cell cycle as well as increased levels of the replicative stress markers 53BP1 and γH2AX (Figures S3B–S3D). Polε has also been shown to be involved in activation of the intra-S-phase checkpoint in yeast (Lou et al., 2008; Navas et al., 1995). However, we failed to detect a defect in Chk1 or H2AX phosphorylation after HU or UV treatment in cells lacking POLE4 (Figure S3E), suggesting that the checkpoint response remains intact.

To identify the possible source of replicative stress in *Pole4*$^{-/-}$ cells, we first analyzed whole-cell extracts by immunoblotting for the levels of Polε, Polδ, Polα, and other components of the replisome (Figure 3A). In contrast to controls, the levels of the major catalytic subunit of Polε, POLE1, were strongly decreased, corresponding to less than 5%–10% of WT levels. We also noticed a reduction, albeit to a lower level, of the second major subunit, POLE2 (Figure 3A). This finding differentiates the mammalian Polε complex from its yeast ancestor, in which the smallest subunits, Dpb3 and Dpb4, are dispensable for maintaining Pol2/Dpb2 stability. This is also reminiscent of *Pold3* deletion in mice, which severely affects the stability of the whole Polδ complex, resulting in early embryonic lethality (Murga et al., 2016).

In whole-cell extracts, loss of POLE4 did not significantly affect the levels of POLD1, POLD2, POLA1 (component of the Polα complex), or other major components of the replication machinery, such as GINS3, MCM2, and CDC45, which are essential components of the processive CMG helicase (Figure 3A). In addition to this, transient knockdown of POLE4 in human HeLa cells was associated with loss of POLE3 expression and vice versa, which suggests that POLE3 and POLE4 are constitutive partners in mammalian cells and required for each other's stability (Figure S3F). Consistent with our cellular studies, we also observed a strong reduction of both POLE1 and POLE2 protein levels in extracts from testis of *Pole4*$^{-/-}$ mice (Figure 3B); this indicates that the *Pole4* knockout is a Polε hypomorphic mouse model, which may explain the subfertility observed in *Pole4*$^{-/-}$ mice (Figure 1). Of note, while *Pole4*$^{+/-}$ cells showed an $\sim$50% reduction of POLE4 protein compared to WT, levels of POLE1 were not significantly changed, potentially explaining the milder phenotype observed in *Pole4* heterozygous mice, embryos, and cells (Figures S3G–S3I).

To investigate the levels and stoichiometry of protein complexes actively involved in DNA replication, we proceeded to analyze the levels of replication factors on chromatin in WT and *Pole4*-deficient MEFs (Figure 3A, chromatin panel). Consistent with Polε complex hypomorphy, chromatin levels of POLE1 were severely decreased in the knockout cells; this was associated with a strong reduction in chromatin-bound POLE2 (compared to total extracts), which points to reduced levels of the whole Polε complex on chromatin. Other replication factors, such as PCNA and Polδ subunits POLD1 and POLD2, as well as CDC45 and GINS3, were largely unaffected in the knockout cells (Figure 3A, chromatin panel).

To distinguish between chromatin-bound proteins and proteins actively traveling with the replication fork, we employed SILAC (stable isotope labeling with amino acids in cell culture)-based proteomics coupled to iPOND (isolation of protein on nascent DNA strands) to quantitatively analyze the composition of replisomes from WT and *Pole4*$^{-/-}$ MEFs (Dungrawala et al., 2015; Lopez-Contreras et al., 2013; Sirbu et al., 2011). To this end, we EdU-pulse labeled WT and *Pole4*$^{-/-}$ cells, grown in heavy and light media, respectively, and subjected them to iPOND and mass spectrometry analysis (Figure S4A). Despite the strong reduction in total and chromatin-bound levels of Polε subunits, we did not detect a major imbalance of polymerases at the replication fork, or the accumulation of other accessory TLS polymerases (Figure 3C; data not shown). Similar results were obtained from the reverse experiment in which WT and *Pole4*$^{-/-}$ cells were grown in light and heavy media, respectively (Figures S4B and S4C). Consistent with our SILAC-based quantitative measurements, iPOND western blot experiments showed no significant differences in POLE1 and POLE2 protein levels at active replication forks between *Pole4*-proficient and -deficient cells (Figure 3D). These data suggest that, once replication forks are established in *Pole4*$^{-/-}$ cells, a Polε complex composed of POLE1 and POLE2 remains stably associated with the replisome and that a POLE1/2 subcomplex is sufficient to initiate processive DNA replication.

A possible explanation for the reduced levels and altered stoichiometry of chromatin-bound Polε complex in *Pole4*$^{-/-}$ MEFs is that CDC45 and the GINS complex are recruited to DNA replication origins, but a functional CMG is not efficiently established, as recently showed by *in vivo* auxin-degron studies in *S. cerevisiae* (Miyazawa-Onami et al., 2017). Consistent with this hypothesis, *Pole4*$^{-/-}$ cells obtained from inbred C57BL/6 embryos showed significant reduction in the levels of PCNA and POLD1 with minimally affected levels of CDC45 and GINS proteins (Figure S4D). Furthermore, transient knockdown of POLE1 in human HeLa cells did not affect chromatin levels of CDC45 or GINS complex component GINS1 (Figure S4E). These data suggest that Polε hypomorphy might impair formation and/or activation of a stable CMG complex, resulting in reduced DNA replication origin activation. Alternatively, reduced processivity, as observed in the yeast Pol2-Dpb2 subcomplex, lacking Dpb3 and Dpb4 (Aksenova et al., 2010), could be the primary cause of replication stress and DNA damage accumulation observed in *Pole4*-deficient cells.

To study replication fork activation and progression at a single-molecule level, we incubated WT and *Pole4*$^{-/-}$ cells with 5-chloro-2'-deoxyuridine (CldU) and then 5-iodo-2'-deoxyuridine (IdU) and monitored replication fork dynamics by fiber stretching assays (Bellelli et al., 2014; Figures 3E–3G). This analysis revealed a significant increase in the frequency of asymmetric forks in *Pole4*$^{-/-}$ cells when compared to WT controls (Figure 3E). Fork stalling events in response to replication stress are associated with dormant origin activation and a corresponding reduction of both IOD (inter-origin distance) and fork speed in mammalian cells, most likely due to compensation mechanisms based on titration of replication factors and deoxyribonucleoside triphosphate (dNTP) levels (for review, see Técher et al., 2017). Fiber stretching analysis revealed a modest increase in both IOD and fork speed distribution in *Pole4*$^{-/-}$ MEFs compared to their WT counterparts (Figures 3F and 3G). The discrepancy between IOD, fork speed, and fork asymmetry observed in *Pole4*$^{-/-}$ cells is reminiscent of that previously described in mouse cells harboring a Chaos3 allele of *Mcm4* (Shima et al., 2007), which exhibits fork asymmetry in the absence of reduced IOD due to extensive reduction of chromatin levels of MCM2–7 hexamers and reduced origin activation (Kawabata et al., 2011a).

In contrast to *Mcm4*$^{(Chaos3)}$, Polε hypomorphy was not associated with reduced levels of chromatin-bound MCM2–7 (Figure 3A). Thus, we reasoned that Polε instability might lead to reduced stable CMG formation/activation and, as a consequence, to reduced or altered spatiotemporal activation of DNA replication origins. Consistent with this notion, we detected a significant reduction in the total number of initiation events in *Pole4*$^{-/-}$ cells compared to WT (Figure S4F). Furthermore, *Pole4*$^{-/-}$ MEFs generated from the more severely affected C57BL/6 inbred background exhibited significantly higher replication fork extension rates when compared to their WT counterparts (Figure S4G). It was previously shown that IOD and fork speed distributions vary significantly between different mouse strains; for instance, MEFs from a C57BL/6 strain show reduced origin activation and a compensatory higher fork speed compared to cells from outbred genetic backgrounds (Kawabata et al., 2011b). We speculate that this phenomenon might explain the embryonic lethality of the *Pole4* knockout allele in the inbred C57BL/6 background (Figures 1 and S1). Collectively, these data

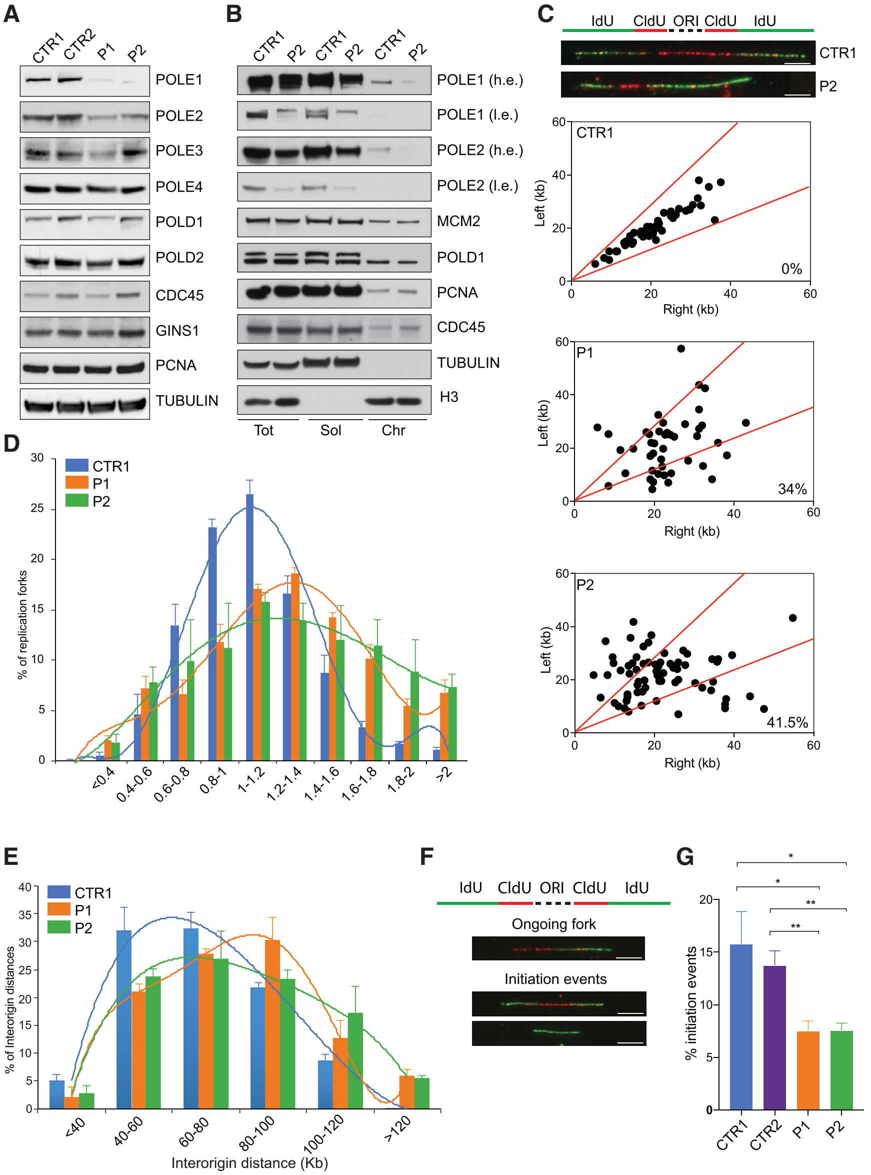

Figure 4. Human *POLE1* Patient Cells Exhibit Polε Instability and Replication Stress

(A) Western blot analysis of Polε subunits and replisome components from total extracts of control- (CTR1 and CTR2) and patient-derived (P1 and P2) cell lines. Tubulin was used for normalization.

(B) Western blot analysis of total, soluble, and chromatin fractions from P2 mutant and CTR1 cells. Tubulin and histone H3 were used as loading controls.

(C) Analysis of fork symmetry in *POLE1* mutant (P1 and P2) and CTR1 cells reported as left/right moving fork ratio. In the top panel, a scheme of the labeling strategy is presented, together with representative pictures of symmetric and asymmetric DNA fibers from CTR1 and P2 cells, respectively. Scale bar, 5 μM.

(D) Bar graphs showing replication fork speed distribution in *POLE1* mutant and control cells. A total of ~1,000 fiber tracts/condition were analyzed from triplicate experiments; error bars ± SEM are included.

(E) Bar graphs showing inter-origin distance distribution in *POLE1* mutant and control cells. Data plotted were obtained from triplicate experiments; error bars ± SEM are included.

(F) Representative scheme of the nucleotide labeling strategy together with representative pictures of the replication structures (ongoing forks and initiation events) analyzed. Scale bar, 5 μM.

(G) Bar graph showing the percentage of initiation events in *POLE1* mutant and control cells. Data were obtained from triplicate experiments (*p < 0.05; **p < 0.01); error bars ± SEM are included.

suggest that the POLE3-POLE4 subcomplex, while playing an important role in maintaining Polε complex stability, is not required for maximal replication rate extension *in vivo*. Furthermore, our data suggest that loss of POLE4 leads to reduced and/or imbalanced origin firing potentially due to defective CMG complex formation and/or activation.

Polε Complex Instability and Replication Stress in *POLE1* Patient-Derived Cells

A number of human Mendelian disorders are caused by mutations in essential genes involved in distinct aspects of DNA replication (for review, see Jackson et al., 2014). Whole-genome sequencing of two patients with microcephalic dwarfism, intra-uterine growth retardation, immunodeficiency, and endocrine insufficiencies identified compound heterozygous mutations in *POLE1* for the same intronic variant, c.168^{+}32C > G, and a further mutation predicted to result in loss of POLE1 functional transcripts (C.L., J.E. Murray et al., unpublished data; see clinical synopsis in STAR Methods). Since intra-uterine growth retardation and marked reduction in postnatal size and lymphopenia are also seen in the *Pole4* mouse model (Figures 1 and 2), we hypothesized that this model would help in understanding the human disease pathogenesis. We therefore investigated whether *POLE1* patient cells exhibit the same molecular defects as those observed in *Pole4*$^{-/-}$ mouse cells.

To analyze the impact of *POLE1* mutations on Polε subunits levels, DNA replication, and genome stability, primary dermal fibroblast lines were obtained from the newly identified patients (P1 and P2). Like *Pole4*$^{-/-}$ cells, *POLE1* patient-derived cell lines showed reduced proliferative potential in culture and displayed elevated levels of DNA damage, as measured by 53BP1 and γH2AX foci accumulation under non-challenging conditions (Figures S5A–S5C). Immunoblotting revealed that both P1 and P2 patient-derived cell lines showed a strong decrease in the levels of POLE1, and a significant reduction in the levels of POLE2, but not POLE3 and POLE4 (Figures 4A and 4B). Other replisome components were unaffected in both cell lines either in total or

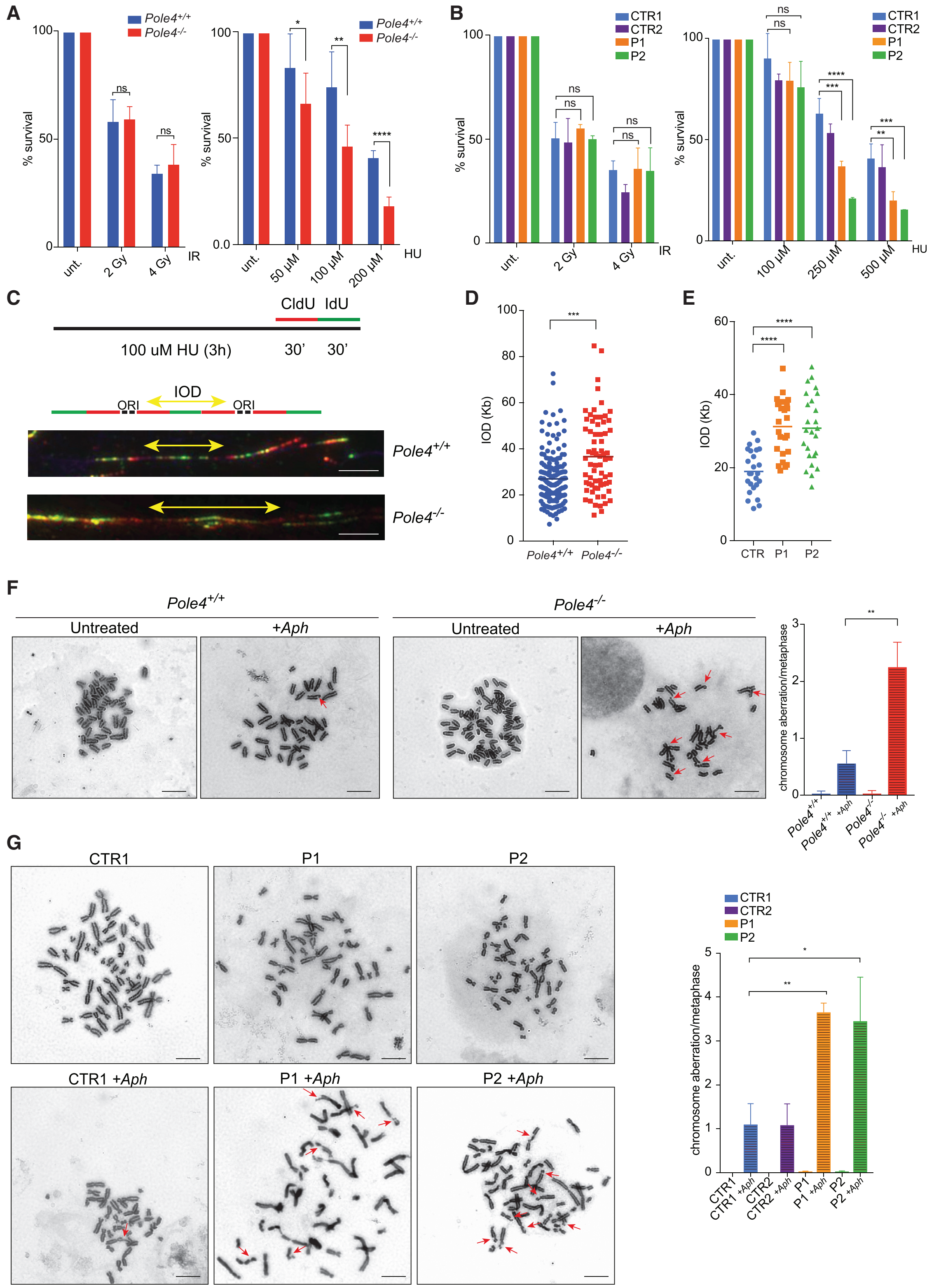

Figure 5. Defective Origin Activation Leads to Chromosomal Instability in *Pole4*$^{-/-}$ and *POLE1* Patient Cells

(A) *Pole4*$^{-/-}$ and *Pole4*$^{+/+}$ cells were subjected to IR or HU (hydroxyurea) treatment, with the described doses, and cell viability was assessed after 5 days (ns, not significant; *p < 0.05; **p < 0.01; ****p < 0.0001); error bars ± SD are included.

(legend continued on next page)

in chromatin extracts. Notably, we observed a stronger reduction in POLE1 levels, compared to that previously reported in *POLE1* patient-derived lymphoblastoid cell lines (Pachlopnik Schmid et al., 2012), which may explain the more severe growth failure observed in P1 and P2 heights.

Analysis of replication fork parameters revealed that *POLE1* patient cells showed a significant increase in asymmetric forks (Figure 4C) and an accumulation of longer, newly incorporated nucleotide tracts (Figure 4D). IOD distribution was also significantly different between non-affected and affected patient cells, with the latter presenting with significant increased IOD (Figure 4E). Consistent with this, the overall percentage of initiation events was significantly reduced in the affected patient cells (Figures 4F and 4G). These data reveal striking similarities between *Pole4*-deficient MEFs and human *POLE1* mutant cells and suggest a common pathogenic mechanism, potentially involving impaired replication fork establishment.

Reduced Origin Activation Drives Chromosomal Instability in Polε Hypomorphic Mouse and Human Cells

We hypothesized that if Polε hypomorphic mouse and human patient cells are compromised for origin activation, they should exhibit hydroxyurea (HU) and/or aphidicolin (Aph) sensitivity, similar to *Mcm4*$^{(Chaos3)}$ mice and patients cells with an MCM4-NT truncation (Gineau et al., 2012; Shima et al., 2007). Consistent with this hypothesis, *Pole4*$^{-/-}$ cells and *POLE1* patient cells were not sensitive to ionizing radiation (IR) but showed heightened sensitivity to HU compared to their WT counterparts (Figures 5A and 5B).

We reasoned that Polε hypomorphic cells might show a reduced ability to activate DNA replication origins in conditions of replication stress, such as HU treatment, and would not exhibit significant IOD reduction (Ge et al., 2007; Kubota et al., 2011). Indeed, *Pole4*$^{+/+}$ MEFs and human control cells showed a very low mean IOD upon HU treatment, consistent with activation of dormant origins. On the contrary, both *Pole4*$^{-/-}$ and *POLE1* patient cells showed higher IOD values when challenged with HU, which is consistent with inefficient origin activation (Figures 5C–5E).

To analyze the consequences of reduced origin activation upon replication stress, we exposed WT and *Pole4*$^{-/-}$ cells, as well as control and patient cells, to low Aph doses for 24 hr and analyzed chromosome spreads by Giemsa staining. As shown in Figures 5F and 5G, under unchallenged conditions we failed to detect signs of chromosomal instability in *Pole4*$^{-/-}$ and *POLE1* mutant cells. However, treatment with low Aph dose resulted in a dramatic increase in the frequency of chromosome breaks and rearrangements in Polε hypomorphic cells (Figures 5F and 5G). Together, these data imply that inefficient origin activation underlies the replication stress response and DNA damage accumulation in Polε hypomorphic mouse and human patient cells.

p53 Deficiency Rescues Embryonic Lethality and Drives Tumorigenesis in *Pole4*$^{-/-}$ Mice

Since *Pole4* null embryos and mice exhibit elevated levels of apoptosis, we proceeded to test the impact of p53 loss on the viability and phenotype of *Pole4*-deficient mice. Strikingly, loss of one or both alleles of p53 was sufficient to rescue the embryonic lethality of *Pole4* mutant mice in the inbred C57BL/6 background (Figure S6A). In contrast to *Pole4*$^{-/-}$ mice, which are essentially embryonic lethal in this inbred background, double-mutant mice were born at submendelian ratios (3.7% born versus 6.25% expected), were devoid of gross abnormalities, and displayed a similar size as WT littermate controls (Figures 6A and S6B). Furthermore, the loss of coordination, observed in rotarod experiments, associated with *Pole4* deficiency was partially rescued by removing both *p53* alleles, but not by a single copy of *p53* (Figure 6B). Loss of both p53 alleles also rescued the absolute number and proportion of white blood cells (Figure 6C). Flow cytometry analysis revealed that the numbers of T cells ($CD4^+$ and $CD8^+$) and B cells in the double mutants were restored to levels comparable to *Pole4*$^{+/+}$ *p53*$^{-/-}$ mice (Figures 6D and 6E). Together, these data establish that p53 loss rescues most of the phenotypes that occur in *Pole4*-deficient mice.

We showed in Figures 1 and S1 that *Pole4* deficiency triggers p53 activation during embryogenesis and is associated with lymphoma development. For this reason, we sought to examine the functional interaction of Pole4 and p53 in lymphoma incidence. To this end, we monitored cohorts of *Pole4*$^{+/+}$ *p53*$^{+/-}$, *Pole4*$^{-/-}$ *p53*$^{-/-}$, *Pole4*$^{+/+}$ *p53*$^{-/-}$, and *Pole4*$^{-/-}$ *p53*$^{-/-}$ mice for ~18 months and performed a Kaplan-Meier analysis to assess lymphoma-free survival (Figure 6F). Strikingly, removal of a single p53 allele in *Pole4*-deficient mice significantly reduced lymphoma-free survival from 480 days in *Pole4*$^{+/+}$ *p53*$^{+/-}$ mice to 226 days in *Pole4*$^{-/-}$ *p53*$^{+/-}$ mice. Furthermore, ~90% of *Pole4*$^{-/-}$ *p53*$^{+/-}$ mice presented with lymphomas (Figure 6G), suggesting that haploinsufficiency for p53 contributes to lymphomagenesis in the absence of POLE4. These data also suggest that *Pole4*$^{-/-}$ mice are specifically prone to lymphoma development since *p53*$^{+/-}$ mice more frequently develop epithelial cancers (Jacks et al., 1994). Collectively, these data establish

(B) *POLE1* mutant (P1 and P2) and control (CTR1 and CTR2) cells were subjected to IR or HU (hydroxyurea) treatment, with the described doses, and cell viability was assessed after 5 days (ns, not significant; **$p < 0.01$; ***$p < 0.001$); error bars ± SD are included.
(C) Upper: representative scheme of the hydroxyurea treatment and CldU-IdU labeling scheme used for fiber stretching assay. Lower: representative images of inter-origin distances (IODs) from *Pole4*$^{+/+}$ and *Pole4*$^{-/-}$ cells. Scale bar, 5 μM.
(D) Mean inter-origin distance values from HU-treated *Pole4*$^{+/+}$ and *Pole4*$^{-/-}$ MEFs (***$p < 0.001$).
(E) Mean inter-origin distance values from HU-treated CTR and *POLE1* mutant cells.
(F) Representative chromosome spreads from *Pole4*$^{+/+}$ or *Pole4*$^{-/-}$ MEFs treated or not with APH (0.3 μm for 24 hr). Yellow arrows indicate scored chromosomal aberrations such as breaks or radials. Scale bar, 10 μM. Right: bar graph showing the main number of chromosomal abnormalities identified in *Pole4*$^{+/+}$ or *Pole4*$^{-/-}$ MEFs treated or not with APH (**$p < 0.01$); error bars ± SD are included.
(G) Representative chromosome spreads from CTR or *POLE1* mutant cells treated or not with APH (0.3 μm for 24 hr). Yellow arrows indicate scored chromosomal aberrations such as breaks or radials. Scale bar, 10 μM. Right: bar graph showing the main number of chromosomal abnormalities identified in the described conditions (*$p < 0.05$; **$p < 0.01$); error bars ± SD are included.

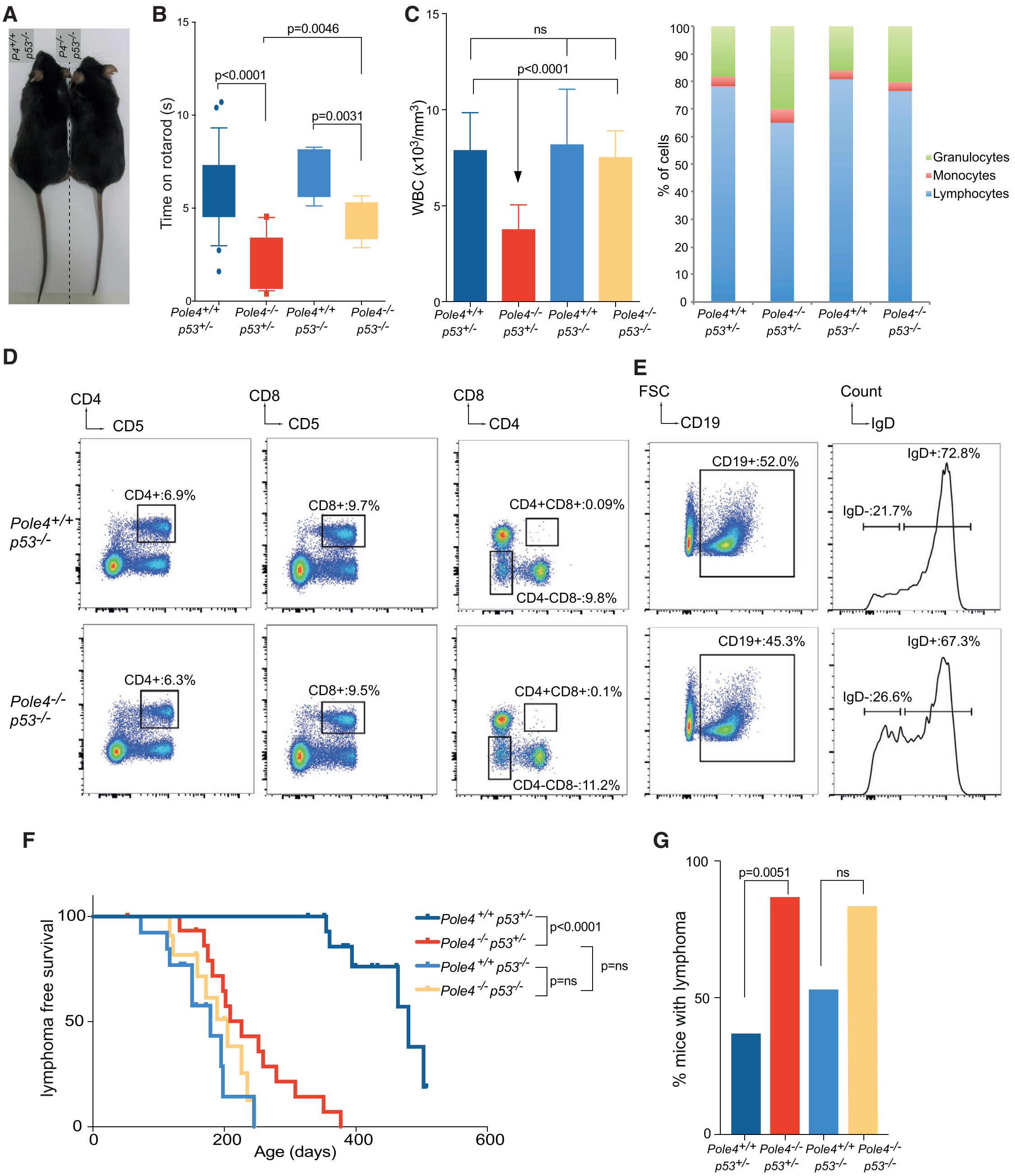

Figure 6. *p53* Deficiency Rescues Embryonic Lethality and Drive Tumorigenesis in *Pole4*$^{-/-}$ Mice

(A) Representative picture of *Pole4/p53* mice. Note the similar size between *Pole4*$^{+/+}$ *p53*$^{-/-}$ and *Pole4*$^{-/-}$ *p53*$^{-/-}$ animals.

(B) Rotarod experiment testing *Pole4*$^{+/+}$ and *Pole4*$^{-/-}$ mice coordination in a *p53*$^{+/-}$ or *p53*$^{-/-}$ background. Note the decreased time spent on the rotating rod by *Pole4*$^{-/-}$*p53*$^{+/-}$ mice compared to their control littermates. Error bars represent ± SEM of n = 27 *Pole4*$^{+/+}$*p53*$^{+/-}$, n = 18 *Pole4*$^{-/-}$*p53*$^{+/-}$, n = 6 *Pole4*$^{+/+}$*p53*$^{-/-}$, n = 6 *Pole4*$^{-/-}$*p53*$^{-/-}$. Significance: t test; *Pole4*$^{+/+}$*p53*$^{+/-}$ versus *Pole4*$^{-/-}$*p53*$^{+/-}$, p < 0.0001; *Pole4*$^{-/-}$*p53*$^{+/-}$ versus *Pole4*$^{+/+}$*p53*$^{-/-}$, p < 0.0001; *Pole4*$^{-/-}$*p53*$^{+/-}$ versus *Pole4*$^{-/-}$*p53*$^{-/-}$, p = 0.0046; *Pole4*$^{+/+}$*p53*$^{+/-}$ versus *Pole4*$^{-/-}$*p53*$^{-/-}$, p = 0.0031; *Pole4*$^{+/+}$*p53*$^{+/-}$ versus *Pole4*$^{+/+}$*p53*$^{+/-}$, p < 0.0001.

(C) Hematology analysis of *Pole4/p53* mice. White blood cell count (left) and distribution (right). n = 24 *Pole4*$^{+/+}$*p53*$^{+/-}$, n = 21 *Pole4*$^{-/-}$*p53*$^{+/-}$, n = 8 *Pole4*$^{+/+}$*p53*$^{-/-}$, n = 11 *Pole4*$^{-/-}$*p53*$^{-/-}$. Significance: t test; *Pole4*$^{+/+}$*p53*$^{+/-}$ and *Pole4*$^{-/-}$*p53*$^{-/-}$ versus *Pole4*$^{-/-}$*p53*$^{+/-}$, p < 0.0001; *Pole4*$^{+/+}$*p53*$^{+/-}$ versus *Pole4*$^{-/-}$*p53*$^{-/-}$, p = 0.633 and *Pole4*$^{+/+}$*p53*$^{+/-}$ versus *Pole4*$^{-/-}$*p53*$^{+/-}$, p = 0.4245. Note the normal distribution of myeloid population in double mutant compared to *Pole4*$^{-/-}$*p53*$^{+/-}$ animals.

(D) Representative flow cytometry plots of *Pole4/p53* mice spleen gated on T lymphocytes and demonstrating a similar number CD4^{+} and CD8^{+} single-positive cells and CD4^{-}CD8^{-} double-negative population. At least three animals have been analyzed per condition, and analyses were done in triplicates. CD5 was used as T cell marker.

(legend continued on next page)

that loss of p53 is able to rescue viability and growth of *Pole4*-deficient mice but increase lymphomagenesis.

DISCUSSION

Polε is a fundamental component of the eukaryotic replisome and mutations of its subunits lead to human genetic disease and cancer (Campbell et al., 2017; Frugoni et al., 2016; Kandoth et al., 2013; Pachlopnik Schmid et al., 2012; Yang et al., 2013). Despite this, the lack of functional and genetic studies on Polε in mammals has limited our understanding of its role in complex organisms. Here, we present the first detailed genetic and mechanistic characterization of mammalian Polε using a mouse knockout and human patient-derived cell lines, which has revealed key roles for Polε during development and in human pathology.

Analysis of embryonic fibroblasts and tissue extracts from *Pole4*$^{-/-}$ mice revealed that POLE4 is required to maintain the stability of the whole Polε complex. Together with the finding that POLD3 is important for Polδ complex stability, our results suggest an important structural role for the accessory subunits of replicative polymerases in mammals (Murga et al., 2016). Despite the strong reduction in total protein levels of Polε, biochemical and proteomic characterization of *Pole4*$^{-/-}$ MEFs showed no significant change in the levels of POLE1 and POLE2 at active replication forks. These data suggest that a POLE1-POLE2 subcomplex is sufficient to initiate and sustain DNA replication in mammalian cells. At the molecular level, *Pole4*$^{-/-}$ MEFs exhibit increased fork rates and larger inter-origin distances associated with accumulation of fork stalling events. This phenotype can be explained by an overall reduction in DNA replication origin activation and excludes defective fork elongation as the initial trigger of replication stress. This also implies that the POLE3–4 subcomplex is dispensable for maintaining normal fork rates in mammalian cells, at least under unchallenged conditions, which differs from what has been observed in *S. cerevisiae* both *in vivo* and *in vitro* (Aksenova et al., 2010). We note that a similar phenotype has been described in MEFs derived from *Mcm4*$^{(Chaos3)}$ mice (Kawabata et al., 2011a; Shima et al., 2007). While levels of chromatin-bound MCMs are unaffected in our model, *Pole4*$^{-/-}$ MEFs showed a similar inability to activate replication origins under conditions of replication stress and a striking sensitivity to replication inhibitors, such as Aph, which results in significant chromosomal instability. We speculate that, in *Pole4*-deficient cells, the levels of Polε are sufficient to sustain normal DNA replication in most tissues and cellular compartments, while the particular dynamics of embryonic replicative cycles and lymphocytes precursor proliferation might affect genome replication and its stability.

Human cell lines derived from patients harboring destabilizing POLE1 mutations showed similar replication profiles, defective origin activation, and severe sensitivity to replication inhibitors to that observed in *Pole4*-deficient MEFs. Similar cellular phenotypes have also been described in cell lines derived from patients affected by mutations of GINS1 and an MCM4-NT truncation (Cottineau et al., 2017; Gineau et al., 2012). We speculate that all these conditions share a common defect in origin activation at the crossroad of CMG helicase formation and/or activation. In our Polε hypomorphic cells, we detected an altered stoichiometry in the chromatin-bound levels of Polε and CMG components, such as GINS subunits and CDC45. This was particularly evident in the C57BL/6 inbred background, which showed greatly reduced levels of PCNA and Polδ subunits on chromatin fraction (suggesting reduced active forks) despite minimal changes in CDC45 and GINS subunits. While CDC45 has been documented to bind to MCMs very early during S-phase and independently from the GINS complex, *in vitro* and *in vivo* data in *S. cerevisiae* have shown that Polε is required for stable CMG formation (Handa et al., 2012; Muramatsu et al., 2010; Sengupta et al., 2013; Yeeles et al., 2015). Here, we establish that chromatin binding of GINS components might not be compromised by reduced Polε levels as recently reported by Miyazawa-Onami et al. using a Pol2 degron strategy (Miyazawa-Onami et al., 2017). While we cannot exclude the possibility of defective stable CMG formation, our data show that Polε is required for initiation of DNA replication in mammalian cells. A role for the budding yeast Polε complex in epigenetic inheritance at the replication fork has been suggested by telomeric silencing defects (Iida and Araki, 2004). Recently, the Dpb3-Dpb4 subcomplex has been directly linked to this phenomenon (He et al., 2017). Future studies will be needed to address a possible role for human Polε and its smallest subunits in histones recycling and/or deposition at the replication fork.

At the organismal level, *Pole4*-deficient mice suffer from intra- and extra-uterine growth retardation, severe developmental abnormalities, lymphopenia, and lymphomagenesis. The growth-retardation phenotype, craniofacial and skeletal abnormalities, and reduction in T and B cell levels closely parallel the clinical phenotypes associated with reported mutations of *POLE1* and *POLE2* in humans, despite the obvious anatomical differences between mice and humans (Frugoni et al., 2016; Pachlopnik Schmid et al., 2012; Thiffault et al., 2015). Similar anomalies with selective immunodeficiencies and growth restriction have also been described in patients with hypomorphic mutations in GINS1 and a N-terminal truncation of MCM4 (not affecting MCM2–7 chromatin levels), which suggests a common pathogenetic mechanism affecting initiation of DNA replication (Cottineau et al., 2017; Gineau et al., 2012; Hughes et al., 2012). Notably, the phenotype for this group of genes appears distinct

(E) Representative flow cytometry plots of *Pole4/p53* mice spleen gated on B lymphocytes and demonstrating a similar number CD19^{+} and IgD^{-} cells. At least three animals have been analyzed per condition, and analyses were done in triplicates.

(F) Lymphoma-free survival of *Pole4/p53* mice. n = 16 *Pole4*$^{+/+}$*p53*$^{+/-}$, n = 16 *Pole4*$^{-/-}$*p53*$^{+/-}$, n = 13 *Pole4*$^{+/+}$*p53*$^{-/-}$, n = 11 *Pole4*$^{-/-}$*p53*$^{-/-}$. Significance: Mantel-Cox test; *Pole4*$^{+/+}$*p53*$^{+/-}$ versus *Pole4*$^{-/-}$*p53*$^{+/-}$, $p < 0.0001$; *Pole4*$^{+/+}$*p53*$^{-/-}$ versus *Pole4*$^{-/-}$*p53*$^{-/-}$, p = 0.3066; *Pole4*$^{-/-}$*p53*$^{+/-}$ versus *Pole4*$^{-/-}$*p53*$^{-/-}$, p = 0.0746. Mice culled due to nonspecific phenotypes (e.g., dermatitis, overgrown teeth, and fits) were excluded from this study.

(G) Lymphoma frequency of *Pole4/p53* mice. Significance: Fisher's exact test; *Pole4*$^{+/+}$*p53*$^{+/-}$ versus *Pole4*$^{-/-}$*p53*$^{+/-}$, p = 0.0051; *Pole4*$^{+/+}$*p53*$^{-/-}$ versus *Pole4*$^{-/-}$*p53*$^{-/-}$, p = 0.1261.

from Meier-Gorlin syndrome (a form of microcephalic dwarfism with microtia and patella hypoplasia/aplasia), which is caused by mutations in genes encoding the pre-replication (PRE-RC) components ORC1,4,6, Cdc6, and Cdt1; Geminin, and the pre-initiation complex (PRE-IC) component CDC45 (Bicknell et al., 2011a; Bicknell et al., 2011b; Fenwick et al., 2016; Guernsey et al., 2011).

Our analysis of *Pole4*-deficient mice suggests that B and T cell development is affected at its initial step, such as the double-negative stage of T cell maturation. Double-negative T cells originate from common precursors migrating from the bone marrow. While having lost self-renewing abilities, double-negative cells undergo a significant proliferative burst in the thymus environment before undergoing successive commitment toward CD4 or CD8 expression (Koch and Radtke, 2011). We suspect that this stage is particularly vulnerable to replication stress due to inefficient origin activation. Consistent with this, previous *POLE1* patients did not show alteration of T cell receptor (TCR) repertoire or immunoglobulin distribution, while switched B cells and memory B cells were equally affected in their total levels, which exclude immunoglobulin G (IgG) and TCR rearrangement defects (Pachlopnik Schmid et al., 2012).

Most of the developmental phenotypes observed in embryos and adult *Pole4*$^{-/-}$ mice likely reflect the consequence of activation of a p53-dependent apoptotic cascade (Figures 1, 2, and 6). Indeed, genetic ablation of p53 in *Pole4*$^{-/-}$ animals is sufficient to rescue the plethora of developmental and immunological defects observed in these mice. These data suggest that embryonic replicative stress is the major driver of the phenotypes described in *Pole4*$^{-/-}$ adult mice and, most likely, in humans suffering hypomorphic mutations of Polε and/or CMG components. Consistent with the important tumor suppressor function of p53, rescue of T and B cell maturation was associated with an increased susceptibility to lymphoma development as observed in *Pole4*$^{-/-}$ *p53*$^{+/-}$ mice.

In conclusion, our analysis of Polε hypomorphy provides a molecular understanding for a new group of genetic conditions at the crossroad of CMG helicase activation. We also provide evidence for an essential role for controlled CMG helicase and origin activation in embryonic development, genome stability, and tumor suppression.

STAR★METHODS

Detailed methods are provided in the online version of this paper and include the following:

- KEY RESOURCES TABLE
- CONTACT FOR REAGENT AND RESOURCE SHARING
- EXPERIMENTAL MODEL AND SUBJECT DETAILS
  - Mouse strains
  - Cell lines
  - Clinical Synopsis of POLE1 patients
- METHOD DETAILS
  - Mice breeding and experiments
  - Histology, immunohistochemistry
  - Alcian blue/Alizarin red staining
  - MicroCT scan
  - Rotarod experiments
  - Cobblestone area forming cell assay (CFAC)
  - Immunophenotyping
  - Western blot analysis of embryos, mouse tissues and cells
  - Mouse Embryonic Fibroblasts (MEFs) isolation and culture
  - Immunofluorescence stainings
  - Chromatin fractionation
  - DNA fiber stretching assay
  - BrdU and EdU FACS
  - Chromosome spreads and GIEMSA staining
  - iPOND (isolation of Proteins on Nascent DNA) and iPOND SILAC Mass Spect
  - POLE4 Antibody generation
  - Analysis of Hydroxyurea and Ionizing radiation sensitivity
- QUANTIFICATION AND STATISTICAL ANALYSIS

SUPPLEMENTAL INFORMATION

Supplemental Information includes six figures and can be found with this article online at https://doi.org/10.1016/j.molcel.2018.04.008.

ACKNOWLEDGMENTS

We would like to thank Penny Jeggo for providing cell lines and the Francis Crick Biological Resource facility for assistance with mouse breeding and maintenance. R.B. and V.B. are funded by the Francis Crick Institute. Boulton lab work is supported by the Francis Crick Institute, which receives its core funding from Cancer Research UK (FC0010048), the UK Medical Research Council (FC0010048), and the Wellcome Trust (FC0010048). S.J.B. is also the recipient of a European Research Council (ERC) Advanced Investigator Grant (TelMetab; Project ID 742437) and Wellcome Trust Senior Investigator (104558/Z/14/Z) and Collaborative Grants. A.J. is supported by funding from an ERC Starter Grant (281847) and by a Medical Research Council Human Genetics Unit core grant (MRC, U127580972).

AUTHOR CONTRIBUTIONS

R.B., V.B., and S.J.B. designed the project and wrote the manuscript with input from A.J. R.B. and V.B. contributed equally to this work. V.B. performed all the work on mice, and R.B. performed experiments on human and mouse cells. J.S. contributed to reagent generation. E.N. and G.S. performed mouse post-mortem analyses and advised on histopathology. D.E.C. assisted with mouse phenotyping. H.R.F. and A.P.S. performed mass spectrometry analysis of the iPOND experiments in *Pole4*$^{+/+}$ and *Pole4*$^{-/-}$ MEFs. F.L. performed the micro-CT scan of the mice. C.L., S.M.O., K.M., and A.J. characterized the human patients, identified the *POLE1* mutations, and provided patient-derived cell lines.

DECLARATION OF INTERESTS

The authors declare no competing interests.

Received: February 5, 2018
Revised: March 28, 2018
Accepted: April 3, 2018
Published: May 10, 2018

REFERENCES

Aksenova, A., Volkov, K., Maceluch, J., Pursell, Z.F., Rogozin, I.B., Kunkel, T.A., Pavlov, Y.I., and Johansson, E. (2010). Mismatch repair-independent

increase in spontaneous mutagenesis in yeast lacking non-essential subunits of DNA polymerase ε. PLoS Genet. *6*, e1001209.

Alexandrov, L.B., Nik-Zainal, S., Wedge, D.C., Aparicio, S.A., Behjati, S., Biankin, A.V., Bignell, G.R., Bolli, N., Borg, A., Børresen-Dale, A.L., et al.; Australian Pancreatic Cancer Genome Initiative; ICGC Breast Cancer Consortium; ICGC MMML-Seq Consortium; ICGC PedBrain (2013). Signatures of mutational processes in human cancer. Nature *500*, 415–421.

Araki, H., Hamatake, R.K., Morrison, A., Johnson, A.L., Johnston, L.H., and Sugino, A. (1991). Cloning DPB3, the gene encoding the third subunit of DNA polymerase II of Saccharomyces cerevisiae. Nucleic Acids Res. *19*, 4867–4872.

Bellelli, R., Castellone, M.D., Guida, T., Limongello, R., Dathan, N.A., Merolla, F., Cirafici, A.M., Affuso, A., Masai, H., Costanzo, V., et al. (2014). NCOA4 transcriptional coactivator inhibits activation of DNA replication origins. Mol. Cell *55*, 123–137.

Bermudez, V.P., Farina, A., Raghavan, V., Tappin, I., and Hurwitz, J. (2011). Studies on human DNA polymerase epsilon and GINS complex and their role in DNA replication. J. Biol. Chem. *286*, 28963–28977.

Bicknell, L.S., Bongers, E.M., Leitch, A., Brown, S., Schoots, J., Harley, M.E., Aftimos, S., Al-Aama, J.Y., Bober, M., Brown, P.A., et al. (2011a). Mutations in the pre-replication complex cause Meier-Gorlin syndrome. Nat. Genet. *43*, 356–359.

Bicknell, L.S., Walker, S., Klingseisen, A., Stiff, T., Leitch, A., Kerzendorfer, C., Martin, C.A., Yeyati, P., Al Sanna, N., Bober, M., et al. (2011b). Mutations in ORC1, encoding the largest subunit of the origin recognition complex, cause microcephalic primordial dwarfism resembling Meier-Gorlin syndrome. Nat. Genet. *43*, 350–355.

Campbell, B.B., Light, N., Fabrizio, D., Zatzman, M., Fuligni, F., de Borja, R., Davidson, S., Edwards, M., Elvin, J.A., Hodel, K.P., et al. (2017). Comprehensive analysis of hypermutation in human cancer. Cell *171*, 1042–1056.

Cottineau, J., Kottemann, M.C., Lach, F.P., Kang, Y.H., Vély, F., Deenick, E.K., Lazarov, T., Gineau, L., Wang, Y., Farina, A., et al. (2017). Inherited GINS1 deficiency underlies growth retardation along with neutropenia and NK cell deficiency. J. Clin. Invest. *127*, 1991–2006.

Donehower, L.A., Harvey, M., Slagle, B.L., McArthur, M.J., Montgomery, C.A., Jr., Butel, J.S., and Bradley, A. (1992). Mice deficient for p53 are developmentally normal but susceptible to spontaneous tumours. Nature *356*, 215–221.

Dungrawala, H., Rose, K.L., Bhat, K.P., Mohni, K.N., Glick, G.G., Couch, F.B., and Cortez, D. (2015). The replication checkpoint prevents two types of fork collapse without regulating replisome stability. Mol. Cell *59*, 998–1010.

Farkas, D.R., and Chapman, D.L. (2009). Kinked tail mutation results in notochord defects in heterozygotes and distal visceral endoderm defects in homozygotes. Dev. Dyn. *238*, 3237–3247.

Fenwick, A.L., Kliszczak, M., Cooper, F., Murray, J., Sanchez-Pulido, L., Twigg, S.R., Goriely, A., McGowan, S.J., Miller, K.A., Taylor, I.B., et al.; WGS500 Consortium (2016). Mutations in CDC45, encoding an essential component of the pre-initiation complex, cause Meier-Gorlin syndrome and craniosynostosis. Am. J. Hum. Genet. *99*, 125–138.

Frugoni, F., Dobbs, K., Felgentreff, K., Aldhekri, H., Al Saud, B.K., Arnaout, R., Ali, A.A., Abhyankar, A., Alroqi, F., Giliani, S., et al. (2016). A novel mutation in the POLE2 gene causing combined immunodeficiency. J. Allergy Clin. Immunol. *137*, 635–638.

Ge, X.Q., Jackson, D.A., and Blow, J.J. (2007). Dormant origins licensed by excess Mcm2-7 are required for human cells to survive replicative stress. Genes Dev. *21*, 3331–3341.

Gineau, L., Cognet, C., Kara, N., Lach, F.P., Dunne, J., Veturi, U., Picard, C., Trouillet, C., Eidenschenk, C., Aoufouchi, S., et al. (2012). Partial MCM4 deficiency in patients with growth retardation, adrenal insufficiency, and natural killer cell deficiency. J. Clin. Invest. *122*, 821–832.

Guernsey, D.L., Matsuoka, M., Jiang, H., Evans, S., Macgillivray, C., Nightingale, M., Perry, S., Ferguson, M., LeBlanc, M., Paquette, J., et al. (2011). Mutations in origin recognition complex gene ORC4 cause Meier-Gorlin syndrome. Nat. Genet. *43*, 360–364.

Handa, T., Kanke, M., Takahashi, T.S., Nakagawa, T., and Masukata, H. (2012). DNA polymerization-independent functions of DNA polymerase epsilon in assembly and progression of the replisome in fission yeast. Mol. Biol. Cell *23*, 3240–3253.

He, H., Li, Y., Dong, Q., Chang, A.Y., Gao, F., Chi, Z., Su, M., Zhang, F., Ban, H., Martienssen, R., et al. (2017). Coordinated regulation of heterochromatin inheritance by Dpb3-Dpb4 complex. Proc. Natl. Acad. Sci. USA *114*, 12524–12529.

Hughes, C.R., Guasti, L., Meimaridou, E., Chuang, C.H., Schimenti, J.C., King, P.J., Costigan, C., Clark, A.J., and Metherell, L.A. (2012). MCM4 mutation causes adrenal failure, short stature, and natural killer cell deficiency in humans. J. Clin. Invest. *122*, 814–820.

Iida, T., and Araki, H. (2004). Noncompetitive counteractions of DNA polymerase epsilon and ISW2/yCHRAC for epigenetic inheritance of telomere position effect in Saccharomyces cerevisiae. Mol. Cell. Biol. *24*, 217–227.

Ilves, I., Petojevic, T., Pesavento, J.J., and Botchan, M.R. (2010). Activation of the MCM2-7 helicase by association with Cdc45 and GINS proteins. Mol. Cell *37*, 247–258.

Jacks, T., Remington, L., Williams, B.O., Schmitt, E.M., Halachmi, S., Bronson, R.T., and Weinberg, R.A. (1994). Tumor spectrum analysis in p53-mutant mice. Curr. Biol. *1*, 1–7.

Jackson, A.P., Laskey, R.A., and Coleman, N. (2014). Replication proteins and human disease. Cold Spring Harb. Perspect. Biol. *6*, 6.

Kandoth, C., McLellan, M.D., Vandin, F., Ye, K., Niu, B., Lu, C., Xie, M., Zhang, Q., McMichael, J.F., Wyczalkowski, M.A., et al. (2013). Mutational landscape and significance across 12 major cancer types. Nature *502*, 333–339.

Kawabata, T., Luebben, S.W., Yamaguchi, S., Ilves, I., Matise, I., Buske, T., Botchan, M.R., and Shima, N. (2011a). Stalled fork rescue via dormant replication origins in unchallenged S phase promotes proper chromosome segregation and tumor suppression. Mol. Cell *41*, 543–553.

Kawabata, T., Yamaguchi, S., Buske, T., Luebben, S.W., Wallace, M., Matise, I., Schimenti, J.C., and Shima, N. (2011b). A reduction of licensed origins reveals strain-specific replication dynamics in mice. Mamm. Genome *22*, 506–517.

Koch, U., and Radtke, F. (2011). Mechanisms of T cell development and transformation. Annu. Rev. Cell Dev. Biol. *27*, 539–562.

Kubota, T., Hiraga, S., Yamada, K., Lamond, A.I., and Donaldson, A.D. (2011). Quantitative proteomic analysis of chromatin reveals that Ctf18 acts in the DNA replication checkpoint. Mol. Cell. Proteomics *10*, M110.005561.

Labib, K. (2010). How do Cdc7 and cyclin-dependent kinases trigger the initiation of chromosome replication in eukaryotic cells? Genes Dev. *24*, 1208–1219.

Lee, S.H., Pan, Z.Q., Kwong, A.D., Burgers, P.M., and Hurwitz, J. (1991). Synthesis of DNA by DNA polymerase epsilon in vitro. J. Biol. Chem. *266*, 22707–22717.

Li, Y., Asahara, H., Patel, V.S., Zhou, S., and Linn, S. (1997). Purification, cDNA cloning, and gene mapping of the small subunit of human DNA polymerase epsilon. J. Biol. Chem. *272*, 32337–32344.

Li, Y., Pursell, Z.F., and Linn, S. (2000). Identification and cloning of two histone fold motif-containing subunits of HeLa DNA polymerase epsilon. J. Biol. Chem. *275*, 23247–23252.

Lopez-Contreras, A.J., Ruppen, I., Nieto-Soler, M., Murga, M., Rodriguez-Acebes, S., Remeseiro, S., Rodrigo-Perez, S., Rojas, A.M., Mendez, J., Muñoz, J., and Fernandez-Capetillo, O. (2013). A proteomic characterization of factors enriched at nascent DNA molecules. Cell Rep. *3*, 1105–1116.

Lou, H., Komata, M., Katou, Y., Guan, Z., Reis, C.C., Budd, M., Shirahige, K., and Campbell, J.L. (2008). Mrc1 and DNA polymerase epsilon function together in linking DNA replication and the S phase checkpoint. Mol. Cell *32*, 106–117.

Masai, H., Matsumoto, S., You, Z., Yoshizawa-Sugata, N., and Oda, M. (2010). Eukaryotic chromosome DNA replication: where, when, and how? Annu. Rev. Biochem. *79*, 89–130.

Méchali, M. (2010). Eukaryotic DNA replication origins: Many choices for appropriate answers. Nat. Rev. Mol. Cell Biol. *11*, 728–738.

Miyazawa-Onami, M., Araki, H., and Tanaka, S. (2017). Pre-initiation complex assembly functions as a molecular switch that splits the Mcm2-7 double hexamer. EMBO Rep. *18*, 1752–1761.

Muramatsu, S., Hirai, K., Tak, Y.S., Kamimura, Y., and Araki, H. (2010). CDK-dependent complex formation between replication proteins Dpb11, Sld2, Pol (epsilon, and GINS in budding yeast. Genes Dev. *24*, 602–612.

Murga, M., Bunting, S., Montaña, M.F., Soria, R., Mulero, F., Cañamero, M., Lee, Y., McKinnon, P.J., Nussenzweig, A., and Fernandez-Capetillo, O. (2009). A mouse model of ATR-Seckel shows embryonic replicative stress and accelerated aging. Nat. Genet. *41*, 891–898.

Murga, M., Lecona, E., Kamileri, I., Díaz, M., Lugli, N., Sotiriou, S.K., Anton, M.E., Méndez, J., Halazonetis, T.D., and Fernandez-Capetillo, O. (2016). POLD3 is haploinsufficient for DNA replication in mice. Mol. Cell *63*, 877–883.

Navas, T.A., Zhou, Z., and Elledge, S.J. (1995). DNA polymerase epsilon links the DNA replication machinery to the S phase checkpoint. Cell *80*, 29–39.

Nick McElhinny, S.A., Gordenin, D.A., Stith, C.M., Burgers, P.M., and Kunkel, T.A. (2008). Division of labor at the eukaryotic replication fork. Mol. Cell *30*, 137–144.

Ohya, T., Maki, S., Kawasaki, Y., and Sugino, A. (2000). Structure and function of the fourth subunit (Dpb4p) of DNA polymerase epsilon in Saccharomyces cerevisiae. Nucleic Acids Res. *28*, 3846–3852.

Pachlopnik Schmid, J., Lemoine, R., Nehme, N., Cormier-Daire, V., Revy, P., Debeurme, F., Debré, M., Nitschke, P., Bole-Feysot, C., Legeai-Mallet, L., et al. (2012). Polymerase ε1 mutation in a human syndrome with facial dysmorphism, immunodeficiency, livedo, and short stature (“FILS syndrome”). J. Exp. Med. *209*, 2323–2330.

Poot, R.A., Dellaire, G., Hülsmann, B.B., Grimaldi, M.A., Corona, D.F., Becker, P.B., Bickmore, W.A., and Varga-Weisz, P.D. (2000). HuCHRAC, a human ISWI chromatin remodelling complex contains hACF1 and two novel histone-fold proteins. . EMBO J. *19*, 3377–3387.

Pursell, Z.F., Isoz, I., Lundström, E.B., Johansson, E., and Kunkel, T.A. (2007). Yeast DNA polymerase epsilon participates in leading-strand DNA replication. Science *317*, 127–130.

Sengupta, S., van Deursen, F., de Piccoli, G., and Labib, K. (2013). Dpb2 integrates the leading-strand DNA polymerase into the eukaryotic replisome. Curr. Biol. *23*, 543–552.

Shima, N., Alcaraz, A., Liachko, I., Buske, T.R., Andrews, C.A., Munroe, R.J., Hartford, S.A., Tye, B.K., and Schimenti, J.C. (2007). A viable allele of Mcm4 causes chromosome instability and mammary adenocarcinomas in mice. Nat. Genet. *39*, 93–98.

Sirbu, B.M., Couch, F.B., Feigerle, J.T., Bhaskara, S., Hiebert, S.W., and Cortez, D. (2011). Analysis of protein dynamics at active, stalled, and collapsed replication forks. Genes Dev. *25*, 1320–1327.

Syvaoja, J., and Linn, S. (1989). Characterization of a large form of DNA polymerase delta from HeLa cells that is insensitive to proliferating cell nuclear antigen. J. Biol. Chem. *264*, 2489–2497.

Tackett, A.J., Dilworth, D.J., Davey, M.J., O’Donnell, M., Aitchison, J.D., Rout, M.P., and Chait, B.T. (2005). Proteomic and genomic characterization of chromatin complexes at a boundary. J. Cell Biol. *169*, 35–47.

Técher, H., Koundrioukoff, S., Nicolas, A., and Debatisse, M. (2017). The impact of replication stress on replication dynamics and DNA damage in vertebrate cells. Nat. Rev. Genet. *18*, 535–550.

Thiffault, I., Saunders, C., Jenkins, J., Raje, N., Canty, K., Sharma, M., Grote, L., Welsh, H.I., Farrow, E., Twist, G., et al. (2015). A patient with polymerase E1 deficiency (POLE1): Clinical features and overlap with DNA breakage/instability syndromes. BMC Med. Genet. *16*, 31.

Tsubota, T., Maki, S., Kubota, H., Sugino, A., and Maki, H. (2003). Double-stranded DNA binding properties of Saccharomyces cerevisiae DNA polymerase epsilon and of the Dpb3p-Dpb4p subassembly. Genes Cells *8*, 873–888.

Yang, Y., Muzny, D.M., Reid, J.G., Bainbridge, M.N., Willis, A., Ward, P.A., Braxton, A., Beuten, J., Xia, F., Niu, Z., et al. (2013). Clinical whole-exome sequencing for the diagnosis of mendelian disorders. N. Engl. J. Med. *369*, 1502–1511.

Yeeles, J.T., Deegan, T.D., Janska, A., Early, A., and Diffley, J.F. (2015). Regulated eukaryotic DNA replication origin firing with purified proteins. Nature *519*, 431–435.

STAR★METHODS

KEY RESOURCES TABLE

REAGENT or RESOURCE	SOURCE	IDENTIFIER
Antibodies		
Goat Anti-Rat IgG (H+L) Antibody, Alexa Fluor594 Conjugated	Thermo Fisher	Cat#A-11007; RRID: AB_141374
Rabbit anti-mouse FITC-conjugated	DAKO	Cat#F0313
Mouse Monoclonal anti-PCNA	Santa Cruz Biotechnology	Cat# sc-56; RRID: AB_628110
Mouse Monoclonal anti-Tubulin	Sigma-Aldrich	Cat#T6074; RRID: AB_477582
Peroxidase-conjugated Goat anti-Mouse IgG (H+L)	Thermo Fisher Scientific	Cat#G-21040; RRID: AB_2536527
Peroxidase-conjugated Goat anti-Rabbit IgG (H+L)	Thermo Fisher Scientific	Cat#G-21234; RRID: AB_2536530
Peroxidase-conjugated Goat anti-Rat IgG (H+L)	N/A	N/A
Rabbit Anti-Mouse IgG (H+L) Antibody, Alexa Fluor488 Conjugated	Thermo Fisher	Cat#A-11059; RRID: AB_142495
Goat Anti-Rabbit IgG (H+L) Antibody, Alexa Fluor488 Conjugated	Thermo Fisher	Cat#A-11034; RRID: AB_2576217
Rabbit polyclonal anti-53BP1	Novus Biologicals	Cat#NB100-304; RRID: AB_10003037
Rat monoclonal anti-Mouse CD5-BV421 clone 53-7.3	BD Biosciences	Cat#562739
Rat monoclonal anti-Mouse CD4-FITC clone GK1.5	BD Biosciences	Cat#557307; RRID: AB_396633
Rat monoclonal anti-Mouse CD8-PE-CF594 clone 53-6.7	BD Biosciences	Cat#562283; RRID: AB_11152075
Rat monoclonal anti-Mouse CD19-FITC clone 6D5	Biolegend	Cat#115506; RRID: AB_313641
Rat monoclonal anti-Mouse IgD-APC clone 11-26c.2a	BD Biosciences	Cat#560868; RRID: AB_10612002
Rat monoclonal anti-Mouse MHCII-APC-Cy7 clone M5/114.15.2	Biolegend	Cat#107628; RRID: AB_2069377
Rat monoclonal anti-Mouse CD135-BV421 clone A2F10	Biolegend	Cat#135313; RRID: AB_2562338
Armenian Hamster anti-Mouse CD48-BV711 clone HM48-1	Biolegend	Cat#103439; RRID: AB_2650824
Rat monoclonal anti-Mouse CD150-BV605 clone TC15-12F12.2	Biolegend	Cat#115927; RRID: AB_11204248
Mouse Monoclonal anti-PLZF	Santa Cruz Biotechnology	Cat# sc-28319; RRID: AB_2218941
Rabbit monoclonal anti-Cleaved Caspase3	Cell Signaling technology	Cat#9664; RRID: AB_2070042
Mouse monoclonal γH2AX clone JBW301	Millipore	Cat#05-63; RRID: AB_309864
Biotinylated Goat Anti-Rabbit IgG Antibody	Vector Laboratories	Cat#BA-1000; RRID: AB_2313606
Biotinylated Horse Anti-Mouse IgG Antibody, rat adsorbed	Vector Laboratories	Cat#BA-2001; RRID: AB_2336180
Rabbit polyclonal anti-p53	Vector Laboratories	Cat#VP-P956; RRID: AB_2335917
Rabbit polyclonal anti-phospho-Histone H3 (Ser10)	Cell Signaling technology	Cat#9701; RRID: AB_331535
Mouse monoclonal anti-p53 (1C12)	Cell Signaling technology	Cat#2524; RRID: AB_331743
Mouse monoclonal anti-Histone H3	Abcam	Cat#ab10799; RRID: AB_470239
Rabbit polyclonal anti-SMC1	Abcam	Cat#ab21583; RRID: AB_2192477
Rat monoclonal anti-BrdU	AbD Serotec	Cat#OBT0030; RRID: AB_609568
Mouse monoclonal anti-BrdU	Becton Dickinson	Cat#347580; RRID: AB_400326
Rabbit polyclonal anti-POLE4	This study	N/A
Mouse Monoclonal Anti-POLE2	Abcam	Cat#ab57298; RRID: AB_2166739
Rabbit polyclonal Anti POLE3	Bethyl	Cat#A301-245A; RRID: AB_890598
Rabbit polyclonal Anti POLE	Genetex	Cat#GTX132100
Rat monoclonal anti GINS1	Fitzgerald	Cat#10R-1766; RRID: AB_10809593
Rabbit polyclonal anti-POLD1	Bethyl	Cat#A304-007A; RRID: AB_2620355

(*Continued on next page*)

Continued

REAGENT or RESOURCE	SOURCE	IDENTIFIER
Rabbit polyclonal anti-POLD2	Abcam	Cat#ab38338; RRID: AB_2252592
Rabbit polyclonal anti-POLA1	Abcam	Cat#ab31777; RRID: AB_731976
Mouse monoclonal anti-MCM2	BD Biosciences	Cat#610701; RRID: AB_398024
Rabbit polyclonal anti-CDC45	Santa Cruz Biotechnology	Cat#sc-20685; RRID: AB_2078507
Rabbit polyclonal anti-GINS3	ProteinTech	Cat#15651-1-AP; RRID: AB_2247477
Rabbit monoclonal anti-pChk1 S345	Cell Signaling technology	Cat# 2348; RRID: AB_331212
Chemicals, Peptides, and Recombinant Proteins		
CldU	Sigma-Aldrich	Cat#C6891
EDTA-free Complete protease inhibitor cocktail	Roche	Cat#COEDTAF-RO
IdU	Sigma-Aldrich	Cat#I7125
EdU	Thermo Fisher Scientific	Cat#A10044
Biotin-Azide	Thermo Fisher Scientific	Cat#B10184
Low melting agarose	Sigma-Aldrich	Cat#A9414
PhosSTOP phosphatase inhibitor cocktail	Roche	Cat#PHOSS-RO
Trustain FcX	Biolegend	Cat#101319
Zombie Yellow dye	Biolegend	Cat#423104
Methocult	StemCell Technologies	Cat#M3434
Alizarin Red S	Sigma-Aldrich	Cat#A5533
Alcian Blue	Sigma-Aldrich	Cat#A5268
3,3′-diaminobenzidine (DAB) substrate	Vector Laboratories	Cat#SK-4100
Streptavidin Sepharose high performance	GE Healthcare	Cat#17-5113-01
CuSO4	SIGMA	Cat#PHR1477
Ribonuclease A	SIGMA	Cat# R5125
Sodium L-Ascorbate	SIGMA	Cat#A7631
BrdU	SIGMA	Cat#B5002
Propidium Iodide	SIGMA	Cat# P4170
Benzonase	Novagen	Cat#71206-3
GIEMSA stain	SIGMA	Cat#48900
DAPI	SIGMA	Cat#10236276001
Hydroxyurea	SIGMA	Cat#H8627
Thymidine	SIGMA	Cat#T9250
Critical Commercial Assays		
FiberPrep (DNA Extraction Kit)	Genomic Vision	Cat#EXTR-001
Lipofectamine RNAiMAX	Thermo Fisher	Cat#13778150
QIAprep Spin Miniprep Kit	QIAGEN	Cat#27106
RNeasy Mini Kit	QIAGEN	Cat#74106
Mouse Hematopoietic Stem and Progenitor Cell Isolation Kit	BD Biosciences	Cat#560492
ProLong Gold antifade with DAPI	Thermo Fisher	Cat#P36931
Click-iT EdU Alexa Fluor 488 Flow Cytometry Assay Kit	Thermo Fisher	Cat#C10425
Experimental Models: Mouse Strains		
$Pole4^{tm1(KOMP)Vlcg}$	This study	N/A
$Trp53^{tm1Brd}$	Donehower et al., 1992	N/A
Experimental Models: Cell Lines		
Mouse Embryonic Fibroblasts $Pole4^{-/-}$	This study	N/A
Mouse Embryonic Fibroblasts $Pole4^{-/-}p53^{-/-}$, $Pole4^{+/+}p53^{-/-}$	This study	N/A

(*Continued on next page*)

Continued

REAGENT or RESOURCE	SOURCE	IDENTIFIER
CTR1 and CTR2 (control unaffected patients)	This study and C.L., J.E. Murray et al., unpublished data	N/A
P1 and P2 (*POLE1* mutant cells)	This study and C.L., J.E. Murray et al., unpublished data	N/A
Oligonucleotides		
ON-TARGETplus Non-targeting Control Pool	Dharmacon	Cat#D-001810-10
ON-TARGETplus POLE siRNA	Dharmacon	Cat#L-020132-00
ON-TARGETplus POLE2 siRNA	Dharmacon	Cat#L-018612-02
ON-TARGETplus POLE3 siRNA	Dharmacon	Cat#L-008460-01
ON-TARGETplus POLE4 siRNA	Dharmacon	Cat#L-009850-02
Software and Algorithms		
Adobe Photoshop CC	Adobe	https://www.adobe.com/es/products/photoshop.html
Cell Profiler	Cell Profiler	http://cellprofiler.org/
ImageJ	NIH	https://imagej.nih.gov/ij/
Volocity 6.3	PerkinElmer	http://cellularimaging.perkinelmer.com/downloads/detail.php?id=14
GraphPad Prism 7	GraphPad	https://www.graphpad.com/
FlowJo	TreeStar	https://www.flowjo.com/solutions/flowjo/downloads

CONTACT FOR REAGENT AND RESOURCE SHARING

Further information and requests for reagents should be directed to and will be fulfilled by the Lead Contact, Simon Boulton (simon.boulton@crick.ac.uk).

EXPERIMENTAL MODEL AND SUBJECT DETAILS

Mouse strains

Mice deficient for POLE4 were generated using an VGB6 ES cell line *Pole4*$^{tm1(KOMP)Vlcg}$ available from KOMP repository (University of California, Davis) in which a deletion cassette containing a β-Galactosidase and a neomycin resistance was inserted in exon 1. This ES line is heterozygous for the *Pole4*$^{tm1(KOMP)Vlcg}$ insertion and therefore contains a WT allele of POLE4, which is sufficient to provide normal function of Polε based on our analysis in het MEF lines. The precise localization of the genetrap vector has been determined by sequencing using primer 5′-AAACCGCACTTCCAATTCTG-3′. *Pole4*$^{tm1(KOMP)Vlcg}$ ES cells were injected into C57BL/6Jax host blastocysts and implanted into pseudopregnant females. Chimeric mice were obtained and bred to C57BL/6Jax mice. The resulting heterozygous (*Pole4*$^{+/-}$) mice were bred to obtain homozygous *Pole4*$^{-/-}$. Genotyping of the offspring was confirmed by western-blot using a homemade antibody and PCR using the following primers (P4-common, 5′-GAGAGGCGTGGTCTCTACCC-3′; P4-WT, 5′-CACCAAGGCCTTTACTCTCG-3′; P4-mut 5′-ATCTCTCCTCTGCAGGACCA-3′). Homozygous *Pole4*$^{-/-}$ mice were also bred into an oubred background 129X1/SvJ;129S1/Sv; FVB/N. *Trp53*^{tm1Brd} were obtained from Allan Bradley (Donehower et al., 1992). Homozygous *Pole4*$^{-/-}$ mice were bred to *Trp53*$^{-/-}$ to obtain double heterozygous mice that were mated together to produce MEFs and double homozygous mice.

Cell lines

Sources of cell lines used in the study are listed in the reagent and resource table. Primary *Pole4*$^{+/+}$, *Pole4*$^{-/-}$, *Pole4*$^{+/+}$ *p53*$^{+/+}$and *Pole4*$^{+/+}$ *p53*$^{-/-}$ mouse embryonic fibroblasts were cultured at 37°C/ 5% CO_2/ 5% O_2 in Dulbecco's modified Eagle's medium (DMEM) (Invitrogen) supplemented with 15% fetal bovine serum (FBS; Sigma) and 1% penicillin-streptomycin (Invitrogen). The sex of the cells was not determined for this study. Human HeLa cells were cultured in DMEM 10% FBS (SIGMA) at 37°C/ 5% CO_2, while human dermal fibroblasts from controls (CTR1 and CTR2) and *POLE1* mutant patients (P1 and P2) were grown in AmnioMAX C-100 Complete Medium (Thermo Fisher Scientific) at 37°C/ 5% CO_2/ 5% O_2.

Clinical Synopsis of POLE1 patients

Mutations in *POLE1* were identified in Patient 1 and 2 by whole genome sequencing of cases with microcephalic dwarfism. A further 6 patients have since been identified from the same patient cohort. All had intrauterine growth retardation (IUGR), and severe

reduction in post-natal stature. Immunodeficiency, adrenal failure, and male genital hypoplasia were present in some, but not all cases. The molecular genetic identification of POLE1 mutations and the detailed clinical phenotype of all 8 cases will be documented in a separate manuscript (C.L., J.E. Murray et al., unpublished data).

Patient 1, was noted to have intrauterine growth retardation at 18/40 gestation on routine ultrasonography, and at birth weighed 926 g (born at 31 weeks gestation). At age 18; height 109.9cm, −9.6 standard deviations (s.d.) below the mean, head circumference −5.1sd. He has low-set posteriorly rotated ears, a small chin, and long thin nose with short neck. He has had CMV pneumonitis, and an EBV-driven hemophagocytic lymphohistiocytosis that resulted in bone marrow transplant. CD4 lymphopenia and hypogammaglobinemia were evident prior to transplantation. He has also had endocrinological complications with primary adrenal failure, hypopituitarism, and insulin resistance. He had also a younger sister who died of HSV infection.

Patient 2 was born at term with a birth weight of 2.5kg. At age 7, height was 93.8cm, −5.4sd; head circumference −2.7sd. He has a similar facial appearance to P1, with small low-set ears, micrognathia, and down-slopping palpebral fissures. No increased susceptibility to infections or immunodeficiency has been reported to date, although he has suffered from recurent otitis media associated with conductive hearing loss. He has recently acquired hearing aids. He also has primary adrenal failure (glucocorticoid deficiency), an accessory central tooth (extracted), a history of severe infantile eczema, cryptorchidism and hypospadias, and severe feeding difficulties due to oral aversion alongside a moderate learning disability. He also has documented osteopenia.

Molecular Genetics of POLE1 patients

Patient 1 is compound heterozygous for the truncating mutation, c.2010dupC, p.Phe672Valfster11, and the intronic variant, c.1686+32C > G.

Patient 2 is compound heterozygous for the same intronic variant, c.168+32C > G in conjunction with an essential splice site mutation, c.62+1G > A.

RT-PCR analysis of patient RNA and *in vitro* mini-gene splicing studies have established that the +32 intronic variant significantly impairs splicing of intron 15, resulting in retention of 47bp intronic sequence, that results in a frameshift consequently predicted to result in nonsense-mediated decay (manuscript in preparation, CL, JM, APJ). Molecular genetic findings predict substantial reduction of POLE1 protein levels and impaired Pol-epsilon function in both patients.

Footnote: Sequence variants annotated on the basis of reference sequence NM_006231.3/NP_006222.2

METHOD DETAILS

Mice breeding and experiments

At least 3 breedings were continuously mated to establish Mendelian ratios and fertility.

For longevity studies, mice were allowed to age and observed for development of disease. The endpoint of the study was set at 21 months but if they appeared unhealthy or got palpable tumors beforehand, animals were sacrificed. They were then subjected to full necropsy.

For blood sampling, mice were placed in a heating chamber for 10 minutes. Then, tail prick was performed and blood was collected into EDTA coated end-to-end capillaries (Sarstedt, 19.447) and transferred to EDTA coated microvettes (Sarstedt, 20.1278). 10μl of blood (duplicates) was used to perform a full blood differential on the VetABC+ blood analyzer (Horiba).

All animal experimentations were undertaken in compliance with UK Home Office legislation under the Animals (Scientific Procedures) Act 1986.

Histology, immunohistochemistry

For histology and post-mortem tissues, samples were fixed in 10% Neutral buffered formalin (NBF), paraffin embedded, sectioned at 4μm and stained with hematoxylin and eosin.

For immunohistochemistry, samples were prepared using standard methods. In brief, tissue sections were processed for staining by microwaving in 0.01M citrate buffer, pH 6. After incubation with primary antibodies (PLZF, Santa Cruz sc28319; p53, Vector VP-P596; Cleaved Caspase3, Cell Signaling 9664; gH2AX, Millipore 05-636;), samples were incubated with biotinylated secondary antibody (Vector) followed by incubation with Avidin Biotin Complex (Vector, Biotinylated Goat anti-rabbit IgG BA-1000; Biotinylated Horse anti-Mouse (Rat adsorbed) BA-2001); slides were developed in 3,3′-diaminobenzidine (DAB) substrate (Vector, SK-4100) and counterstained in hematoxylin. Embryos section images were acquired at 40X using a Aperio AT2 scanner (Leica). Stainings were quantified using Cell Profiler software. Testis and lymphomas section images were acquired at 20X using an Axio Scan.Z1 (Zeiss) and stainings were quantified using ImageJ software on serial sections and tubules with similar diameter.

Alcian blue/Alizarin red staining

Embryos were stained following a modified version of the protocol published by Rigueur and Lyons, 2014. Briefly, embryos were collected at 15.5dpc and extraembryonic membranes were removed. Then embryos were fixed overnight in 70% ethanol at room temperature and incubated in 95% ethanol for 1 h. Ethanol was replaced by acetone to remove excess of fat and let overnight at room temperature. Embryos were then stained with 0.03% Alcian Blue (Sigma, A5268) for 24h and then de-stained in 70% ethanol overnight. They were cleared in 1% KOH for 3h and then counterstained in 0.05% Alizarin Red S (Sigma, A5533) for 4h at room temperature. Embryos were cleared in 1% KOH overnight and then transferred to 100% glycerol for long-term storage.

Pictures were taken on a white background using a Stemi SV6 stereomicroscope (Zeiss) and bone were measured using ImageJ software.

MicroCT scan

Mice were anaesthetized with 2.5% isofluorane and microCT images were taken using a SkyScan 1176 microCT scanner (Bruker Micro CT, Kontich, Belgium). Anaesthesia was maintained with 2% isoflurane during the scan. X-ray projection images were acquired using a source voltage of 50kV and a source current of 500μA and a 0.5mm aluminum filter over a 180° trajectory with a rotation step size of 0.7°. Other imaging parameters were: pixel size = 36.04μm, frame averaging = 2, exposure time = 60ms. Scan time was 3min 35sec per sub scan. Each lower body scan consists of 6 sub scans. Reconstruction was performed using NRecon software (version 1.6.8) Reconstruction parameters: smoothing set to 4, ring artifact correction set to 4 and beam hardening correction set to 35%. A variable post alignment compensation and dynamic range of 0.001973-0.092057 of the X-ray attenuation coefficient were applied.

Rotarod experiments

Animals in home cage were placed in testing room for at least 1hr before testing to minimize effects of stress on behavior during testing. Animals from the same cage were placed in separated lane of the apparatus (AccuRotor 4-Channel RotaRod) and trained to walk on a 5rpm rotating rod for 60 s (3 trials separated by 10 min intervals). Then animals were placed on the rod again and the apparatus was set to accelerate from 4 to 40rpm in 300 s. Procedure was repeated 3 times separated by at least 10 min intervals. Any animals that completed a full passive rotation around the rod were trialed a fourth time. The latency to fall was recorded and plotted on graphs. Young animals were tested at 3-4 months old and aged animals around 12 months old. Body weight was checked to ensure that it won't affect their gait.

Cobblestone area forming cell assay (CFAC)

$2x10^5$ bone marrow cells were plated (in triplicates) for colony-forming assay using MethoCult (StemCell Technologies, M3434) following the manufacturer's instructions. Cells were incubated at 37°C with 5% CO2 and 20% O2 for 12 days, before colonies were identified and counted manually as CFU (Colony Forming Unit) -G (Granulocytes), -M (Macrophages), -GM (Granulocyte, Macrophage), GEMM (Granulocyte, Erythroid, Macrophage, Megakaryocyte), BFU-E (Burst Forming Unit– Erythroid).

Immunophenotyping

Lymphocyte populations were analyzed by flow cytometry in single cell suspensions from spleen. Briefly, spleens were mashed up through 70μm cell strainer with the end of a syringe plunger and cells were resuspended as single cells in RPMI + 2%FBS. Non-specific antibody binding was blocked by using an anti-mouse CD16/32 antibody (Biolegend, Trustain FcX, 101319). Then cells were stained using the following combination for T lymphocyte analysis (CD5-BV421 (BD, 562739), CD4-FITC (BD, 557307), CD8-PE-CF594 (BD, 562283) and for B lymphocyte analysis (CD5-BV421 (BD, 562739), CD19-BV510 (BD, 562956), IgD-APC (BD, 560868), MHCII-APC-Cy7 (Biolegend, 107628)). Viability was determined by staining the cells with Zombie Yellow dye (Biolegend, 423104).

Hematopoietic stem cells (HSCs) were analyzed from bone marrow samples. Femur and tibia were crushed in RPMI + 2% FBS in a mortar and passed through cell strainers to achieve single cells suspension. Then cells were stained using the BD Mouse Hematopoietic Stem and Progenitor Cell Isolation Kit (BD, 560492) following the manufacturer's procedure. Briefly, non-specific antibody binding was blocked by using an anti-mouse CD16/32 antibody (BD, clone 2.4G2). then the cells were stained with Lin-APC (CD3, clone 145-2C11; CD45R (B220), clone RA3-6B2; Ly6C and Ly6G (Gr1), clone RB6-8C5; CD11b (Mac1) clone M1/70; TER-119, clone TER-119), Sca-1 PE-Cy7 (Clone D7), c-Kit-PE (Clone 2B8), CD34-FITC (Clone RAM34), CD135-BV421 (Flt3, Biolegend, clone A2F10, 135313), CD48-BV711 (Biolegend, clone HM48-1, 103439), CD150-BV605 (Biolegend, clone TC15-12F12.2, 115927). Cells viability was determined by staining with Zombie Yellow dye (Biolegend, 423104).

Data were collected on an LSRFortessa flow cytometer (Becton Dickinson) and were analyzed with FlowJo software (TreeStar).

Western blot analysis of embryos, mouse tissues and cells

Embryos were snap frozen at −80deg and resuspended in benzonase buffer (50mM Tris-HCl pH7.5, 50mM NaCl, 2mM MgCl2, 0.5% NP-40, 50U/ml benzonase, 1x protease inhibitor, 1x phosphatase inhibitor) and incubated at 4°C for 30 min. NaCl was added to the sample at a final concentration of 150mM. After 30min incubation, cell lysates were clarified by centrifugation.

Mice tissues were snap-frozen in liquid nitrogen and subsequently lysed in RIPA buffer (150 mM NaCl, 100 mM Tri pH 7.5, 1% NP-40, 0.1% SDS, 0.5% sodium deoxycolate) containing protease and phosphatase inhibitors (ROCHE) using the precellys 24 tissue disruptor (Berlin technologies).

Cultured cells from *Pole4*$^{+/+}$ and *Pole4*$^{-/-}$ embryos (MEFs) or control and *POLE1* mutant patients were lysed in RIPA containing protease and phosphatase inhibitors (ROCHE). Lysates were clarified by centrifugation (12.300 rpm 30 min at 4°C) and protein concentration was estimated by BRADFORD assay (BIORAD). Equal amounts of proteins were loaded on NuPAGE 4%–12% Bis-Tris gels and transferred onto nitrocellulose membrane. Membranes were blocked in 5% milk in PBST (PBS-Tween 0.1%) and incubated with primary antibodies and HRP-conjugated secondary antibodies.

Mouse Embryonic Fibroblasts (MEFs) isolation and culture

Pole4$^{+/-}$ mice in mixed or C57BL/6 background were mated. Pregnant females at 13.5 days gestation were subjected to euthanasia under anesthesia, followed by uterine dissection to isolate individual embryos. Each embryo was washed in PBS followed by removal of head (used for embryo genotyping) and internal organs (heart and liver). The embryo body was minced with sterile razor blades and incubated in trypsin at 37°C for 20 min, followed by gentle pipetting of the trypsin digest. Cell suspension was pelleted, resuspended and plated in 10 cm dishes (now considered passage 0) in DMEM (Dulbecco's modified Eagle's medium (DMEM) supplemented with 15% FBS (SIGMA) and 50μg/ml penicillin-streptomycin, 2mM L-glutamine. Once subconfluent, a standard 3T3 protocol was followed: every 3 days cells were trypsinized, counted using cellometer Auto 2000 (Nexcelom Bioscience) to determine the number of Population doublings (PD) and then replated at a fixed density (8x10^5 cells per 100-mm dish) The accumulation of population doubling level (PDL) was calculated using the formula $\Delta PDL = \log(nh/ni)/\log 2$, where ni is the initial number of cells and nh is the cell number at each passage.

Immunofluorescence stainings

For indirect immunofluorescence stainings, cells were seaded on coverslips and fixed in 4% paraformaldehyde. After permeabilization with 0.5% Triton X-100 (5 min on ice), coverslips were blocked in 1% BSA/PBS and incubated with the following primary antibodies diluited in 0.5% BSA/PBS: anti-H2AX phosphorylated on Ser139 (gH2AX) (Millipore), −53BP1, (Novus Biologicals), for 1h at room temperature. Coverslips were then washed 3 times in PBS and incubated with Alexa Fluor 488 goat anti-rabbit or rabbit anti-mouse antibodies (Invitrogen) for 40 min at room temperature. After DAPI counterstaining, coverslips were mounted in Glycerol/PBS (1:1) and observed with Axio Imager.M2 (ZEISS) using the Volocity 6.3 software.

Chromatin fractionation

Chromatin fractionation experiments were performed as described in Bellelli et al. (2014). Cells in mid-esponential phase of growth were washed once in ice-cold 1X phosphate-buffered saline (PBS) and lysed in ice-cold CSK (10 mM PIPES, pH 6.8, 100mM NaCl, 300 mM sucrose, 1mM MgCl2, 1 mM EGTA, 1mM DTT) buffer containing 0.5% Triton X-100 (Pierce Biotechnology) and protease and phosphatates inhibitors (ROCHE) for 10 min on ice. Chromatin-bound and un-bound proteins were separated by low speed centrifugation (3,000 rpm, 3 min at 4°C). The pellett (chromatin fraction) was washed in CSK 0.5% Triton and resuspend in Laemmli buffer 1X. Total fraction was obtained by direct cell lysis in 1X Laemmli buffer. For each fraction, protein amounts deriving from comparable number of cells were analyzed by SDS-PAGE and western blotting.

DNA fiber stretching assay

DNA fiber assay was performed as described in Bellelli et al., 2014. Pole4$^{+/+}$ and Pole4$^{-/-}$ MEFs, as well as human dermal fibroblast from controls and *POLE1* mutant patients, were pulse labeled with 20 μM CldU for 20 min and subsequently pulse labeled with 200 μM IdU for 20 min. Cells were trypsinized, washed in PBS, counted and resuspended at a concentration of 5x 10^5 in PBS. 2.5 μL of cell suspension were spotted on clean glass slides and lysed with 7.5 μL of 0.5% SDS in 200 mM Tris-HCL, pH 7.4, 50 mM EDTA (10 min, R.T.). Slides were tilted (15° to horizontal), allowing a stream of DNA to run slowly down the slide, air-dried and then fixed in methanol/acetic acid (3:1) for 15 min at R.T. Acid-treated slides (30 min R.T.) were blocked in 1% BSA/PBS for 30 min at R.T. and incubated with rat anti-BrdU monoclonal antibody (1:1000 overnight; AbD Serotec) and mouse anti-BrdU monoclonal antibody (1:500 1h R.T.; Becton Dickinson). After 3 washes in PBS, slides were incubated with a mixture of Alexa Fluor 488 rabbit anti-mouse and Alexa Fluor 594 goat anti-rat antibodies (1:500 R.T.; Invitrogen) for 40 min at room temperature and mounted in PBS/Glycerol 1:1. Fibers were then examined using Axio Imager.M2 (ZEISS) with 60x oil immersion objective and the Volocity 6.3 software. For quantification, at least 500 replication structures were counted per experiment.

For analysis of interorigin distance upon Hydroxyurea (HU) treatment, *Pole4*$^{+/+}$ and *Pole4*$^{-/-}$ MEFs, as well as human dermal fibroblast from controls and *POLE1* mutant patients, were incubated for 3 hours in media containing 100 mM HU and subsequently pulsed labeled first in media containing HU (100 μM) and CldU (20 μM) for 30 min and then HU (100 μM) and IdU (200 μM) for 30 min. Cells were processed and stained as previously described for standard fiber assay. All fiber stretching assay experiments were performed at least in triplicate.

BrdU and EdU FACS

For EdU/DAPI FACS analysis, *Pole4*$^{+/+}$ and *Pole4*$^{-/-}$ MEFs (passage 3) were labeled and processed using the Click-iT EdU Flow Cytometry Cell Proliferation Assay (Thermo Fisher). Cells were pulse labeled for 30 min with 10 μM EdU and fixed in 4% paraformaldehyde, before being permeabilized in PBS-Triton 0.5% and washed in 1% BSA. Cells were then resuspended in Click-iT reaction cocktail containing Alexa Fluor 488 Azide and incubated for 30 min at R.T. After being washed, cells were finally counterstained for DNA content by DAPI (1 mg/ml) and analyzed using a Flow cytometry analyzer LSRII (Becton Dickinson).

For BrdU/DNA content analysis of human dermal fibroblasts derived from control or *POLE1* mutant patients, cells were pulse labeled with 10 μM BrdU (SIGMA) for 30 min, washed and released or not in normal media for 4, 8, 12 or 16 hours to analyze S-phase progression. Cell were trypsinized, pelleted and fixed in ice-cold 70% ethanol. After washing, cells were resuspended in 500 mL of 2M HCl and incubated at R.T for 30 min with occasional mixing. Cells were pelleted, washed to remove excessive acid and incubated with anti-BrdU antibody for 20 min at R.T. and rabbit anti-mouse FITC-conjugated (DAKO) for 20 at R.T. in the dark. Cells were finally washed, resuspended in PBS-T containing RNase A and Propidium Iodide and analyzed using a Flow cytometry analyzer LSRII (Becton Dickinson).

Chromosome spreads and GIEMSA staining

Cells in mid-exponential phase of growth were incubated in colcemid (10 mg/ml) for 2-4 hours. After shake off, floating cells were collected, pelleted and resuspended in 2 mL of media. Upon addition of 4 mL of deionized water, cells were gently mixed and incubated for 6 min at R.T. before being fixed on ice in methanol/acetic acid (3:1). Fixed cells were washed again in fixative buffer and resuspended 200 mL of fresh fixative buffer and spreaded on clean slides by gentle dropping. Dried slides were subsequently incubated in a solution of 6% GIEMSA/PBS for 7 min, washed in PBS and deionized water. Slides were finally mounted with DPX mounting media and analyzed using Axio Imager.M2 (ZEISS) with 60x oil immersion objective and the Volocity 6.3 software.

iPOND (isolation of Proteins on Nascent DNA) and iPOND SILAC Mass Spect

iPOND was performed according to standard protocols (Sirbu et al., 2011). *Pole4*$^{+/+}$ and *Pole4*$^{-/-}$ MEFs were pulse labeled with 10 μM EdU (5-ethynyl-2′-deoxyuridine, Invitrogen) for 10 min. After washing in normal media, cells were released or not for 30 min in media containing 10 μM thymidine (SIGMA). Cells were then fixed in 1% Formaldehyde (SIGMA) in PBS for 20 min at R.T. Crosslinking was subsequently quenched by addition of Glycine to a final concentration of 0.125M for 10 min at R.T. Cells were scraped, pelleted, washed 3 times in PBS and stored at −80. Frozen pellets were resuspended in 0.25% Triton/PBS and incubated at R.T. for 30 min. After washing in 0.5% BSA/PBS and PBS, pellets were resuspended in Click reaction cocktail cointaining 10 μM Biotin-Azide (Invitrogen), 10 mM Na Ascorbate (SIGMA) and 2mM CuSO4 (SIGMA) and incubated for 1h at R.T. Controls were resuspended in the same buffer containing DMSO instead of Biotin-Azide. After washing in 0.5% BSA/PBS and PBS, pellets were lysed in RIPA buffer (150 mM NaCl, 100 mM Tri pH 7.5, 1% NP-40, 0.1% SDS, 0.5% sodium deoxycolate) containing protease and phosphatase inhibitors (ROCHE) and sonicated using a BIORUPTOR sonicator in 1.5 mL Eppendorf tubes (20-25 cycles at 30 s on, 30 s off setting). Lysates were clarified by high speed centrifugation (13.200 rpm, 15 min at 4 C) and incubated with streptavidin Sepharose beads (GE Healthcare) for 16 hours. After being washed in RIPA and 1M NaCl, beads were resupended in 2X Laemmli buffer, incubated for 25 min at 99°C and loaded on 4%–12% NUPAGE Bis-Tris gels for SDS-PAGE analysis.

For iPOND SILAC Mass spectrometry, *Pole4*$^{+/+}$ and *Pole4*$^{-/-}$ embryonic cells were incubated in SILAC DMEM supplemented with 10% dialyzed FBS (SIGMA), 100 mg/L [$^{12}C_6$]arginine, and [$^{12}C_6$]lysine (light) or [$^{13}C_6$,$^{15}N_4$]l-arginine and [$^{13}C_6$,$^{15}N_2$]l-lysine (heavy) with 200 mg/L l-proline, at passage 0, amplified to passage 2 and subjected to iPOND as previously described. To this aim, cells grown in heavy and light media were fixed, quenched and collected in the same tube to obtain a single mix. After iPOND, pellet lysis in RIPA, samples sonication and streptavidin beads capture, the immunoprecipitated material was subjected to SDS-PAGE. Coomassie-stained polyacrylamide gel slices were excised from SDS-PAGE gels using a scalpel into a 96 well plate and processed for mass spectrometry using the Janus liquid handling system (PerkinElmer). Briefly, the excised protein gel pieces were placed in individual wells of a 96-well microtiter plate and destained with 50% v/v acetonitrile and 50 mM ammonium bicarbonate, reduced with 10 mM DTT, and alkylated with 55 mM iodoacetamide. After alkylation, the samples were digested with trypsin (Promega), overnight at 37°C. The resulting peptides were extracted in 1% v/v formic acid, 2% v/v acetonitrile. Digests were subsequently analyzed by nano-scale capillary LC-MS/MS. Peptide mixtures were separated on a 50 cm, 75um I.D. EasySpray C_{18} LC-MS column over a 30 minutes gradient and eluted directly into the LTQ Orbitrap Velos or Orbitrap Fusion Lumos (Thermo Scientific) mass spectrometer. The mass spectrometer was operated in data dependent mode with the most intense multiply charged precursor ions fragmented in the linear ion trap using collision-induced dissociation. Raw mass spectrometric data was processed in MaxQuant (Nature Biotechnology 26, 1367 - 1372 (2008)) (version 1.5.2.8) for protein identification and SILAC quantification, the database search was performed using the Andromeda search engine against the *Mus musculus* canonical sequences downloaded from UniProtKB (release 2012_08).

POLE4 Antibody generation

Policlonal anti-POLE4 antibody was generated by immunizing rabbits with full length un-tagged human POLE4 obtained by standard E. Coli expression methods. Serum was affinity purified by chromatography using a GST-POLE4 column and used at a 1:1000 concentration for western blotting. Rabbits immunization and antibody purification was performed by Cambridge Research Biochemicals (Billingham, UK) in accordance with standard rules and procedures.

Analysis of Hydroxyurea and Ionizing radiation sensitivity

Pole4$^{+/+}$ and *Pole4*$^{-/-}$ MEFs as well as *POLE1* mutant and control cells were treated with increasing doses of Hydroxyurea (SIGMA) for 24 hours or irradiated using a GSR D1 ^{137}Cs irradiator with 2 or 4 Gy. After washing, cells were left growing in standard media for 5 days and viable cells were analyzed using Cellometer Auto 2000 (Nexcelom Bioscience).

QUANTIFICATION AND STATISTICAL ANALYSIS

Statistics, including statistical tests used, number of events quantified, standard deviation standard error of the mean, and statistical significance are reported in the figures and in the figure legends (Kaplan–Meier plots for survival and significance calculation using Log-rank (Mantel–Cox) test, unpaired t test for staining quantification statistics). Statistical analysis has been performed using GraphPad Prism7 software (GraphPad) and statistical significance is determined by the value of $p < 0.05$.

Molecular Cell, Volume *70*

Supplemental Information

Polε Instability Drives Replication Stress, Abnormal Development, and Tumorigenesis

Roberto Bellelli, Valerie Borel, Clare Logan, Jennifer Svendsen, Danielle E. Cox, Emma Nye, Kay Metcalfe, Susan M. O'Connell, Gordon Stamp, Helen R. Flynn, Ambrosius P. Snijders, François Lassailly, Andrew Jackson, and Simon J. Boulton

Figure S1

A.

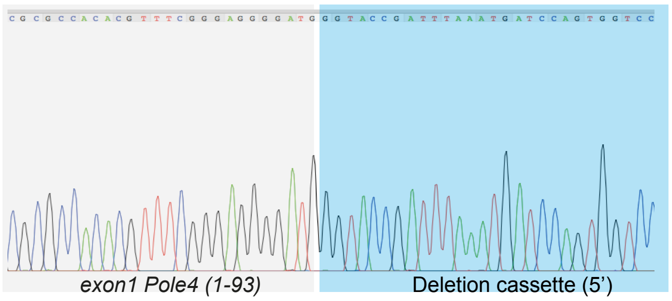

B.

	Pole4 (inbred)			
	+/+	+/-	-/-	Total
#	91 (63)	161 (127)	1 (63)	253
%	36.0 (25)	63.6 (50)	0.4 (25)	100

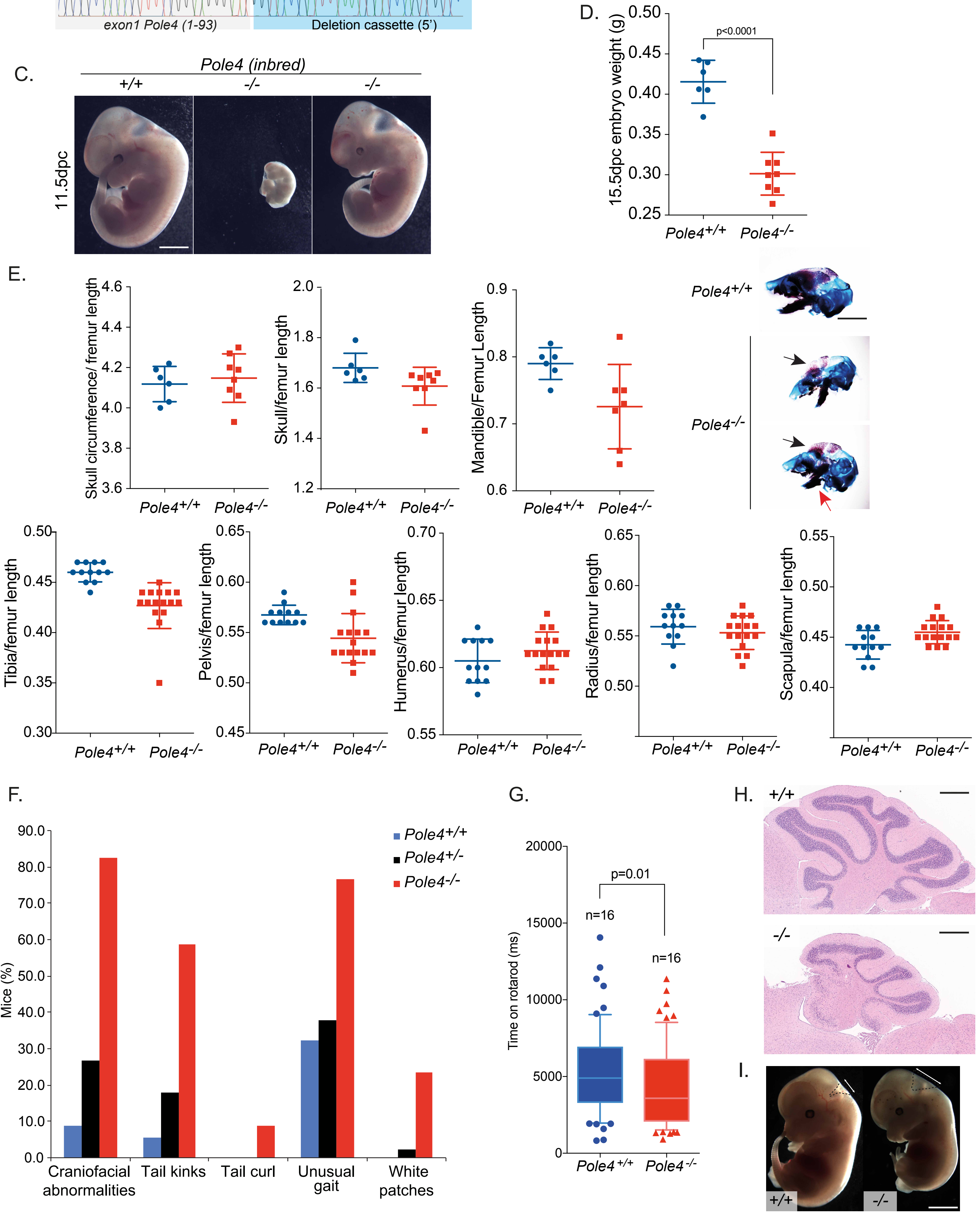

Figure S1: (Related to Figure 1)

(A) Sequencing data showing the insertion of the deletion cassette in exon1 of *Pole4* gene. (B) *Pole4*$^{+/+}$, *Pole4*$^{+/-}$ and *Pole4*$^{-/-}$ mice Mendelian ratios in C57BL/6 background. Numbers in brackets represent expected number or percent. (C) Representative images of inbred *Pole4*$^{+/+}$ and *Pole4*$^{-/-}$ embryos at 11.5dpc. Bar=0.5mm (D) Weight analysis of *Pole4*$^{+/+}$ and *Pole4*$^{-/-}$ embryos at 15.5dpc. Error bars represent ±standard error of the mean (SEM) of n= *Pole4*$^{+/+}$ and n= *Pole4*$^{-/-}$. Significance: t-test, p<0.0001. (E) Body and bones measurements relative to femur length during development in utero at 15.5dpc. At least 3 embryos were used per analysis. Alcian blue/Alizarin red staining of *Pole4*$^{+/+}$ and *Pole4*$^{-/-}$ embryos head at 15.5dpc. The black arrow shows the bulging forehead and the red arrow indicates the shortening of the mandible. Bar=3mm. (F) Frequency of *Pole4*$^{+/+}$ and *Pole4*$^{-/-}$ mice displaying abnormal phenotypes such as craniofacial abnormalities, tail kinks or curl, unusual gait and white patches. (G) Rotarod experiment testing 12 months old *Pole4*$^{+/+}$ and *Pole4*$^{-/-}$ mice coordination. Note the decreased time spent on the rotating rod by *Pole4*$^{-/-}$ mice compared to their wild type littermates. Error bars represent ±standard error of the mean (SEM) of n=16 *Pole4*$^{+/+}$ and n=16 *Pole4*$^{-/-}$. Significance: *t*-test, p=0.01. (H) Sagittal section of cerebellum from *Pole4*$^{+/+}$ and *Pole4*$^{-/-}$ mice. Note the reduced size and decrease in the number of lobule in the mutant section. Sections are stained by hematoxylin and eosin, bar=500µm. (I) Whole-mount lateral images of embryos at 13.5dpc. White arrows show the expanded roof of fourth ventricle, bar=1mm.

Figure S2

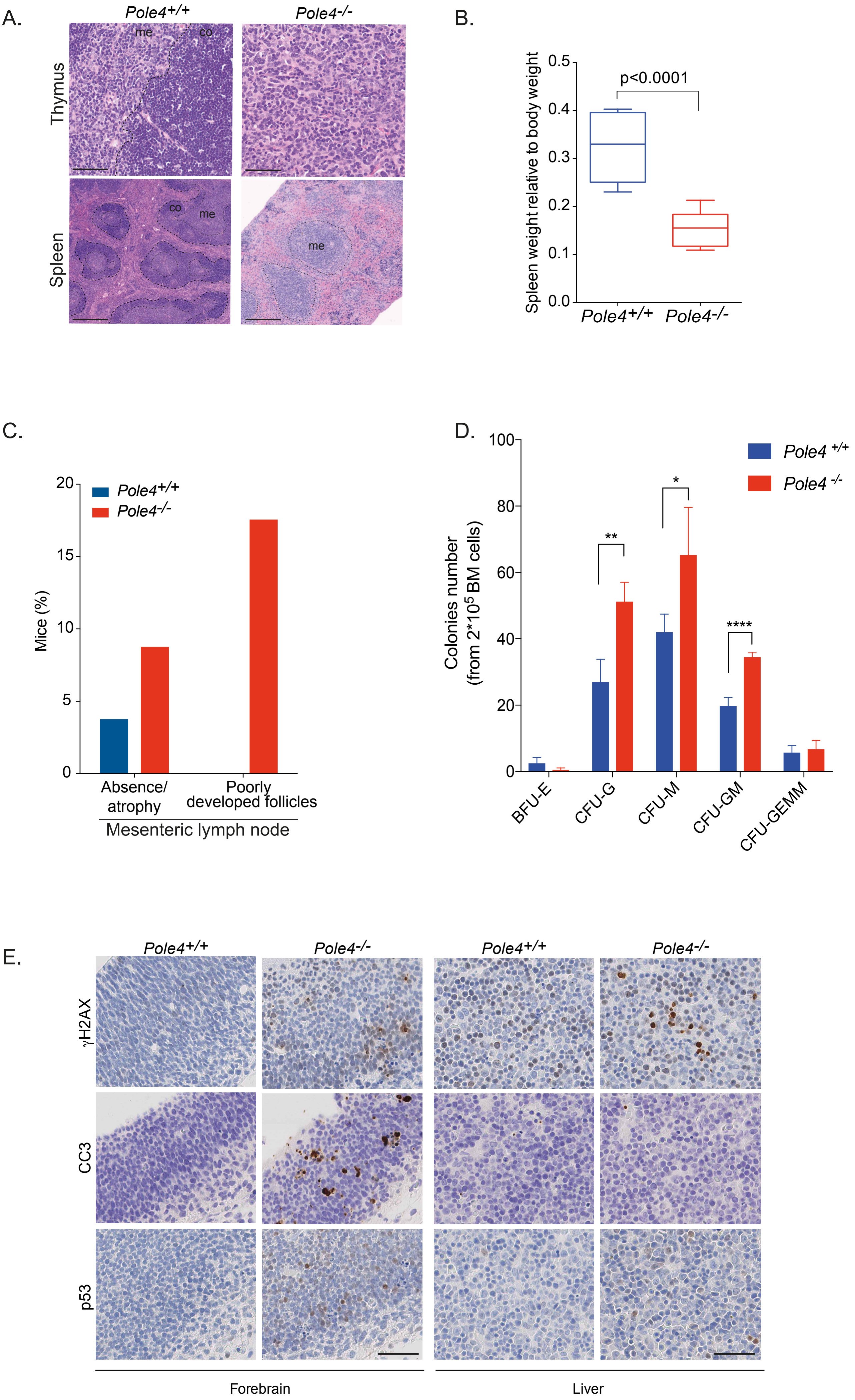
A.
Pole4+/+
Pole4-/-
Thymus
Spleen
me
co
B.
Spleen weight relative to body weight
p<0.0001
0.0
0.1
0.2
0.3
0.4
0.5
Pole4+/+
Pole4-/-
C.
Mice (%)
Pole4+/+
Pole4-/-
0
5
10
15
20
Absence/ atrophy
Poorly developed follicles
Mesenteric lymph node
D.
Colonies number (from 2*10^5 BM cells)
Pole4 +/+
Pole4 -/-
0
20
40
60
80
100
**
*
****
BFU-E
CFU-G
CFU-M
CFU-GM
CFU-GEMM
E.
Pole4+/+
Pole4-/-
Pole4+/+
Pole4-/-
γH2AX
CC3
p53
Forebrain
Liver

Figure S2: (Related to Figure 2)

(A) Representative sagittal section of *Pole4*$^{+/+}$ and *Pole4*$^{-/-}$ mice thymus and spleen. me: medulla, co: cortex. Bar= 300μm for thymus and 200μm for spleen. (B) Spleen weight relative to body weight. Significance: *t*-test, p<0.0001. (C) Frequency of *Pole4*$^{+/+}$ and *Pole4*$^{-/-}$ mice presenting abnormal mesenteric lymph nodes. (D) Cobblestone area forming cells assay of bone marrow (BM) cells from *Pole4*$^{+/+}$ and *Pole4*$^{-/-}$ mice plated and grown in Methocult media. Colonies of hematopoietic progenitors BFU-E, CFU-GEMM, CFU-GM, CFU-G or CFU-M were counted 12 days after plating $2x10^5$ cells. Bone marrow from 3 animals was used and plated in triplicates. Significance: *t*-test, for CFU-GM, ****: p<0.0001; for CFU-G, **: p=0.0016; for CFU-M, *p=0.0232. (E) Representative images of γH2AX, Cleaved caspase 3 and p53 immunohistochemistry in forebrain and liver section of 13.5dpc embryos. Nuclei have been counterstained with hematoxylin. Bar=50μm.

Figure S3

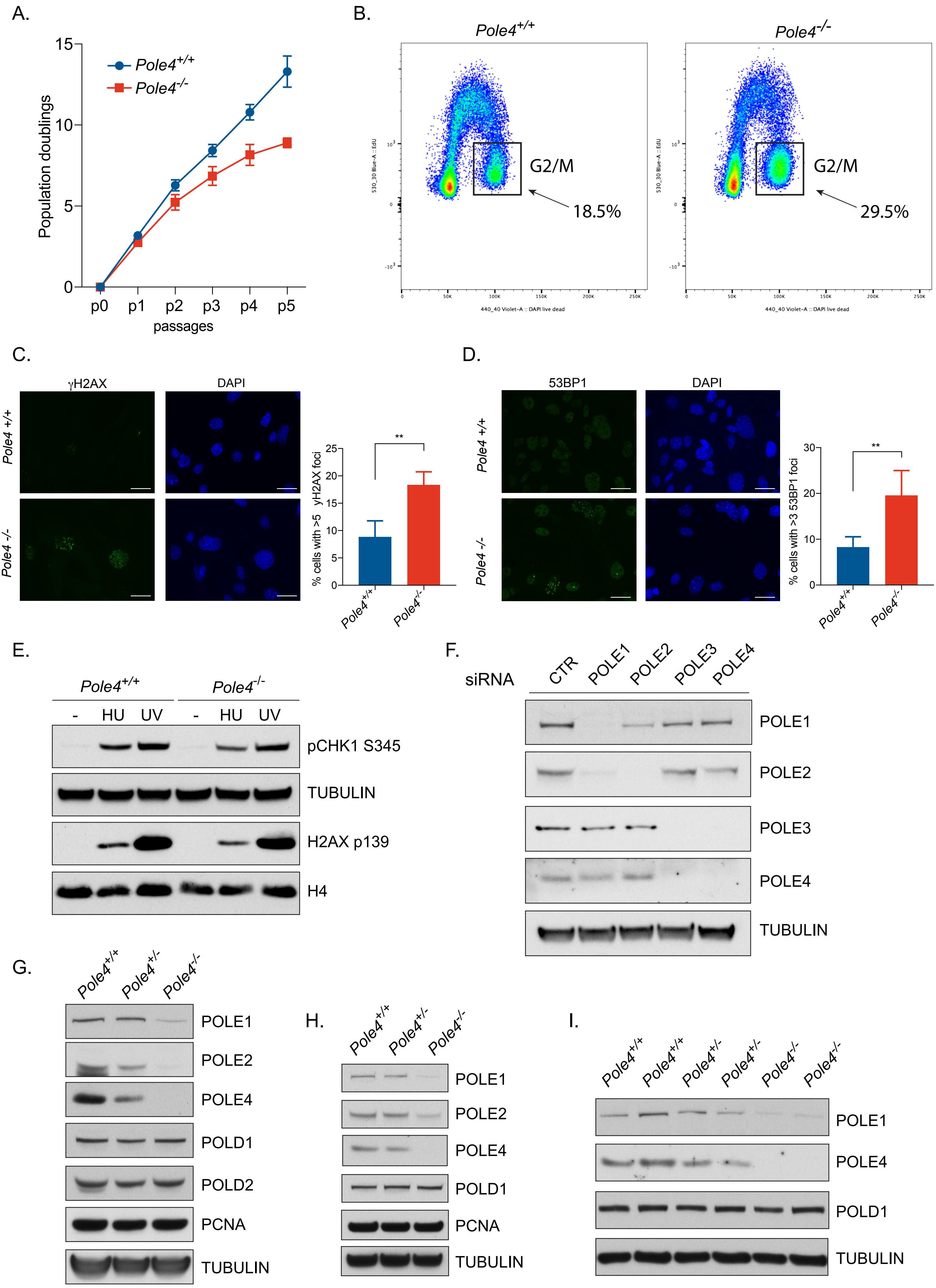
A.
Pole4+/+
Pole4-/-
Population doublings
15
10
5
0
p0
p1
p2
p3
p4
p5
passages
B.
Pole4+/+
Pole4-/-
G2/M
18.5%
G2/M
29.5%
C.
γH2AX
DAPI
Pole4 +/+
Pole4 -/-
% cells with >5 γH2AX foci
**
Pole4+/+
Pole4-/-
D.
53BP1
DAPI
Pole4 +/+
Pole4 -/-
% cells with >3 53BP1 foci
**
Pole4+/+
Pole4-/-
E.
Pole4+/+
Pole4-/-
- HU UV - HU UV
pCHK1 S345
TUBULIN
H2AX p139
H4
F.
siRNA
CTR
POLE1
POLE2
POLE3
POLE4
POLE1
POLE2
POLE3
POLE4
TUBULIN
G.
Pole4+/+
Pole4+/-
Pole4-/-
POLE1
POLE2
POLE4
POLD1
POLD2
PCNA
TUBULIN
H.
Pole4+/+
Pole4+/-
Pole4-/-
POLE1
POLE2
POLE4
POLD1
PCNA
TUBULIN
I.
Pole4+/+
Pole4+/+
Pole4+/-
Pole4+/-
Pole4-/-
Pole4-/-
POLE1
POLE4
POLD1
TUBULIN

Figure S3. (Related to Figure 3).

(A) Analysis of proliferation of *Pole4*$^{+/+}$ and *Pole4*$^{-/-}$ MEFs, measured as accumulation of population doublings. Cells were cultured in low oxygen conditions (5%) according to a standard 3T3 protocol; data are shown as mean +/- SD of 4 different experiments. (B) Representative EdU/DAPI flow cytometry profile of *Pole4*$^{+/+}$ and *Pole4*$^{-/-}$ MEFs. (C) Left: representative γH2AX immunofluorescence staining of *Pole4*$^{+/+}$ and *Pole4*$^{-/-}$ MEFs. DAPI was used for nuclear counterstaining. Right: Bar graph showing percentage of *Pole4*$^{+/+}$ and *Pole4*$^{-/-}$ MEFs with >5 γH2AX foci (** p< 0.01); Error bars ±standard deviation (SD) are included; Scale bar, 30μM. (D) representative 53BP1 immunofluorescence staining of *Pole4*$^{+/+}$ and *Pole4*$^{-/-}$ MEFs. DAPI was used for nuclear counterstaining. Right: Bar graph showing percentage of *Pole4*$^{+/+}$ and *Pole4*$^{-/-}$ MEFs with >3 53BP1 foci (** p< 0.01); Error bars ±standard deviation (SD) are included; Scale bar, 30μM. (E) Western blot analysis of total cell extracts from *Pole4*$^{+/+}$ and *Pole4*$^{-/-}$ MEFs exposed to 2 mM Hydroxyurea (HU) or 20J/m2 ultraviolet rays (UV) for 4 hours. pCHK1 S345 and γH2AX (pH2AX S139) were used as markers of checkpoint activation. Tubulin and H4 were used as loading controls. (F) Western blot analysis of HeLa cell lysates, subjected to silencing of Polε complex subunits, using the indicated antibodies. Tubulin was used for normalization. (G-H-I) Western blot analysis of testis (G), embryo (H) or MEFs (I) extracts from *Pole4*$^{+/+}$, *Pole4*$^{+/-}$ and *Pole4*$^{-/-}$ mice using the indicated antibodies. Tubulin was used as loading control.

Figure S4

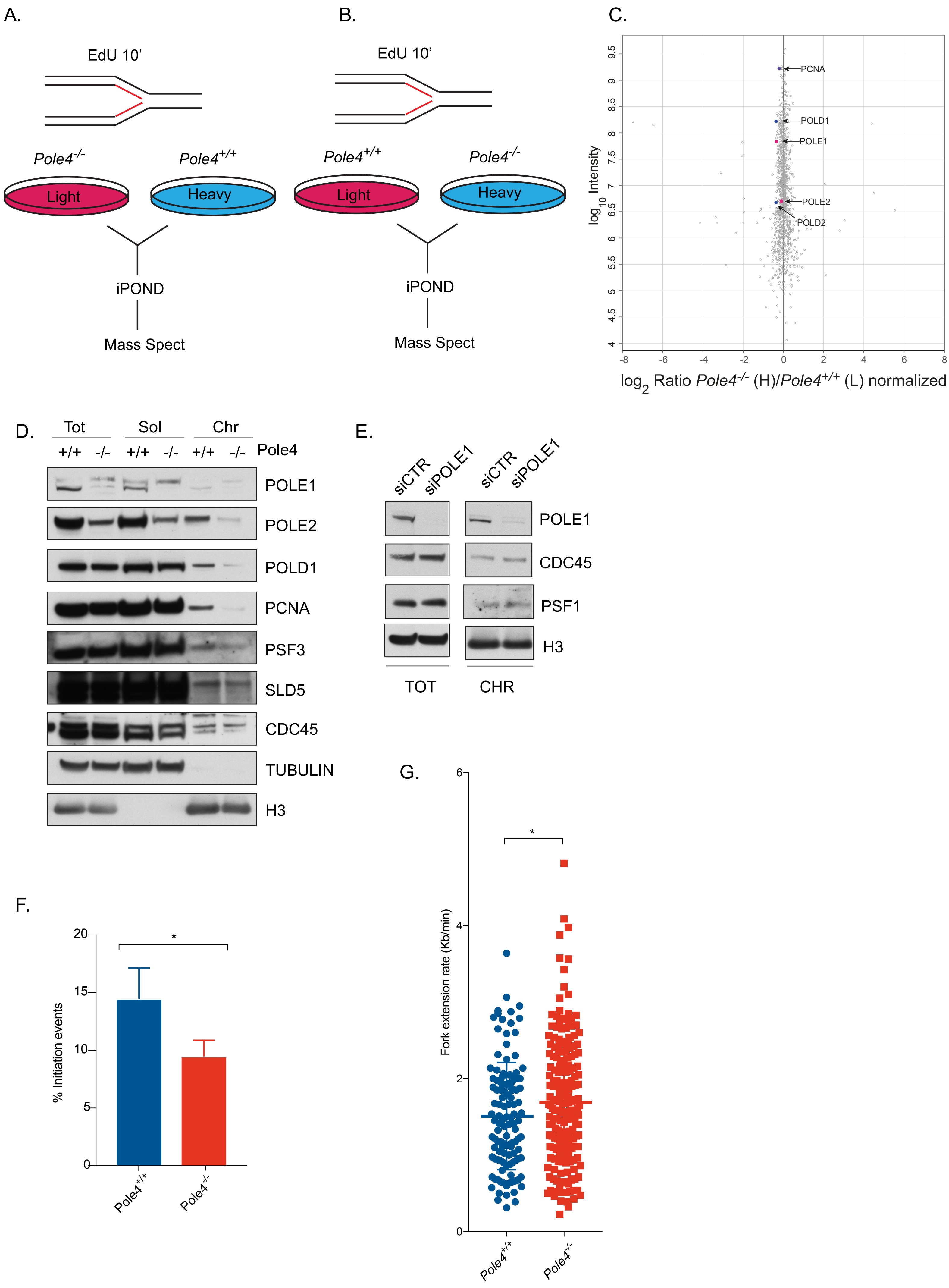
A.
EdU 10'
Pole4-/-
Pole4+/+
Light
Heavy
iPOND
Mass Spect
B.
EdU 10'
Pole4+/+
Pole4-/-
Light
Heavy
iPOND
Mass Spect
C.
log10 Intensity
PCNA
POLD1
POLE1
POLE2
POLD2
log2 Ratio Pole4-/- (H)/Pole4+/+ (L) normalized
D.
Tot
Sol
Chr
+/+ -/- +/+ -/- +/+ -/-
Pole4
POLE1
POLE2
POLD1
PCNA
PSF3
SLD5
CDC45
TUBULIN
H3
E.
siCTR
siPOLE1
siCTR
siPOLE1
POLE1
CDC45
PSF1
H3
TOT
CHR
F.
% Initiation events
*
Pole4+/+
Pole4-/-
G.
Fork extension rate (Kb/min)
*
Pole4+/+
Pole4-/-

Figure S4. (Related to Figure 3).

(A) and (B) Cartoons depicting the iPOND-SILAC-MS strategy used for replisome analysis of *Pole4*$^{-/-}$ and *Pole4*$^{+/+}$ MEFs (C) Results of the iPOND-SILAC-MS experiment reported as logarithmic fold change of Heavy/Light ratio. Polδ and Polε major subunits are indicated in the plot as blue and red dots respectively. (D) Western blot analysis of replication proteins from total, soluble and chromatin fractions of *Pole4*$^{+/+}$ and *Pole4*$^{-/-}$ MEFs obtained from inbred C57BL/6 embryos. Tubulin and Histone H3 were used as loading controls (E) Western blot analysis of replication proteins from total (TOT) and chromatin (CHR) fractions of HeLa cells transfected with siRNAs against POLE1. Histone H3 was used as loading control. (F) Bar graph showing the percentage of initiation events in *Pole4*$^{+/+}$ and *Pole4*$^{-/-}$ MEFs. Data were obtained from 3 different *Pole4*$^{+/+}$ and *Pole4*$^{-/-}$ MEFs clones (*$p<0.05$); Error bars ±standard deviation (SD) are included. (G) Replication fork speed (IdU extension rate) of *Pole4*$^{+/+}$ and *Pole4*$^{-/-}$ MEFs in a C57BL/6 genetic background (*$p<0.05$).

Figure S5

A.

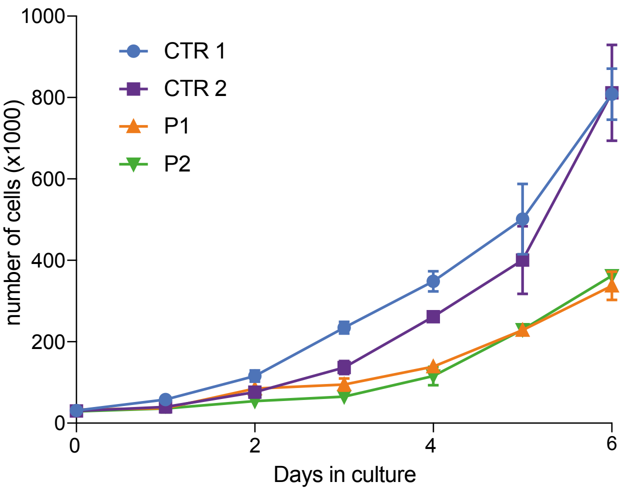

B.

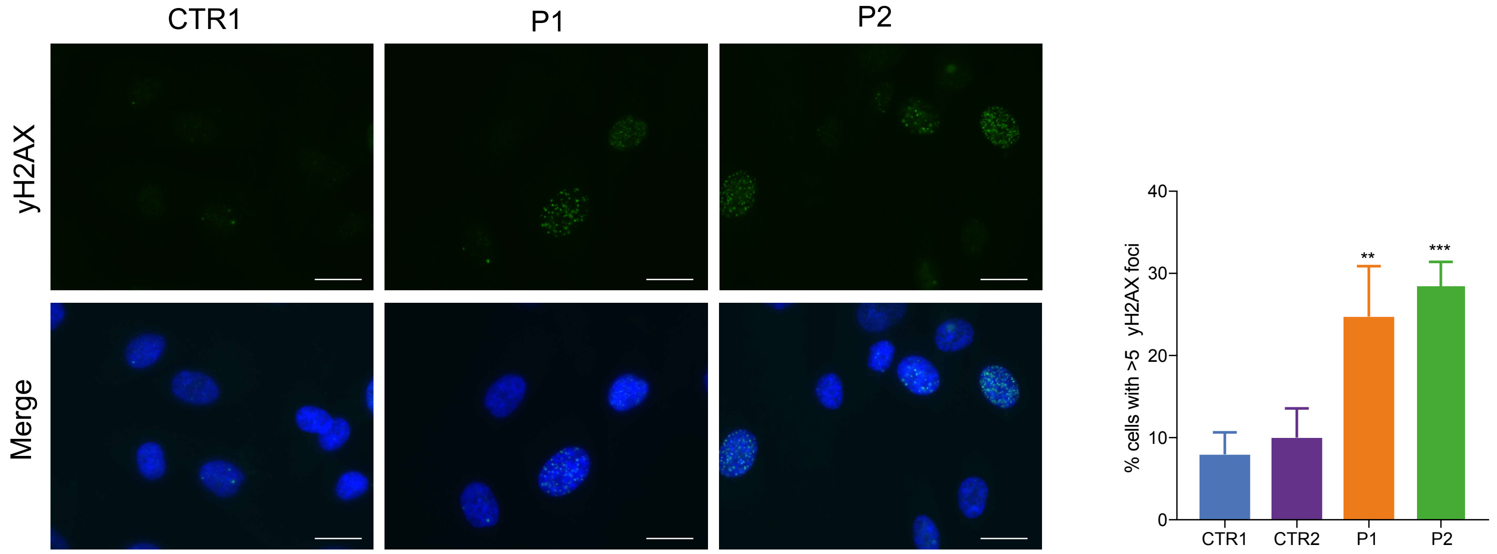

C.

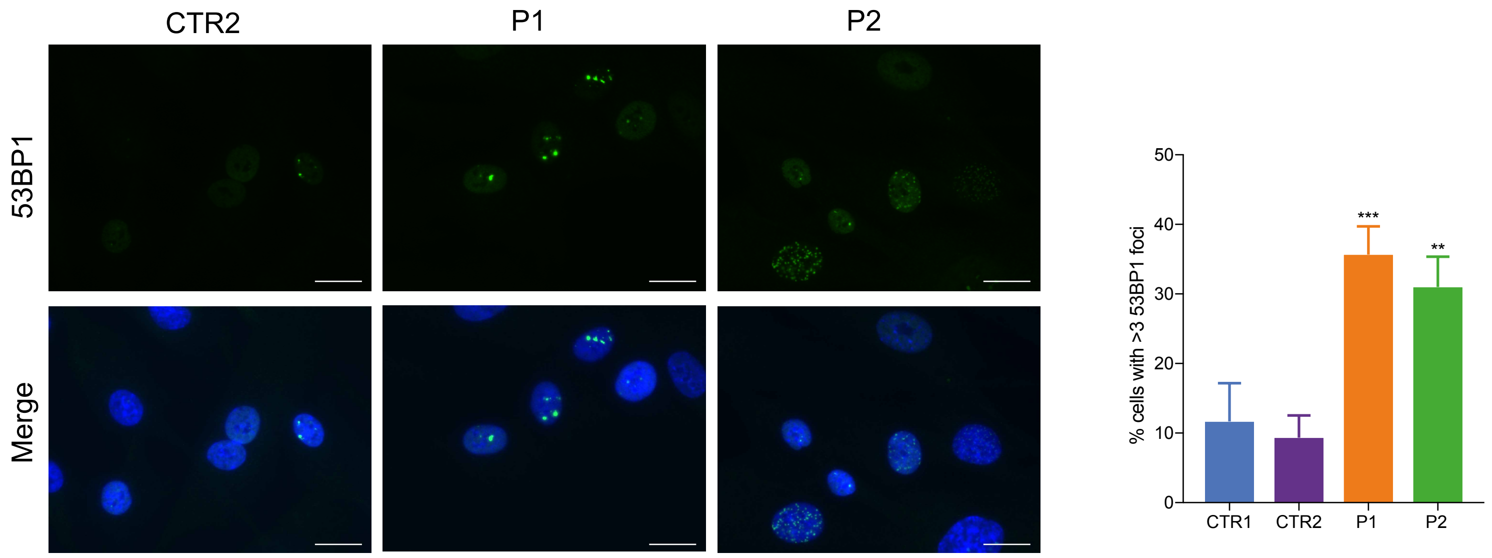

Figure S5. (Related to figure 4).

(A) Growth curve of *POLE1* mutant (P1 and P2) and control (CTR1 and CTR2) cells. 20.000 cells were plated in duplicate and counted for 6 consecutive days; Error bars ±standard deviation (SD) are included. (B) Left: representative γH2AX immunofluorescence staining of CTR1 and *POLE1* mutant (P1 and P2) cells. DAPI was used for nuclear counterstaining. Right: Bar graph showing percentage of CTR and *POLE1* mutant cells with >5 γH2AX foci (** $p < 0.01$; *** $p < 0.001$); Error bars ±standard deviation (SD) are included; Scale bar, 30μM. (C) Left: representative 53BP1 immunofluorescence staining of CTR1 and *POLE1* mutant (P1 and P2) cells. DAPI was used for nuclear counterstaining. Right: Bar graph showing percentage of CTR and *POLE1* mutant cells with >3 53BP1 foci (** $p < 0.01$; *** $p < 0.001$); Error bars ±standard deviation (SD) are included; Scale bar, 30μM.

Figure S6

A.

Genotype	Observed #	Observed %	Expected #	Expected %
Pole4$^{+/+}$ *p53*$^{+/+}$	5	4.6	6.8	6.25
Pole4$^{+/+}$ *p53*$^{+/-}$	20	18.3	13.6	12.5
Pole4$^{+/+}$ *p53*$^{-/-}$	3	2.8	6.8	6.25
Pole4$^{+/-}$ *p53*$^{+/+}$	14	12.8	13.6	12.5
Pole4$^{+/-}$ *p53*$^{+/-}$	43	39.4	27.3	25
Pole4$^{+/-}$ *p53*$^{-/-}$	9	8.3	13.6	12.5
Pole4$^{-/-}$ *p53*$^{+/+}$	0	0	6.8	6.25
Pole4$^{-/-}$ ***p53***$^{+/-}$	***11***	***10.1***	***13.6***	***12.5***
Pole4$^{-/-}$ ***p53***$^{-/-}$	***4***	***3.7***	***6.8***	***6.25***
Total	109	100	109	100

B.

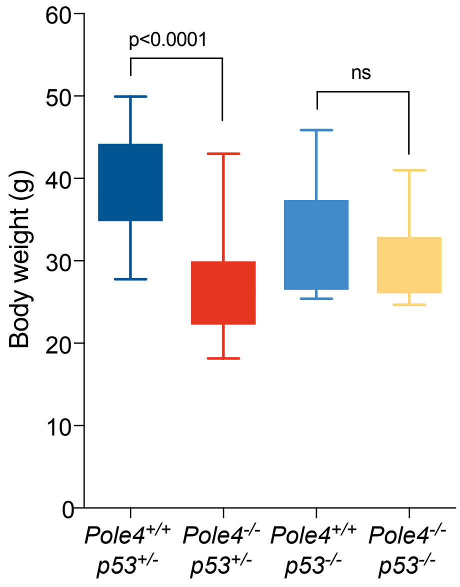

C.

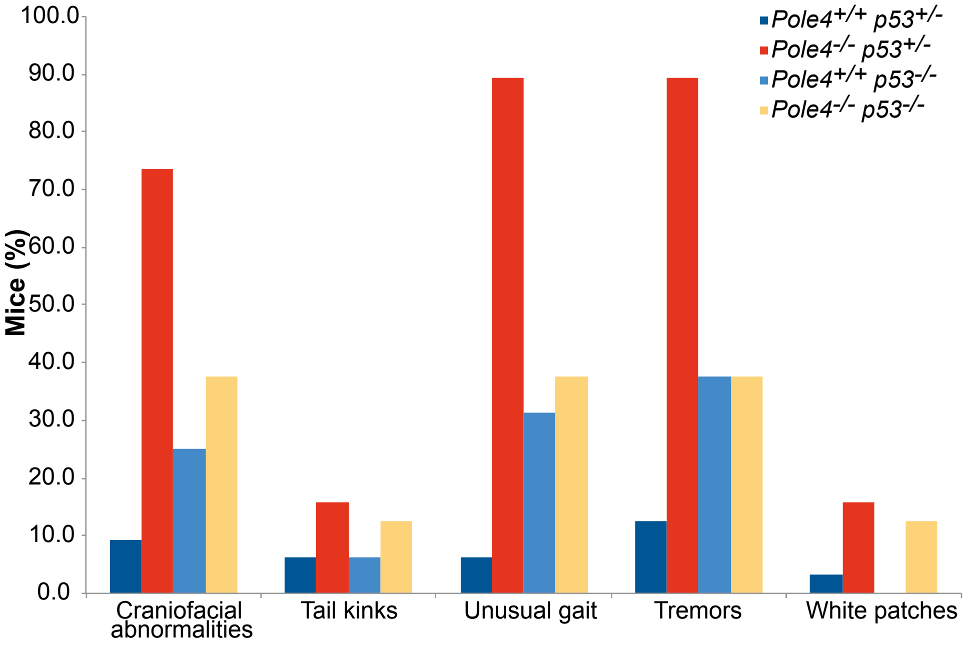

Figure S6: (Relative to Figure 6)

(A) *Pole4/p53* mice Mendelian ratios in C57BL/6 background. (B) *Pole4/p53* mice body weight at 6 months. Significance: *t*-test. (C) Frequency of *Pole4/p53* mice displaying abnormal phenotypes such as craniofacial abnormalities, tail kinks or curl, unusual gait, tremors and/or white patches.
